# Methotrexate’s effect on cells and adalimumab immunogenicity in axial spondyloarthritis: A mathematical study

**DOI:** 10.1371/journal.pcbi.1014740

**Published:** 2026-09-21

**Authors:** Sara Sottile, Conception Paul, Rachel Audo, Theo Rispens, Denis Mulleman, Peter Rashkov

**Affiliations:** 1 Department of Medical and Surgical Sciences, Università di Bologna, Bologna, Italy; 2 Institut de Génétique Moléculaire de Montpellier, Montpellier, France; 3 PhyMedExp, Université de Montpellier, CNRS, INSERM, Montpellier, France; 4 Department of Rheumatology, Hôpital Arnaud de Villeneuve, Centre Hospitalier Universitaire de Montpellier, Université de Montpellier, Montpellier, France; 5 Department of Immunopathology, Sanquin Research, Amsterdam, The Netherlands; 6 Landsteiner Laboratory, Amsterdam UMC, University of Amsterdam, Amsterdam, The Netherlands; 7 Nanomedicines and Nanoprobes Department, Center for Molecular Biophysics, UPR CNRS 4301, Université de Tours, Tours, France; 8 Service de Rhumatologie, Centre Hospitalier Régional Universitaire de Tours, Tours, France; 9 Institute of Mathematics and Informatics, Bulgarian Academy of Sciences, Sofia, Bulgaria; The University of British Columbia, CANADA

## Abstract

Axial spondyloarthritis is a chronic inflammatory disease impacting the spine and joints. Tumour necrosis factor inhibitors, like adalimumab, are used to treat severe cases, but up to 25% of patients discontinue due to reduced effectiveness, often from emergence of anti-drug antibodies. Methotrexate, while ineffective alone in treatment, has shown potential in reducing the formation of anti-drug antibodies to the therapeutic compound, but its mechanism of action on the immune response remains unclear. The objective of the study is to develop a mathematical model that describes the impact of methotrexate in reducing the immunogenicity of adalimumab in axial spondyloarthritis. Based on mathematical models established in the literature, we formulate a system of ordinary differential equations to describe the temporal dynamics of the immune cells (T and B lymphocytes), the therapeutic compounds in the study (adalimumab and methotrexate), and TNFα. The data used to calibrate the model are sourced from previously published in vitro experiments and a clinical trial which involved 110 patients who received adalimumab alone or adalimumab in combination with methotrexate, from whom adalimumab concentration, lymphocyte counts, and antibody titres collected along five visits during the course of treatment. A computational model is used to generate virtual patient cohorts reflecting the original data and to simulate immunogenic responses to adalimumab. Simulations across 31 scenarios for the mechanism whereby methotrexate acts on the immune cell subsets, predict that methotrexate likely reduces immunogenicity by increasing apoptosis of activated T cells.

## Author summary

We present a mathematical model that describes the impact of the disease-modifying drug methotrexate in suppressing immunogenicity of the tumour necrosis factor inhibitor adalimumab by describing the temporal dynamics of the immune response based on a study of patients with axial spondyloarthtitis. We study computationally 31 scenarios for the possible mechanism of action on different immune cell subsets via generated virtual patient cohorts reflecting the available data. Our simulations predict that methotrexate likely reduces immunogenicity by suppressing activated T cells. Our model’s predictions can help guide further experimental work to illuminate methotrexate’s mechanism of action in chronic inflammatory diseases, and improve quality of life for patients whose treatment is often interrupted due to development of anti-drug antibodies.

## 1 Introduction

Axial spondyloarthritis (AxSpA) is a chronic inflammatory disease that primarily affects the axial skeleton, which includes the spine and the sacroiliac joints. This condition is classified into two distinct forms: radiographic AxSpA, where structural damage is visible on X-ray images, and non-radiographic AxSpA, where no clear radiographic changes are seen, but other clinical or imaging features point to the presence of the disease. AxSpA typically begins in the third decade of life, with the disease more commonly affecting men than women, with a male-to-female ratio of approximately 2:1. The prevalence of AxSpA appears to align closely with the presence of the HLA-B27 gene, which contributes a substantial proportion of the disease susceptibility. The disease’s pathogenesis is also known to involve immune system factors such as tumour necrosis factor (TNFα) and interleukin-17 (IL-17), both of which play critical roles in the inflammation seen in AxSpA [1,2].

The first-line therapy is the use of non-steroidal anti-inflammatory drugs (NSAIDs), which help to reduce pain and inflammation [2,3]. In cases where NSAIDs are insufficient or in more severe cases of AxSpA, biopharmaceuticals/therapeutic antibodies such as TNFα inhibitors and IL-17 blockers are commonly used to target the underlying immune system dysfunction and manage inflammation [1,3].

Anti-drug antibodies (ADA) are immune responses that develop against therapeutic proteins, such as biopharmaceuticals used in the treatment of various conditions. The presence of ADA can significantly reduce the efficacy of TNFα inhibitors, which are commonly used to treat autoimmune or chronic inflammatory diseases [4–6]. The effect of ADA on drug concentration and clinical response depends on the magnitude to ADA titres. Higher ADA titres are more likely to cause therapeutic failure, while low levels of ADA may not interfere significantly with the treatment’s efficacy [7–9].

Methotrexate (MTX) is a disease modifying antirheumatic drug (DMARD) that is commonly used to treat chronic inflammatory and autoimmune conditions like rheumatoid arthritis, peripheral forms of spondyloarthritis, and psoriatic arthritis [10]. MTX’s anti-inflammatory action likely manifests in increase of extracellular adenosine that interacts with cell surface receptors and modulates the production of cytokines in rheumatoid arthritis [10,11], with effects being likely cell cycle-dependent [12]. In the context of rheumatoid arthritis, studies have shown that MTX co-medication is associated with a reduced proportion of patients who develop detectable ADA, suggesting that it may play a role in reducing the immune response against therapeutic compounds [13].

The goal of this study is to adapt existing mathematical models for immunogenicity [14,15] to the context of anti-TNFα therapy with the fully human IgG1 monoclonal antibody adalimumab (Humira) in the treatment of spondyloarthritis [9,13]. This model is mechanistic and describes the main mechanisms of immunogenicity: antigen presentation, lymphocyte activation and differentiation, formation of immune memory as results of the adaptive immune response, and production of ADA. It is calibrated using a 26-week prospective, randomised, open-labelled, multicentre study CoMARIS [9]. The model includes MTX co-medication in order to study possible modes of action that help reduce the immunogenicity of adalimumab [4,13].

Mechanistic mathematical models that examine the impact of methotrexate in inflammatory diseases are scarce [16]. Based on literature data for *in vitro* experiments with MTX, several modes of action on immune cell subsets are formulated as therapeutic responses, parametrised using data fitting, and used in the model of immunogenicity as modules that are selectively included or excluded to simulate different combinations. Parameter values are estimated from available data in CoMARIS or taken from the literature. The model’s modular structure allows combinations of methotrexate and adalimumab be implemented, and thus, it is a convenient tool to simulate different mechanisms for the action of MTX on various lymphocyte subpopulations. In this way the computational experiments help improve the understanding behind the reduced immunogenicity of adalimumab, thereby improving the efficacy of therapy. Virtual patient cohorts are generated across the different scenarios and simulated for lymphocyte dynamics and ADA production. The results for each scenario are then compared with the CoMARIS data set [9] to infer the most likely mechanism of action that suppresses the emergence of immunogenicity to adalimumab. At the end, we perform a simulation of the reactive co-administration of MTX, with delays ranging from 4 to 12 weeks.

Computational modelling enables the *in silico* study of biological systems under external controls (in this study, the therapy of AxSpA using therapeutic antibodies in co-administration of DMARD). While clinical data are invaluable, a real patient can obviously receive only one treatment at a time. In contrast, synthetic data generated from the same virtual patient allow testing multiple treatment scenarios on that individual. This capability enables the system’s output to be evaluated under various assumed treatment regimes and compared with observed data. This distinguishes *in silico* models from *in vivo* experimental work and helps guide further experimental research.

## 2 Materials and methods

### 2.1 Ethics statement

The patient study was approved by the ethics committee of Tours University Hospital and was conducted in accordance with the Declaration of Helsinki. It was registered at ClinicalTrials.gov (NCT01895764).

Patients signed an informed consent to participate in the clinical study and agreed their data to be used for research purposes by authorized persons in accordance with the law. In the informed consent, the patients declared they acknowledge their data could be analysed by authorised persons by the promoter and by persons mandated by the Health authorities.

### 2.2 Study design

Patients fulfilling the Assessment of Spondyloarthritis International Society criteria were recruited between March 2013 and October 2014 within the HUGO network (Hôpitaux Universitaires du Grand Ouest-Western France University Hospitals) were enrolled in CoMARIS, a 26-week prospective, randomised, open-labelled, multicentre study [9]. All patients received adalimumab subcutaneously every other week, and half of them were randomly assigned to receive MTX subcutaneously 2 weeks before adalimumab (week −2) as well as every week after that.

Data were collected from 110 patients, from whom measurements of adalimumab concentration and antibody titres were taken across five hospital visits (week 0, 4, 8, 12, and 26) [9]. Participants were drawn blood immediately prior to an adalimumab injection. However, 18 patients missed one or more visits, leaving a total of 91 patients with complete data used in the model calibration. Among these 91 patients, 49 received only adalimumab (whom we refer to as MTX-negative or MTX–), while 42 received adalimumab in combination with MTX (whom we refer to as MTX-positive or MTX+).

### 2.3 Biological analysis

ADA are detected with an antigen-binding test performed as described previously [7]. Antibody concentrations are compared with a standard serum containing ADA concentrations and expressed in arbitrary units per millilitre (AU/mL). A patient’s ADA-positive and ADA-negative status is classified respectively as titres above 12 and below 12 AU/mL at week 26. Taking into account all visits, ADA-low and ADA-high status is classified respectively as 13–100 and >100 AU/mL at any time as described previously [9].

Adalimumab concentration is measured by using a validated ELISA as indicated in [17]. Unfortunately, few TNFα measurements are detected at baseline and due to the small sample size, they are not used in the model calibration (Fig D in S1 Appendix). Concentration measurements of MTX and of its metabolites (MTX-polyglutamate) are not provided.

In addition to the antibody and adalimumab measurements, lymphocyte analysis was performed for a subset of the 91 patients. Blood samples were collected across several visits (week -2, 4, 12, and 26) from 20 patients to analyze B and T lymphocyte counts. 11 of the remaining 19 samples are from MTX– patients (of whom 3 were classified as ADA-low (2 male, 1 female), and 2 as ADA-high (both female)). Finally, 8 of the 19 samples are from MTX+ patients (of whom 7 were classified as ADA-negative and 1 as ADA-high (female)).

Peripheral mononuclear blood cells (PBMCs) were isolated from blood collected in EDTA tubes using the Ficoll density gradient method (Ficoll-Paque PLUS, density 1.077g - GE Healthcare) and frozen until T cells and B cells phenotyping by flow cytometry. After defrost of cells, blocking was performed by adding 25 μL of 1/20 human plasma to prevent non-specific antibody binding and then incubated for 20 minutes with fluorochrome conjugated antibodies as followed: for T phenotyping: anti-CD127- PerCpCy5.5, anti-CD25-PE and anti-CD4 FITC; for NK/T phenotyping: anti-CD3 APC, anti-CD4 FITC, anti-CD8 PerCpCY5.5, anti-CD16 BV510, anti-CD56 PE/Cy7; and for B phenotyping: anti-CD19-BV510, anti-CD24-PE, anti-CD27-PE/Cy7, anti-CD38 APC-H7, anti-IgD APC, anti-CD5-PERCPCY5.5, anti-IgM-FITC (all antibodies from BD Pharmingen). Dead cells were excluded by adding DAPI before acquisition on the FACSCanto II (Becton Dickinson).

### 2.4 Data description

In the group of 55 MTX– patients were 29 patients who developed ADA, three of whom had transient immunogenicity. Of these, 7 patients had high levels of antibodies in the final week 26. In contrast, only 14 patients in MTX+ cohort developed ADA, among whom 3 patients were classified as ADA-high.

From the patient visits data on several immune cell subsets, including naive T cells, identified as CD4 + CD25-, activated T cells (CD4 + CD25 + , however, we note that regulatory T cells also express the CD25 marker), NK cells (CD3-CD56+) as well as several subsets of B lymphocytes, including naive B cells (identified as CD19 + CD27-), memory B cells (CD19 + CD27+), and precursors to B regulatory (B10) cells (CD24^high^CD27 + , CD24^high^CD26^high^) are available for calibration of the mathematical model.

There were no data available on memory CD4 + T cells or effector T cells, and monocytes, including macrophages, dendritic cells, and plasma cells were not measured. Importantly, the CoMARIS data set includes total counts of these subsets of immune cells, and no analysis of their ability to specifically recognise adalimumab has been performed. Therefore, the available data have been used as indicator of total cell count. All biological analyses were performed after study completion without the knowledge of clinical data or group of randomisation.

A comparison of the two treatment cohorts reveals some notable differences. The ADA titres are lower in the MTX+ cohort, while the adalimumab concentration was higher in this group, including among those who developed ADA [9].

### 2.5 Formulation of the mathematical model

**Fig 1:**
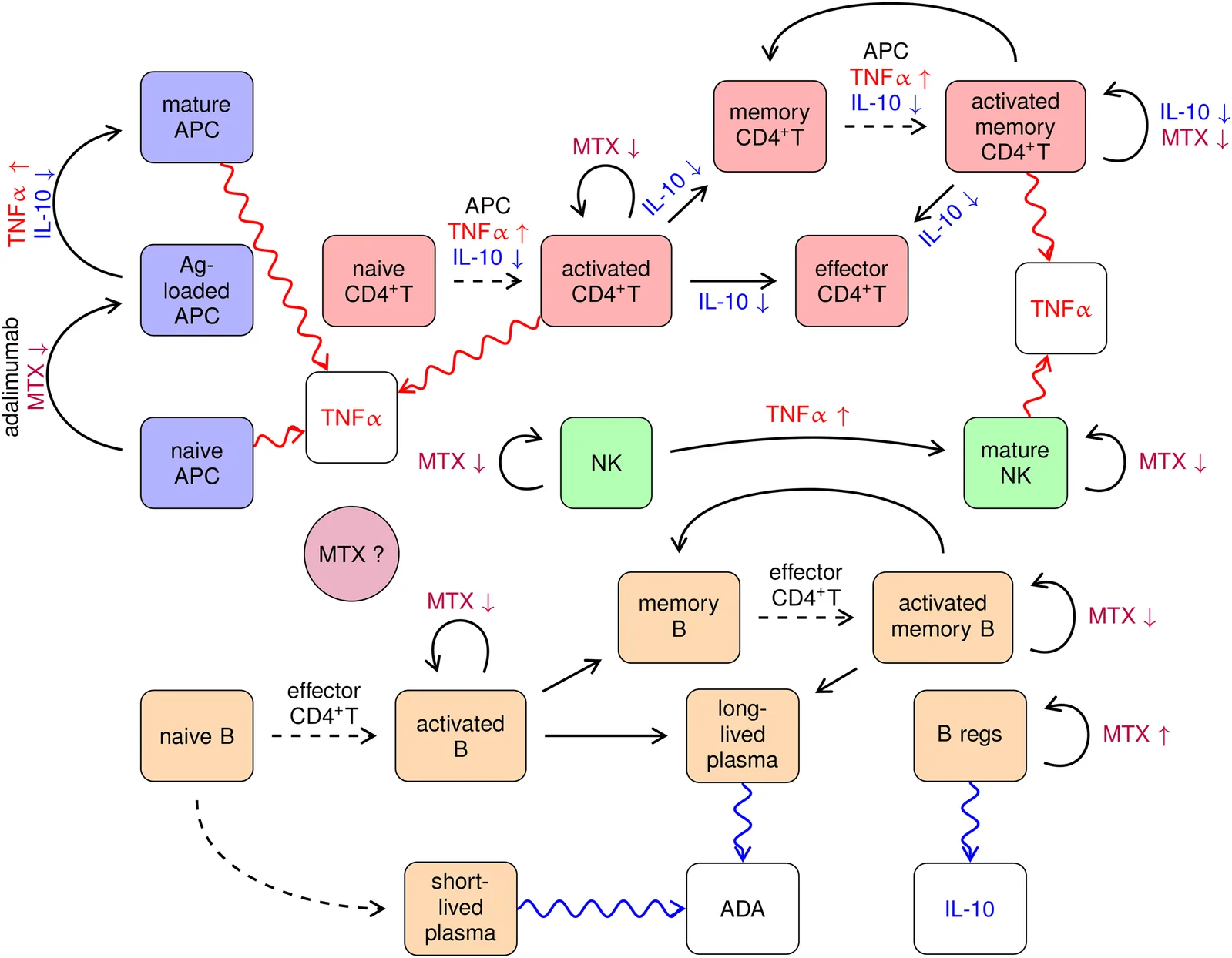
Scheme of the model for the interactions between APC, T lymphocytes, B lymphocytes, adalimumab and MTX. Dashed arrows denote activation, continuous arrows denote proliferation (if circling to the same compartment) or differentiation. Wavy arrows denote production of TNF-α, or IL-10, up- and down-arrows following TNF-α, MTX or IL-10 denote stimulation and suppression of the process.

We adapt a model for immunogenicity against therapeutic compounds [14] to the CoMARIS setting of anti-TNF-α therapy based on adalimumab, whereby two patient cohorts are considered – with or without co-administration of MTX. Our model’s structure is simplified compared to that of the original model because the CoMARIS data set does not contain data on MHC-II alleles, B- and T-cell epitopes or type of ADA against adalimumab. Nevertheless, we could include additional types of immune cells available for the CoMARIS data set (NK and B10 cells) in the model. The basic scheme of the model is shown in Fig 1 and the variables are listed in Table 1. A system of ordinary differential equations describes the dynamics of TNF-α, antigen-presenting cells (APC), CD4^+^ T lymphocytes, NK cells, B lymphocytes, adalimumab, MTX and antidrug antibody.

**Table 1:** List of model variables.

Variable	Notation	Unit
Adalimumab (depot)	*U*_0_(*t*)	μg/mL
Adalimumab (blood)	*U*(*t*)	μg/mL
Methotrexate (depot)	*X*_0_(*t*)	μg/mL
Methotrexate (blood)	*X*(*t*)	μg/mL
Tumour necrosis factor α (TNF-α)	$F_{\alpha }(t)$	μg/mL
Antidrug antibody	*A*(*t*)	AU/mL (μg/mL)
Immature/resting APC	*D*_0_(*t*)	cells/mL
Naive CD4 + T cells	*H*_0_(*t*)	cells/mL
Memory T cells	$H_{m}(0)$	cells/mL
Activated naive CD4 + T cells	$H_{a}(t)$	cells/mL
Activated memory CD4 + T cells	$H_{ma}(t)$	cells/mL
Effector T cells	$H_{e}(t)$	cells/mL
Naive B cells	*B*_0_(*t*)	cells/mL
Activated B cells	$B_{a}(t)$	cells/mL
Memory B cells	$B_{m}(t)$	cells/mL
Activated memory B cells	$B_{ma}(t)$	cells/mL
NK cells	*K*_0_(*t*)	cells/mL
Mature NK cells	*K*_1_(*t*)	cells/mL
Short-lived plasma cells	$P_{s}(t)$	cells/mL
Long-lived plasma cells	*P*(*t*)	cells/mL
B10 (B regulatory) cells	*B*_10_(*t*)	cells/mL

#### 2.5.1 PK models (adalimumab, methotrexate, TNF-α, ADA).

For the therapeutic monoclonal antibody adalimumab and for MTX we employ a two-compartment pharmacokinetic model (depot-central compartment). Adalimumab is administered in the depot (site of injection) as a pulse at a regular interval $\tau_{U}=14\;\text{days}$ in equation (1) starting from week 0 (*t* = 0 days). Its uptake dose 40 mg corresponds to a concen*t*ration of $D_{U}=50$ mg/mL or 50000 μg/mL. Adalimumab is absorbed into the blood at rate $a_{U}$, and cleared from the blood at rate $\delta_{U}$.

The volume ratio between the two compartments, denoted by $V_{U}$, is estimated from the CoMARIS data, as shown in Table B in S1 Appendix. Adalimumab binds to its target, TNF-α at rate $k_{100}UF_{\alpha }$, or to the antidrug antibody (ADA) at rate *k*_101_ *A U*. However, as the binding to its target has a negligible effect on the adalimumab concentration, with $U(t)\gg F_{\alpha }(t)$ [4] and TNF-α barely measured at baseline in the CoMARIS data set, we neglect the term $-k_{100}UF_{\alpha }$ in the equation (2)


$$\begin{array}{c} U_{0}^{\prime }=D_{U}\sum_{j=0}^{n}\delta (t-\tau_{U}j)-a_{U}U_{0},\;U_{0}(0)=0 \end{array}$$ (1)

$$\begin{array}{c} U^{\prime }=a_{U}\frac{U_{0}}{V_{U}}-k_{101}AU-\delta_{U}U,\;U(0)=0. \end{array}$$ (2)

Similarly, MTX (10 mg) is co-administered in the depot with an initial dose of $D_{X,1}=10$ mg/mL at t=−14 days, followed by subsequent injections at an interval of $\tau_{X}=7$ days from day 0 as described [9]. MTX is absorbed into the blood at rate $a_{X}$, and cleared from the blood at rate $\delta_{X}$ (equations (3)-(4)).


$$\begin{array}{c} X_{0}^{\prime }=\left(D_{X}\delta (t+2\tau_{X})+\sum_{j=0}^{n}D_{X}\delta (t-j\tau_{X})\right)-a_{X}X_{0},\;X_{0}(-14)=0 \end{array}$$ (3)

$$\begin{array}{c} X^{\prime }=a_{X}\frac{X_{0}}{V_{X}}-\delta_{X}X,\;X(-14)=0\;. \end{array}$$ (4)

Free antidrug antibody (ADA, equation (5)) is produced by both short-lived and long-lived plasma cells at a rate $p_{A}$ and undergoes clearance at rate $\delta_{A}$.


$$\begin{array}{c} A^{\prime }=p_{A}(P_{s}+P)-\delta_{A}A,\;A(0)=0. \end{array}$$ (5)

Levels of ADA against adalimumab were reported also in other inflammatory diseases (for IBD the cut-off value for undetectable antidrug antibodies is 10 ng/mL [18]).

TNF-α is a pro-inflammatory cytokine produced by various cell types: neutrophils, macrophages, dendritic cells, activated CD4 + T cells, NK cells [6,19,20], which modulates the inflammatory process and the immune response. Reported levels of TNF-α in AxSpA are in the range 13.24±7.45 pg/mL [21,22], but as already mentioned, sufficient data on TNF-α is not available in CoMARIS (Fig D in S1 Appendix). In our model we assume a term $\kappa_{0}$ for the background production of TNFα, and specific production rates $\kappa_{1},\kappa_{3},\kappa_{5}$ are given for TNFα production by APCs, activated CD4 ^+^ T lymphocytes and mature CD3-CD56 + CD16 + NK cells respectively. TNFα undergoes decay at rate $\delta_{\alpha }$, as well as inhibition by the binding to anti-TNFα monoclonal antibody adalimumab, as reflected in the last term $-k_{100}UF_{\alpha }$ of equation (6).


$$\begin{array}{c} F_{\alpha }^{\prime }=\kappa_{0}+\kappa_{1}(D_{0}+D_{1})+\kappa_{3}(H_{a}+H_{ma})+\kappa_{5}K_{1}-\delta_{\alpha }F_{\alpha }-k_{100}UF_{\alpha }\;. \end{array}$$ (6)

TNFα acts on different immune cell populations in a dose-dependent manner which we model as a Michaelis-Menten term with EC50 $\kappa_{TNF}$, and maximum rate depending on the context.

#### 2.5.2 Antigen-presenting cells.

Antigen-presenting cells (APC) are crucial for the process of immunogenicity because they pick, process and display antigen peptides on their MHC-II complexes to activate the adaptive immunity. Unfortunately, APCs were not measured in CoMARIS since APCs localise in lymph nodes, tissues and sites of inflammation. As proxy for APC counts we use a combination of dendritic cells (DCs) and macrophages. DCs always express MHC-II, but unlike DCs, macrophages do not always express MHC-II. Only after IFNγ-induced activation these cells synthesise MHC-II.

At homeostasis for MTX-negative patients, resting APCs are produced at a rate equal to their removal rate $\mu_{D}$ and have a carrying capacity $K_{D}$ (7). Resting APCs uptake of antigen (adalimumab) at rate $\alpha_{APC|U}D_{0}U$ and become antigen-loaded APCs $D_{*}$ (8) before they enter the mature APC stage *D*_1_. TNFα is important for maturation and survival of DCs as evidenced by impaired functionality of DCs derived from rheumatoid arthritis patients on anti-TNF therapy [23] and by NF-κB activation post TNFα stimulation [24]. In (8) we model the TNFα -mediated pro-maturation signal as an increase of the maturation rate $\alpha_{1}$ by up to 50% for high levels of TNFα. In (9) we model the TNFα-induced survival of APCs as a reduction in the removal rate of mature APCs $\frac{\mu_{D}}{1+\frac{F_{\alpha }}{\kappa_{TNF}+F_{\alpha }}}$ [25], so that at high levels of TNFα the removal rate can be reduced by up to 50%.

Experimental data [10,26,27] reveal that MTX exerts an inhibitory effect on monocytes and macrophages. The suppressive action of MTX on APC activation is included in the equations for resting APCs in a dose-dependent manner at rate $\gamma_{1}X$. Finally, the suppressive action of IL-10 on the maturation of APCs (for example, on dendritic cells [28,29]) is included indirectly using the relative change in B regulatory (B10) cell population, via a proxy term $\frac{1}{c_{10}}$ in (9), where $c_{10}=B_{10}(t)/B_{10}(0)$. This is a reasonable assumption that does not require much additional complexity in the model because B10 lymphocytes secrete rapidly large amounts of IL-10, and are able to downregulate the immune response in inflammatory diseases solely via IL-10 [30]. Even though other immune cell populations such as monocytes or Th2 cells produce IL-10, this response may be context dependent and the lack of data on IL-10 or Th2 cells from CoMARIS makes the assessment of additional sources difficult to quantify.


$$\begin{array}{c} D_{0}^{\prime }=\mu_{D}(K_{D}-D_{0})-\alpha_{APC|U}D_{0}U-\gamma_{1}XD_{0},\;D_{0}(0)=K_{D} \end{array}$$ (7)

$$\begin{array}{c} D_{*}^{\prime }=\alpha_{APC|U}D_{0}U-\frac{\alpha_{1}}{c_{10}}(1+\frac{F_{\alpha }}{F_{\alpha }+\kappa_{TNF}})D_{*},\;D_{*}(0)=0 \end{array}$$ (8)

$$\begin{array}{c} D_{1}^{\prime }=\frac{\alpha_{1}}{c_{10}}(1+\frac{F_{\alpha }}{F_{\alpha }+\kappa_{TNF}})D_{*}-\frac{\mu_{D_{1}}}{1+\frac{F_{\alpha }}{\kappa_{TNF}+F_{\alpha }}}D_{1},\;D_{1}(0)=0 \end{array}$$ (9)

#### 2.5.3 CD4 + T lymphocytes.

The activation of naive and memory CD4^+^ T cells depends on the relative abundance of APCs available due to the limited number of T lymphocytes with which an APC can react at a given time. We use saturation terms of the form proposed by [14] to model this assumption: $\frac{D_{1}}{D_{1}+h_{n}H_{\sum }}$ for the activation of naive CD4^+^ T cells in equation (11) and $\frac{D_{1}}{D_{1}+h_{m}H_{\sum }}$ for the activation of memory CD4^+^ T cells in equation (13). There we denote $H_{\sum }(t)=H_{0}(t)+H_{a}(t)+H_{e}(t)+H_{m}(t)+H_{ma}(t)$, and the weights $h_{n},h_{m}$ in the denominator satisfy $h_{m}\leq h_{n}$ to take into account different activation thresholds between naive and memory CD4^+^ T cells, similar to [14].

Activated CD4 + cells $H_{a}$ and activated memory CD4 + T cells $H_{ma}$ can undergo several pathways: they can a) proliferate with probability *q*, or b) differentiate either to memory T cells or to effector T cells $H_{e}$ with probability respectively *p* or 1−p. The proliferation of activated T cells $H_{a}$ and $H_{ma}$ occurs at maximum rates $\rho_{Ha},\rho_{Hm}$. In the case of transient immunogenicity we let $\rho_{Hm}=0$, whereas $\rho_{Ha}>0$.

To allow for immune memory effects we assume logistic growth in equation (12) for memory CD4 ^+^ T cells $H_{m}$ with growth rate


$$\begin{array}{c} \alpha_{H_{m}}=\frac{\alpha_{T_{m}|APC}}{c_{10}}\left(1+\frac{F_{\alpha }}{\kappa_{TNF}+F_{\alpha }}\right) \end{array}$$

chosen to be equal to the maximum activation rate of memory CD4^+^ T cells by APCs. We note that other mathematical approaches are possible to maintain immune memory for longer periods of time, for example, in a model for the immune response to influenza [15].

In subsequent simulations we set *p* = 0.5 except for the case of restored immune tolerance (transient immunogenicity, where no memory T cells form).

We also include the antagonistic effects of cytokines in the activation rates. On the one hand, the anti-apoptotic action of TNFα on CD4 + T helper cells [6,31–33] is modelled as a reduction in the respective removal rates $\mu_{H0},\mu_{Ha}$ by up to 50% at high concentration of TNFα. Further, the increased activation rate due to pro-inflammatory TNFα [33,34] is modelled as an increase of up to 50% of the maximum activation rates $\alpha_{T|APC},\alpha_{Tm|APC}$ at high at high concentration of TNFα. On the other hand, IL-10 has a suppressive effect on activation, proliferation and differentiation from naive to T helper cells [33–35], and this is modelled by a reduction of the maximum activation rates $\alpha_{T|APC},\alpha_{Tm|APC}$ and the maximum proliferation rates $\rho_{Ha},\rho_{Hm}$ by a factor $\frac{1}{c_{10}}$ in (11) and (13) as a proxy for the immunosuppressive action of IL-10, similar to equation (8).

Finally, MTX has a dose-dependent apoptotic effect on the activated T cells only (experimental evidence provided in [36]) which we model through a Hill function $-\frac{\gamma_{4}X^{2}}{c_{4}^{2}+X^{2}}H_{a}$ and $-\frac{\gamma_{4}X^{2}}{c_{4}^{2}+X^{2}}H_{ma}$.


$$\begin{array}{c} H_{0}^{\prime }=-\frac{\alpha_{T|APC}}{c_{10}}\frac{D_{1}}{D_{1}+h_{n}H_{\sum }}(1+\frac{F_{\alpha }}{F_{\alpha }+\kappa_{TNF}})H_{0}\;, \end{array}$$ (10)

$$\begin{array}{cc} H_{a}^{\prime } & =\frac{\alpha_{T|APC}}{c_{10}}\frac{D_{1}}{D_{1}+h_{n}H_{\sum }}(1+\frac{F_{\alpha }}{F_{\alpha }+\kappa_{TNF}})H_{0}\\ & \;+\frac{q}{c_{10}}\frac{\rho_{Ha}D_{1}}{D_{1}+h_{n}H_{\sum }}H_{a}-\frac{\mu_{Ha}}{1+\frac{F_{\alpha }}{\kappa_{TNF}+F_{\alpha }}}H_{a}-\frac{\gamma_{4}X^{2}}{c_{4}^{2}+X^{2}}H_{a}\;, \end{array}$$ (11)

$$\begin{array}{cc} H_{m}^{\prime } & =\frac{p(1-q)}{c_{10}}\left(H_{a}\frac{\rho_{Ha}D_{1}}{D_{1}+h_{n}H_{\sum }}+H_{ma}\frac{\rho_{Hm}D_{1}}{D_{1}+h_{m}H_{\Sigma }}\right)\\ & \;-\frac{\alpha_{T_{m}|APC}}{c_{10}}\frac{D_{1}}{D_{1}+h_{m}H_{\sum }}(1+\frac{F_{\alpha }}{F_{\alpha }+\kappa_{TNF}})H_{m}+\alpha_{Hm}H_{m}(1-\frac{H_{m}}{K_{Hm}})\;, \end{array}$$ (12)

$$\begin{array}{cc} H_{ma}^{\prime } & =\frac{\alpha_{T_{m}|APC}}{c_{10}}\frac{D_{1}}{D_{1}+h_{m}H_{\sum }}(1+\frac{F_{\alpha }}{F_{\alpha }+\kappa_{TNF}})H_{m}\\ & \;+\frac{q}{c_{10}}\frac{\rho_{Hm}D_{1}}{D_{1}+h_{m}H_{\sum }}H_{ma}-\frac{\mu_{Ha}}{1+\frac{F_{\alpha }}{\kappa_{TNF}+F_{\alpha }}}H_{ma}-\frac{\gamma_{4}X^{2}}{c_{4}^{2}+X^{2}}H_{ma} \end{array}$$ (13)

$$\begin{array}{c} H_{e}^{\prime }=\frac{\left(1-p)(1-q\right)}{c_{10}}(\frac{\rho_{Ha}D_{1}}{D_{1}+h_{n}H_{\sum }}H_{a}+\frac{\rho_{Hm}D_{1}}{D_{1}+h_{m}H_{\sum }}H_{ma})-\frac{\mu_{He}}{1+\frac{F_{\alpha }}{\kappa_{TNF}+F_{\alpha }}}H_{e}\;. \end{array}$$ (14)

with initial conditions


$$\begin{array}{c} H_{0}(0)={\bar{H}}_{0},\;H_{a}(0)=0,\;H_{m}(0)=0,\;H_{ma}(0)=0,\;H_{e}(0)=0\;. \end{array}$$

Observe from equations (11), (12), (13) that the parameters $\alpha_{T|APC},c_{10}$ are not simultaneously identifiable.

#### 2.5.4 Natural killer (NK) cells.

NK cells are characterised as CD3^-^CD56^+^, and they produce cytokines like TNFα, IFN-γ, which prime CD4^+^ T cells and stimulate maturation of APCs [37]. We denote by *K*_0_ the total count of CD3^-^CD56^+^ NK cells and by *K*_1_ the mature cytotoxic NK cells under inflammatory conditions (identified as CD3^-^CD56^+^CD16^+^ NK cells [38,39].). In homeostasis NK cells are removed at rate $\mu_{NK}$. TNFα stimulates maturation of naive NK cells [40]. The maturation rate of NK cells $\alpha_{2}$ increases as function of TNFα in a dose-dependent manner.

Methotrexate has been reported to suppress NK cell activity in an *in vivo* murine model [41], downregulate gene expression in CD56 NK cells [42], as well as lower counts of NK cells and in human patients with rheumatoid arthritis treated with MTX compared to healthy subjects and MTX-naive patients [43]. Data from CoMARIS also indicate MTX leads to a reduction in NK cell counts, which we model as a suppressive effect on the two populations $K_{0},K_{1}$ in equation (15). The dynamics of TNFα-modulated activation of NK cells is described by


$$\begin{array}{cc} K_{0}^{\prime } & =\mu_{NK}(K_{NK}-K_{0})-\gamma_{2}XK_{0}\;,\\ K_{1}^{\prime } & =\alpha_{2}\left(1+\frac{F_{\alpha }}{F_{\alpha }+\kappa_{TNF}}\right)(K_{0}-K_{1})-\mu_{NK}K_{1}-\gamma_{3}XK_{1}\;. \end{array}$$ (15)

In the MTX– cohort we assume that the NK cell populations do not change between weeks −2 and 0, so we set $K_{NK}=10^{5}$ cells/mL, with *K*_1_(0) = 60000 cells/mL, while in the MTX^+^ group we set $K_{NK}=1.2\times 10^{5}$ cells/mL, with $K_{1}(-14)=84000$ cells/mL.

If the two populations are in equilibrium at *t* = 0, the value of $\alpha_{2}$ can be estimated by


$$\begin{array}{c} \alpha_{2}=\frac{\mu_{NK}}{1+\frac{F_{\alpha }}{F_{\alpha }+\kappa_{TNF}}}\frac{K_{1}(0)}{K_{0}(0)-K_{1}(0)}\;. \end{array}$$

#### 2.5.5 B lymphocytes.

For the B lymphocyte dynamics we adapt the model in [14] to model the process of the T cell-dependent activation, and to keep the model structure simple and due to data availability we do not model explicitly the binding of antigen to B cell receptor as proposed by [14].

We take the value of the initial condition $K_{B}$ for (16) in the range of 1000–5000 cells/mL since initially a small number of naive CD19^+^CD27^-^ cells would be available for antigen-presentation by effector CD4^+^ T lymphocytes. We choose this cell type to represent naive B cells in the model, since the expression of the CD27 marker is presumably essential for the formation of memory B cells [44,45].

Naive and memory B cells are activated in a T cell-dependent manner by CD4 + T effector cells with a maximum rate $\alpha_{B|T},\alpha_{Bm|T}$. For the T cell-dependent activation of naive and memory B cells we make a similar assumption as we did before for the activation of CD4^+^ T cells. The activation rate depends on the relative abundance of effector T cells $H_{e}(t)$ available due to the limited number of B cells to which an effector T cell can bind at any given time. We use saturation terms of the form proposed by [14] to model this assumption: $\frac{H_{e}}{H_{e}+b_{n}B_{\sum }}$ for the activation of naive B cells in equation (16) and $\frac{H_{e}}{H_{e}+b_{m}B_{\sum }}$ for the activation of memory B cells in equation (18). Here, the notation $B_{\sum }$ stands for the total amount of naive B cells, activated naive B cells, memory B cells and activated memory B cells:


$$\begin{array}{c} B_{\sum }=B_{0}(t)+B_{a}(t)+B_{m}(t)+B_{ma}(t)\;. \end{array}$$

The weights $b_{m},b_{n}$ satisfy $b_{m}\leq b_{n}$ to take into account the fact that effector CD4^+^ T cells activate memory B cells more efficiently than naive B cells [14] for the ADA-high patient cohort. For the ADA-low patient cohort, those values are assumed to be similar in magnitude because of the assumed lower number of T memory cells overall.

The rates of removal of activated B cells is given by $\mu_{Ba},\mu_{Bma}$ respectively in (17) and (19). Activated naive B cells become short-lived plasma cells $P_{s}$ at constant rate $\rho_{Ps}$, whose value may depend on the specific patient cohort. We assume that activated memory B cells are not able to differentiate into short-lived plasma cells.

We introduce parameters r,s∈(0,1) to describe the fates of activated naive and memory B cells $B_{a},B_{ma}$. These cells can a) proliferate with probability *s* or b) differentiate into memory B cells $B_{m}$ with probability *r*, or into long-lived antibody-secreting plasma cells *P* with probability (1−r). Activated B cells proliferate in a T cell-dependent manner at maximum rate $\rho_{Ba}$, and activated B memory cells proliferate in a T cell-dependent manner at maximum rate $\rho_{Bm}$. When s≈1, few B lymphocytes differentiate into B memory cells, and when r≈1, few activated B lymphocytes differentiate into long-lived plasma cells. In subsequent simulations we set *r* = 0.5 except for the case of restored immune tolerance (transient immunogenicity, where immune memory does not form in the form of B memory cells).

In the equation for memory B cells (18) we introduce a logistic term with growth rate $\alpha_{Bm|T}$ to account for the maintenance of immune memory over long periods of time. Finally, MTX has a dose-dependent apoptotic effect on the activated B cells only (experimental evidence provided in [12]) which we model through linear rates $-c_{40}XB_{a}$ and $-c_{41}XB_{ma}$. The dynamics of B lymphocytes is described by


$$\begin{array}{c} B_{0}^{\prime }=-\alpha_{B|T}\frac{H_{e}}{H_{e}+b_{n}B_{\sum }}B_{0}, \end{array}$$ (16)

$$\begin{array}{c} B_{a}^{\prime }=\alpha_{B|T}\frac{H_{e}}{H_{e}+b_{n}B_{\sum }}B_{0}+s\rho_{Ba}\frac{H_{e}}{H_{e}+b_{n}B_{\sum }}B_{a}-(\mu_{Ba}+\rho_{P_{s}})B_{a}-c_{40}XB_{a}, \end{array}$$ (17)

$$\begin{array}{cc} B_{m}^{\prime } & =r(1-s)[\rho_{Ba}\frac{H_{e}}{H_{e}+b_{n}B_{\sum }}B_{a}+\rho_{Bm}\frac{H_{e}}{H_{e}+b_{m}B_{\sum }}B_{ma}]\\ & \;-\alpha_{B_{m}|T}\frac{H_{e}}{H_{e}+b_{m}B_{\sum }}B_{m}+\alpha_{B_{m}|T}B_{m}\left(1-\frac{B_{m}}{K_{Bm}}\right), \end{array}$$ (18)

$$\begin{array}{c} B_{ma}^{\prime }=\alpha_{B_{m}|T}\frac{H_{e}}{H_{e}+b_{m}B_{\sum }}B_{m}+s\rho_{Bm}\frac{H_{e}}{H_{e}+b_{m}B_{\sum }}B_{ma}-\mu_{Ba}B_{ma}-c_{41}XB_{a}\;. \end{array}$$ (19)

with initial conditions


$$\begin{array}{c} B_{0}(0)={\bar{B}}_{0},\;B_{a}(0)=0,\;B_{m}(0)=0,\;B_{ma}(0)=0\;B_{a}(0)=0\;. \end{array}$$

#### 2.5.6 Short-lived plasma cells.

Short-lived plasma cells are primarily found in secondary lymphoid organs and serve as the initial producers of antibodies during an immune response. In contrast, long-lived plasma cells are generally located in the bone marrow, where they sustain antibody production for months to years. The role of short-lived plasma cells is important for the case of transient immunogenicity (observed in some ADA-low patients), whereby antibodies are formed initially, but whose production is not maintained over time. Short-lived plasma cells are formed by activated naive B cells and are removed at rate $\mu_{Ps}$.


$$\begin{array}{c} P_{s}^{\prime }=\rho_{P_{s}}B_{a}-\mu_{Ps}P_{s},\;P_{s}(0)=0 \end{array}$$

#### 2.5.7 Long-lived plasma cells.

The long-lived plasma cells *P* are essential for the immune memory. They are able to produce antibodies over long periods of time. They are formed from activated naive and memory B cells with probability (1−r)(1−s).


$$\begin{array}{c} P^{\prime }=(1-r)(1-s)[\rho_{Ba}\frac{H_{e}}{H_{e}+b_{n}B_{\sum }}B_{a}+\rho_{Bm}\frac{H_{e}}{H_{e}+b_{m}B_{\sum }}B_{ma}]-\mu_{P}P,\;P(0)=0\;. \end{array}$$ (20)

#### 2.5.8 B10 cells.

B10 (or B regulatory, Breg) cells produce IL-10 and act as regulators of immune response [30]. They are known for actively secreting IL-10 and can influence the immune response in multiple ways: by suppressing TNFα responses *in vitro*, IFN γ responses *in vitro* and *in vivo*, CD4^+^ T cell activation, dendritic cell antigen presentation and antigen-specific CD4^+^CD25^+^ proliferation [46]. Studies suggest that on one hand, MTX leads to increased interleukin-10 gene expression in lymphocytes *in vitro* [47] and, on the other, MTX-induced immune tolerance induces a specific expansion of IL-10- and TGF-β-secreting B cells that express Foxp3, which implies the induction of B regulatory cells [11,48]. CoMARIS data (Fig C in S1 Appendix) demonstrate that the abundance of CD24^high^CD27 + cells, which are able to differentiate into B10 cells [49], is stimulated in the MTX+ cohort.

We use the following model to describe the stimulatory action of MTX on B10 cell population:


$$\begin{array}{c} B_{10}^{\prime }=\mu_{10}(K_{10}+\alpha_{10}K_{10}X-B_{10}),\;B_{10}(0)>0\;. \end{array}$$ (21)

The mathematical model developed and presented here consists of a relatively large number of equations (23 in total), while only a few of the parameters involved can be directly found in the literature. In addition, many of these parameters are not simultaneously identifiable if one attempts to estimate them from available data. To overcome this limitation, we employ simpler, lower-dimensional auxiliary models—typically involving only two or three variables to estimate specific subsets of parameters. These models are presented in Section C in S1 Appendix. The resulting parameter values were then integrated back into the full model, ensuring consistency between the simplified and comprehensive formulations. The values of the model parameters are listed in Section D in S1 Appendix. These parameters are chosen to calibrate the model output representing virtual patients whose status is classified as ADA-low (including ADA-transient) and ADA-high according to the criteria from [9].

Our mathematical model involves a large number of parameters, and many of them have been estimated using simpler auxiliary models. Given the model’s complexity, it is essential to assess how variations in individual parameters influence the outputs of interest ADA titre *A* and adalimumab concentration *U*.

Sensitivity analysis is a mathematical technique used to determine how variations in model parameters affect model outputs. It helps identify which parameters have the greatest impact, guiding model refinement and interpretation. Sensitivity measures can be local, assessing small perturbations around a nominal parameter set, or global, evaluating parameter influence over a wider range. In our study, we focus on local sensitivity analysis to capture the immediate response of the system to small changes in parameters.

This approach allows us to quantify the dependence of model predictions on specific parameters, helping to identify key contributors to system dynamics and potential sources of uncertainty. By systematically evaluating parameter influence, we ensure the robustness of our findings and gain a deeper understanding of the model’s behaviour.

We perform local sensitivity analysis using a sensitivity matrix 𝒮 to understand the effect of changes of individual parameters on the model output [50]. This analysis is performed numerically in R with the FME toolbox [51].

For a parameter $(\theta_{j}{)}_{j=1}^{m}$ the element of the matrix ${𝒮}_{ij}=\frac{\partial Y_{i}}{Y_{i}}\frac{\theta_{j}}{\partial \theta_{j}}$, where $Y_{i}=Y(t_{i}),Y\in \{A,U\}$ is the computed value at day $t_{i}=3.5+14(i-1),i=1,..\mathrm{.13}$. Cumulative measures of the local sensitivity of the parameter $\theta_{j}$ are norms such as $\frac{1}{13}\sum_{i}|{𝒮}_{ij}|,(\frac{1}{13}\sum_{i}|{𝒮}_{ij}{|}^{2}{)}^{1/2}$ as well as the mean $\frac{1}{13}\sum_{i=1}^{13}{𝒮}_{ij}$ and extremes $\min_{i}{𝒮}_{ij},\max_{i}{𝒮}_{ij}$. This produces a ranking of the importance of the changes of individual parameters on the outputs of interest.

## 3 Results

### 3.1 Local sensitivity analysis

**Fig 2:**
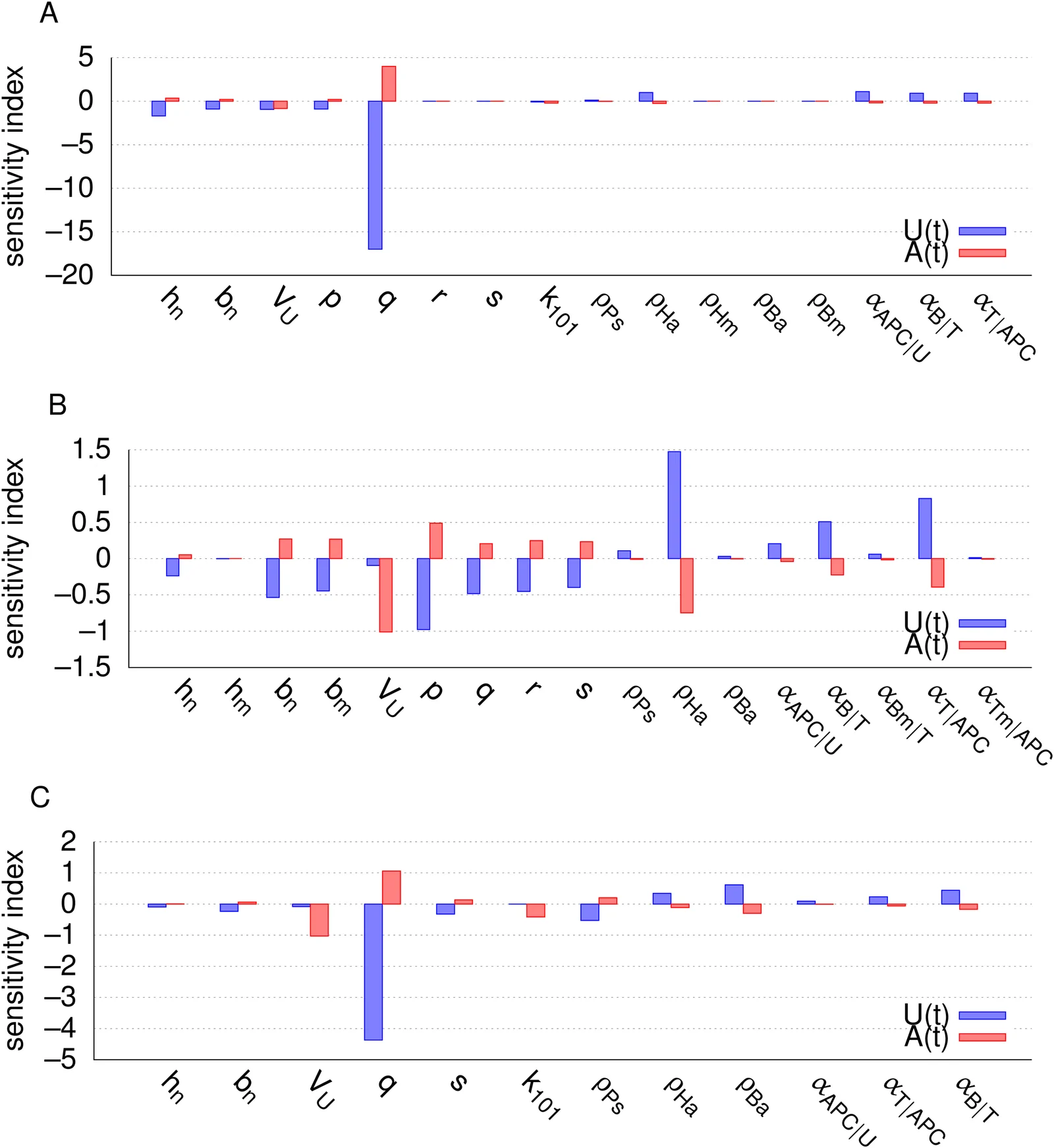
Local sensitivity analysis (sensitivity indices): the subcohort in ADA-low group with ADA < 30 AU/mL (A); the subcohort in ADA-low group with ADA between 30 and 100 AU/mL (B); the subcohort with transient ADA (C).

The ADA titres reported in the data set suggest a finer stratification of the ADA-low and ADA-high cohorts, and we reflect this structure in the sensitivity analysis and construction of virtual cohorts. For the ADA-low patient cohort we perform three separate sensitivity analyses: for the subcohort with transient immunogenicity (3 patients), for the subcohort with ADA titre below 30AU/mL (10 patients), and for the subcohort with ADA titre between 30 and 100 AU/mL (3 patients). The parameters with the highest sensitivity index (>0.5 in absolute value) with respect to the adalimumab concentration *U* and ADA titre *A* from the subcohort with ADA titre below 30AU/mL are $\alpha_{APC|U},\alpha_{T|APC},\alpha_{B|T},h_{n},b_{n},p,q,V_{U},\rho_{Ha}$, for the subcohort with ADA titres between 30 and 100 AU/mL the results are similar (Fig 2A-2B). For the transient ADA subcohort the model parameters with largest sensitivity indices are $V_{U},q,\rho_{Ps},\rho_{Ba},\rho_{Ha}$ (Fig 2C).

**Fig 3:**
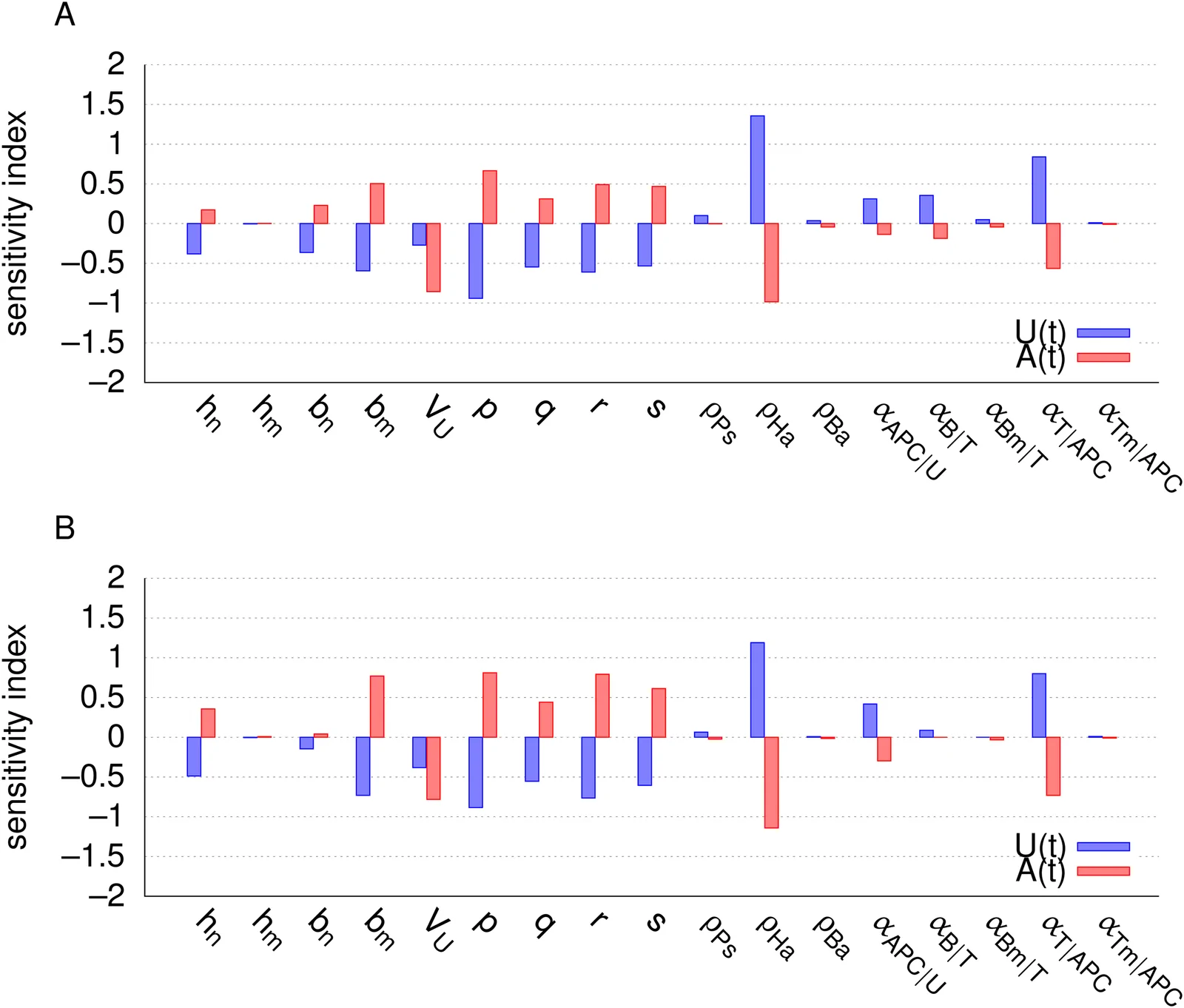
Local sensitivity analysis (sensitivity indices): the subcohort in ADA-high group with ADA < 1000 AU/mL (A); the subcohort in ADA-high group with ADA > 1000 AU/mL (B).

For the ADA-high patient cohort we perform two separate sensitivity analyses: for the subcohort with ADA titre below 1000AU/mL, and with ADA titre above 1000 AU/mL (observed in just 2 patients). For both ADA-high subcohorts the local sensitivity analysis shows the parameters with the highest sensitivity index (>0.5 in absolute value) with respect to the adalimumab concentration *U* and the ADA titre *A* are $\alpha_{T|APC}$, $b_{m}$, $V_{U}$, *p*,*q*,*r*,*s*, $\rho_{Ha}$ (Fig 3).

### 3.2 Virtual patient cohorts

Since the available patient data from CoMARIS do not contain counts of APCs, antigen-specific T or B lymphocytes, and many parameter values have unknown values or are non-identifiable from the data, we employ Latin hypercube sampling for the parameters exhibiting largest sensitivity indices (>0.5 in absolute value) to generate virtual patient cohorts corresponding to ADA-low (including transient ADA) and ADA-high cohorts. In order to represent the structure of the virtual patient cohorts according to the titre ranges for the ADA-low, ADA-high patients and those with transient immunogenicity, we vary the parameters with highest sensitivity indices in a Latin hypercube sample ($\alpha_{B|T},\alpha_{Bm|T},\alpha_{T|APC},\alpha_{Tm|APC},h_{n},b_{n},h_{m},b_{m},V_{U},\rho_{Ps}$). The reported ranges of their values in Section D in S1 Appendix reﬂect selective differentiation of the virtual patients driven by the data ranges of ADA titre in weeks 4, 8, 12 and 26 following the criteria for ADA-low and ADA-high patients [9], and the respective adalimumab concentration range. As the parameter $\rho_{Ps}$ has lower sensitivity in the ADA-high cohort, it is assumed to be fixed there. The parameter $\rho_{Ba}$ has a higher sensitivity in the transient ADA subcohort, so it is assumed to vary only there in order to produce virtual patients that conform to the ranges of the data. The probabilities *p*,*q*,*r*,*s* are assumed fixed. The range for the Latin hypercube for the parameters representing the putative therapeutic effects of MTX is chosen as mean value ± 15% (denoted henceforth as LH15), similar ranges are used to generate virtual cohorts in [52,53]. Furthermore, the point value of the PK parameter $V_{U}$ which is varied by ±15% agrees with the range of $V_{U}$ obtained by fitting individual patient data of adalimumab, for whom the fitted value $\delta_{U}$, in addition, agrees with the value reported elsewhere [54].

Pearson correlation coefficients for the parameter pairs with statistical significance at level 1% in the ADA-low, ADA-high and ADA-transient virtual patient cohorts are summarised in Table K in S1 Appendix. The observed correlations are generally moderate and do not systematically involve those parameters with the highest sensitivity indices (Figs 2 and 3). The high correlation between $h_{n},h_{m}$ may result from the assumption $h_{m}\leq h_{n}$ reflecting the different activation thresholds between naive and memory CD4^+^ T cells.

**Fig 4:**
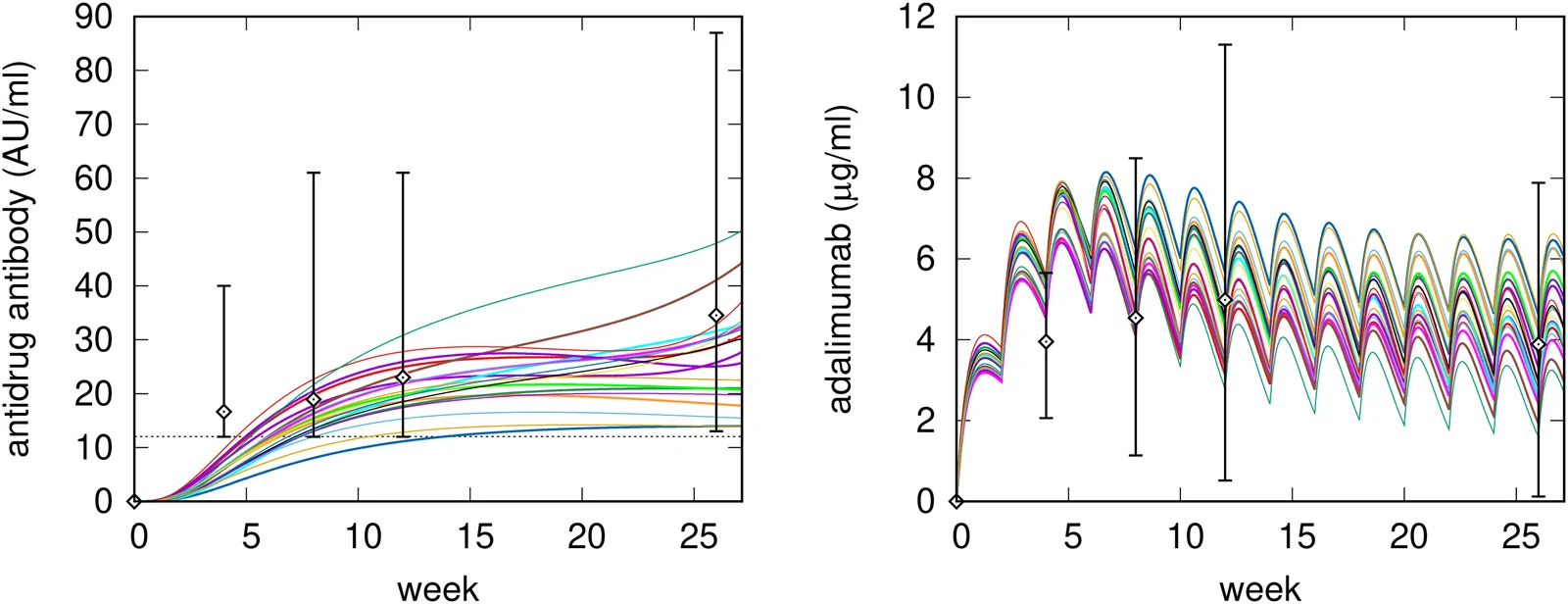
Selected MTX– virtual patients from ADA-low cohort. Error bars represent minimum and maximum (16 patients). Trajectories use parameter values listed in Section D in S1 Appendix. The LOD of the antibody assay (12 AU/mL) is indicated as a dotted line.

**Fig 5:**
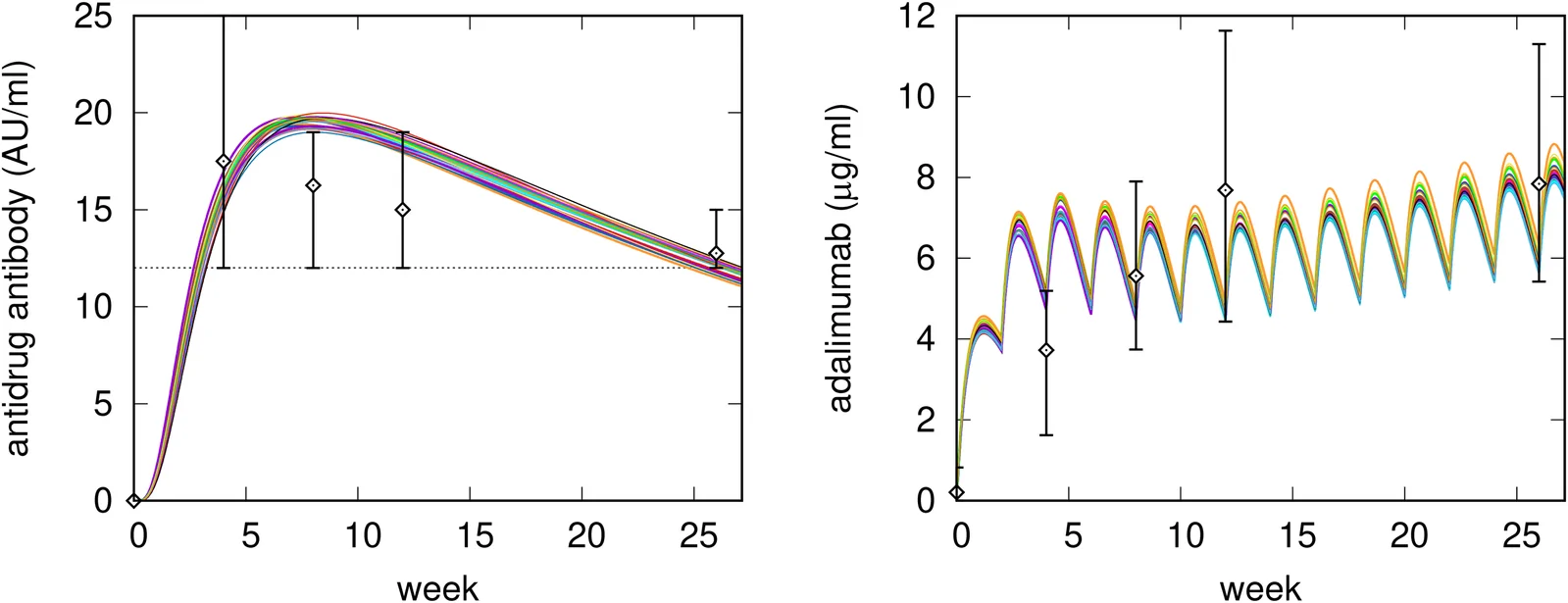
Selected MTX– patients from virtual cohort with transient ADA. Error bars represent minimum and maximum (3 patients). Trajectories use parameter values listed in Section D in S1 Appendix. The LOD of the antibody assay (12 AU/mL) is indicated as a dotted line.

**Fig 6:**
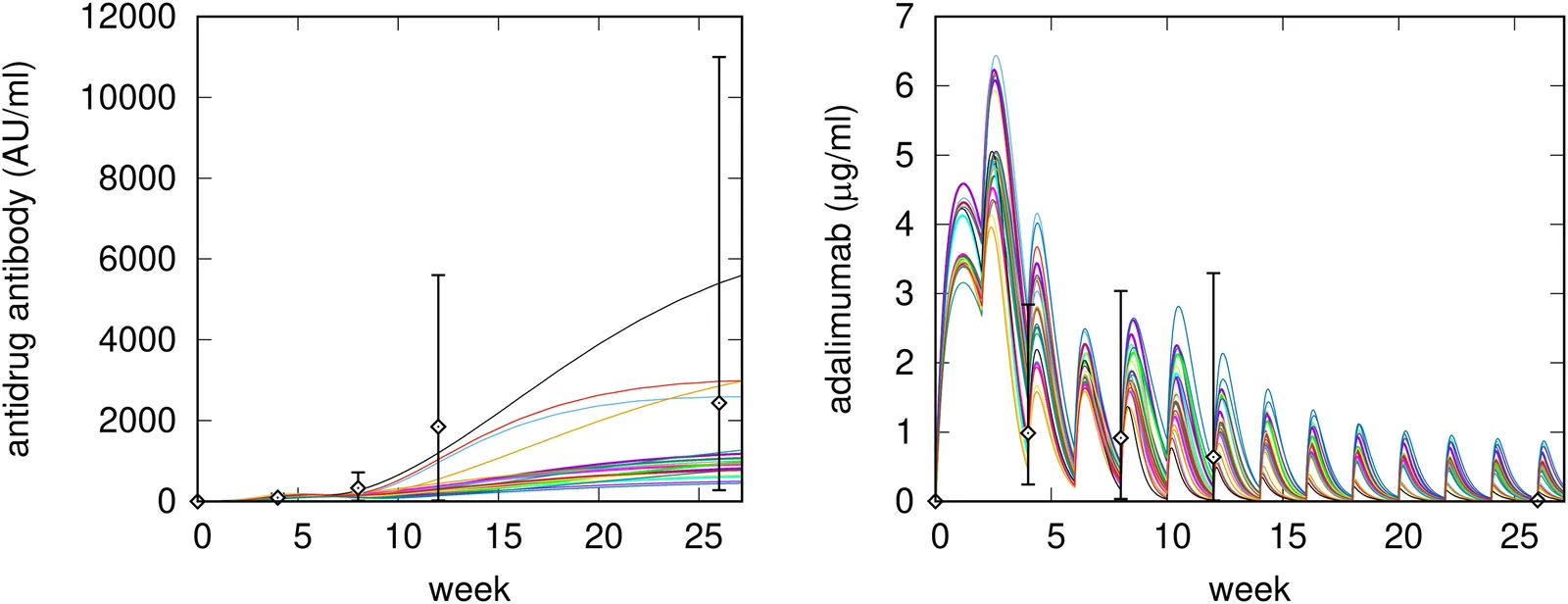
Selected MTX– patients from virtual ADA-high cohort. Error bars represent minimum and maximum (7 patients). Trajectories use parameter values listed in Section D in S1 Appendix. The LOD of the antibody assay (12 AU/mL) is indicated as a dotted line.

The selected parameter values allow us to explore the effect of parameter variability on the dynamics of the model by computing the solution for each virtual patient. Some solutions for the model without co-administration of MTX are plotted in Figs 4-6. We see that the dynamics for the low and ADA-high cohorts corresponds quite satisfactory to the patient data on adalimumab concentration and ADA titres. In the ADA-low cohort due to the nature of data reporting (ADA-negative values are reported as <12 AU/mL), the plotted values for the ADA titre use this cut-off value, so the minima are at 12AU/mL. Hence, the plotted data range for week 26 in Fig 5, left panel, appears to majorise the simulated trajectories. Simulations of ADA and adalimumab dynamics over a period of 52 weeks are provided in Fig Y in S1 Appendix and reveal that despite the different temporal profiles the classification by cohorts remains valid for the prolonged period.

To test for the effect of MTX on the immune response in these virtual patients we prepare a list of scenarios based on the admissible combinations of modes of action (Table 2). A check mark (✓) means that MTX is assumed to act on the specific cell population, and the terms describing MTX action corresponding to the particular scenario are included in the respective equation of the model. For example, in scenario 6 in Table 2, MTX is used only in the equations for APCs (9) and activated T and B lymphocytes (equations (11), (13), (17), (19)). Or, further, scenario 23 assumes that MTX acts only on APC apoptosis and Breg cell activation as described in equations (7) and (21), and omits the terms with MTX action in the other equations. In the respective scenario, we draw the parameter value in the functional responses for each hypothetical mechanism of action of MTX from a uniform distribution with ranges mean ±15% or with mean and SE derived from the auxiliary models listed in Section C in S1 Appendix. Then, using the virtual patient cohort, we perform simulations of all scenarios for the mechanism of MTX action. Illustrative results are shown in Figs 8-10 for scenario no. 28.

**Table 2:** List of model scenarios for the action of MTX on different cell types.

Scenario no.	APC	Activ. T	NK cells	Bregs	Activ. B
1	✓	✓	✓	✓	✓
2	✓	✓	✓		✓
3	✓	✓		✓	✓
4		✓	✓	✓	✓
5	✓		✓	✓	✓
6	✓	✓			✓
7	✓		✓		✓
8	✓			✓	✓
9		✓	✓		✓
10		✓		✓	✓
11			✓	✓	✓
12	✓				✓
13		✓			✓
14			✓		✓
15				✓	✓
16	✓	✓	✓	✓	
17	✓	✓	✓		
18	✓	✓		✓	
19		✓	✓	✓	
20	✓		✓	✓	
21	✓	✓			
22	✓		✓		
23	✓			✓	
24		✓	✓		
25		✓		✓	
26			✓	✓	
27	✓				
28		✓			
29			✓		
30				✓	
31					✓

The local sensitivity analysis gives us some indications of the scenarios that are most likely to affect the dynamics of ADA production. For example, the parameter $\alpha_{T|APC}$ that represents the T cell activation by APCs has a high sensitivity index in the ADA-low and ADA-high cohorts, and in some scenarios its value is affected by the value of *c*_10_, serving as a proxy for Breg production of anti-inflammatory cytokines such as IL-10 [11,29]. Similarly, the maximum proliferation rate of activated T cells $\rho_{Ha}$, another parameter with high sensitivity index is also affected by *c*_10_. Hence, we would guess that scenarios where MTX is taken to increase abundance of Breg cells (scenarios with no. 1, 3–5, 8, 10, etc. in Table 2) are more likely candidates. However, we stress that the model incorporates this particular mode of action in a phenomenological manner, as levels of IL-10 are not available in the CoMARIS data set, and the suppressive effect of IL-10 on APCs, T lymphocytes, etc. is hard to quantify against published data [28,35] due to the variety of assays used. In particular, the dynamics of activated T cells can be directly influenced by the MTX-induced apoptotic effect represented in other scenarios such as no. 2, 9, 13, 24, etc.

Using the parameter values drawn from the Latin hypercube LH15, we generate 190 virtual patients for the ADA-low cohort (30 of whom are transient ADA, 130 of whom have ADA titre below 30 AU/mL), and 100 for the ADA-high cohort (20 of whom have ADA titre above 1000 AU/mL). These cohort sizes are chosen as approximately 10-fold the cohort size reported in [9] and conform to the relative shares of the respective sub-cohorts according to ADA levels. We do not compute individual trajectories for 260 virtual patients for the ADA-negative cohort who do not receive MTX because those can be simulated by setting parameter values $\alpha_{B|APC}=\alpha_{T|APC}=0$ (no formation of Ag-specific T or B lymphocytes), $\alpha_{1}=0$ (no maturation of APCs) or $\alpha_{APC|U}=0$ (no loading and processing of antigen).

To simulate the different scenarios of the mechanism of action of MTX on the immune cell populations in the model, we perform simulations by substituting the values *X*(*t*) resulting from the MTX pharmacokinetic equation (4) with $D_{X}=10$ mg/mL into the equation(s) describing the dynamics of the respective cell population for each virtual patient as outlined in Table 2.

Then the resulting values of antidrug antibody titres at week 4, 8, 12 and 26 are compared against the criteria outlined in [9] and the MTX+ virtual patient is assigned one of 3 status categories: *ADA-positive MTX+ at week 26*, *ADA-high MTX +* , or *ADA-low MTX +* . The category *ADA-negative MTX +* is complementary to the last two, and not included in the estimation. To carry out this analysis we derive the conditional probabilities for a given patient transitioning between cohorts under MTX administration, denoted by ℙ(Y|ADA-low MTX--),ℙ(Y|ADA-high MTX--),ℙ(Y|ADA-neg MTX--), for each cohort $Y\in \{{\text{ADA-pos}}_{26}MTX+\},\text{ADA-high MTX+},\text{ADA-low MTX+}\}$.

Based on the parameter values that characterise the ADA-negative MTX– patients, we assume that for any virtual patient from the ADA-negative MTX– cohort, the probability to become ADA-negative under co-medication of MTX, ℙ(ADA-neg MTX+|ADA-neg MTX--)=1 in any scenario. For any scenarios with index i=1,...31 we compute the probability distribution for the 3 status categories


$$\begin{array}{cc} \mathbb{P}({\text{ADA-pos}}_{26}MTX+\},i) & =\mathbb{P}({\text{ADA-pos}}_{26}MTX+\}|\text{ADA-low MTX--})\mathbb{P}(\text{ADA-low MTX--})\\ & \;+\mathbb{P}({\text{ADA-pos}}_{26}MTX+\}|\text{ADA-high MTX--})\mathbb{P}(\text{ADA-high MTX--})\\ & \;+\mathbb{P}({\text{ADA-pos}}_{26}MTX+\}|\text{ADA-neg MTX--})\mathbb{P}(\text{ADA-neg MTX--}),\\ \mathbb{P}(\text{ADA-high MTX+},i) & =\mathbb{P}(\text{ADA-high MTX+}|\text{ADA-low MTX--})\mathbb{P}(\text{ADA-low MTX--})\\ & \;+\mathbb{P}(\text{ADA-high MTX+}|\text{ADA-high MTX--})\mathbb{P}(\text{ADA-high MTX--})\\ & \;+\mathbb{P}(\text{ADA-high MTX+}|\text{ADA-neg MTX--})\mathbb{P}(\text{ADA-neg MTX--}),\\ \mathbb{P}(\text{ADA-low MTX+},i) & =\mathbb{P}(\text{ADA-low MTX+}|\text{ADA-low MTX--})\mathbb{P}(\text{ADA-low MTX--})\\ & \;+\mathbb{P}(\text{ADA-low MTX+}|\text{ADA-high MTX--})\mathbb{P}(\text{ADA-high MTX--})\\ & \;+\mathbb{P}(\text{ADA-low MTX+}|\text{ADA-neg MTX--})\mathbb{P}(\text{ADA-neg MTX--})\;. \end{array}$$

Due to our assumption on the conditional probabilities that


$$\begin{array}{c} \mathbb{P}(\text{ADA-low MTX+}|\text{ADA-neg MTX--})=\mathbb{P}(\text{ADA-high MTX+}|\text{ADA-neg MTX--})\\ =\mathbb{P}({\text{ADA-pos}}_{26}MTX+\}|\text{ADA-neg MTX--})=0, \end{array}$$

the above expressions simplify.

**Fig 7:**
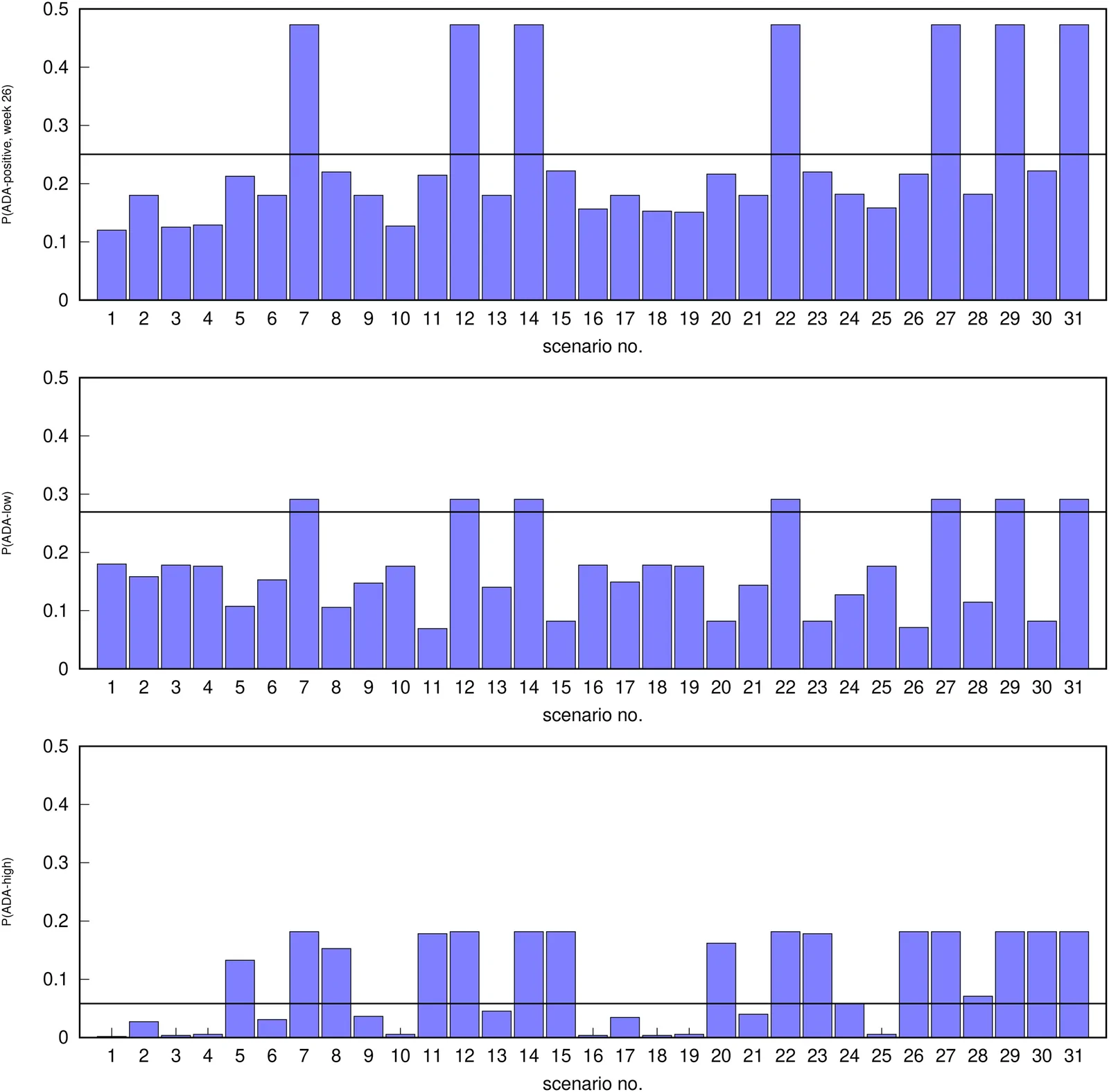
Computed probabilities for the Latin hypercube LH15 with methotrexate dose $D_{X}=10$ mg/mL for the 31 scenarios (horizontal axis).

The probabilities from the experimental observations in CoMARIS [9] are shown in Table 3, and compared against the probability distributions computed across all 31 scenarios in Fig 7. Histograms of the conditional probabilities resulting from the virtual patient cohorts drawn from the Latin hypercube LH15 are shown in Fig Z in S1 Appendix.

**Table 3:** Experimental observations of the probabilities $p_{i}$ based on [9]. *p*_1_ presents the probability of ADA presence at week 26 only, whereas *p*_4_ represents the probability of being ADA-negative across all visits.

Probability	Value
$p_{1}=\mathbb{P}({\text{ADA-pos}}_{26}MTX+\})$	0.25
$p_{2}=\mathbb{P}(\text{ADA-high MTX+})$	0.058
$p_{3}=\mathbb{P}(\text{ADA-low MTX+})$	0.269
$p_{4}=\mathbb{P}(\text{ADA-neg MTX+})$	0.673

Next, we define a metric to rank the hypothetical scenarios from most to least likely based on the model prediction. For scenario with no. i=1,...,31 we compute the squared residual deviation of the computed probabilities from the reported values $p_{j}$ (*j* = 1,2,3) (since $p_{2}+p_{3}+p_{4}=1$):


$$\begin{array}{cc} \Delta_{i} & =|p_{1}-\mathbb{P}(\text{ADA-pos MTX+},i){|}^{2}+|p_{2}-\mathbb{P}(\text{ADA-high MTX+},i){|}^{2}\\ & \;+|p_{3}-\mathbb{P}(\text{ADA-low MTX+},i){|}^{2}\;. \end{array}$$ (22)

To account for differences in model complexity across scenarios, we introduce an AIC-inspired model selection criterion. Unlike the AIC, our criterion is not derived from a likelihood function but is specifically designed to compare mechanistic scenarios by balancing the discrepancy with the experimental data against the number of active biological mechanisms. Consequently, standard interpretations of ΔAIC values and associated thresholds are not directly applicable to the proposed criterion; a detailed mathematical interpretation of the criterion, including its relation to the underlying discrepancy measure, is provided in Section A in S1 Appendix. Since the structure is fixed in the basic model without MTX, we use the number of active mechanisms across scenarios as a proxy for model complexity. The resulting metric is defined as


$$\begin{array}{c} {\text{IC}}_{i}=n\cdot \ln\left(\frac{\Delta_{i}}{n}\right)+2k_{i}\;, \end{array}$$ (23)

where *n* = 3 is the number of summary statistics and $k_{i}$ is the number of active mechanisms in scenario *i*. The scenario with index $i=\text{arg}\min_{i}{\text{IC}}_{i}$ is selected by the proposed criterion as the preferred mechanistic scenario within the present modelling framework.

The results in Table 4 show that scenario no. 28 achieves the lowest IC value. This scenario corresponds to a mechanism of MTX action inducing apoptosis of activated CD4 + T lymphocytes only. While several more complex scenarios (e.g., no. 13, 21, 25, 27, 29, and 31) yield comparable goodness of fit, their improved fit is marginal and does not compensate for the increased model complexity. In particular, scenarios no. 27 and no. 29 incorporate MTX effect respectively solely on APCs and NK cells, scenario no. 13 includes apoptosis of both activated B and T lymphocytes, and scenario no. 21 represents a combination of suppression of APC and activated T lymphocytes, while scenario no. 25 additionally incorporates activation of regulatory B cells. Overall, these results indicate that increased apoptosis of activated CD4 + T cells alone provides the most parsimonious explanation of the observed data within the current modelling framework, while additional mechanisms cannot be reliably identified given the available data. The resulting ADA profiles in the other scenarios do not match in a staisfactory manner the reported ADA ranges in MTX+ patients from CoMARIS data (Section E.2 in S1 Appendix).

**Table 4:** Ranking of scenarios according to the AIC-inspired criterion (23), with $D_{X}=10$ mg/mL. Lower IC values indicate a better trade-off between goodness of fit and model complexity.

Scenario	IC	Scenario	IC
**28**	**-11.951**	18	-8.934
25	-11.066	19	-8.863
21	-10.894	12	-8.524
13	-10.787	14	-8.524
27	-10.524	22	-8.524
29	-10.524	4	-8.249
31	-10.524	10	-8.199
24	-10.394	23	-8.133
30	-10.050	15	-8.050
2	-9.327	26	-7.875
6	-9.162	8	-7.064
17	-9.058	7	-6.524
16	-9.036	20	-6.421
9	-9.004	3	-6.163
18	-8.934	11	-5.915
19	-8.863	5	-5.453
		1	-4.025

**Fig 8:**
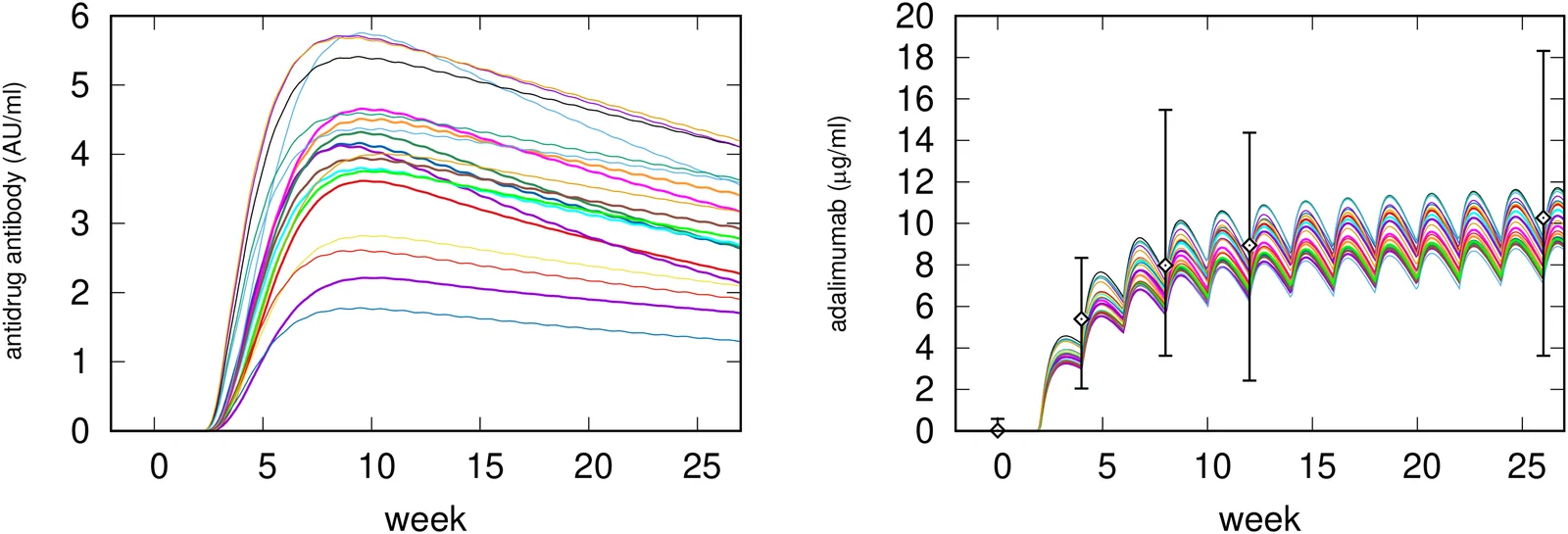
Selected virtual patients from the MTX + ADA-negative cohort under scenario no. 28 (MTX effect on activated T lymphocytes only). Error bars represent minimum and maximum of the CoMARIS data (35 patients). Trajectories use parameter values listed in Section D in S1 Appendix and drawn from LH15 with MTX concentration dose $D_{X}=10$ mg/mL.

**Fig 9:**
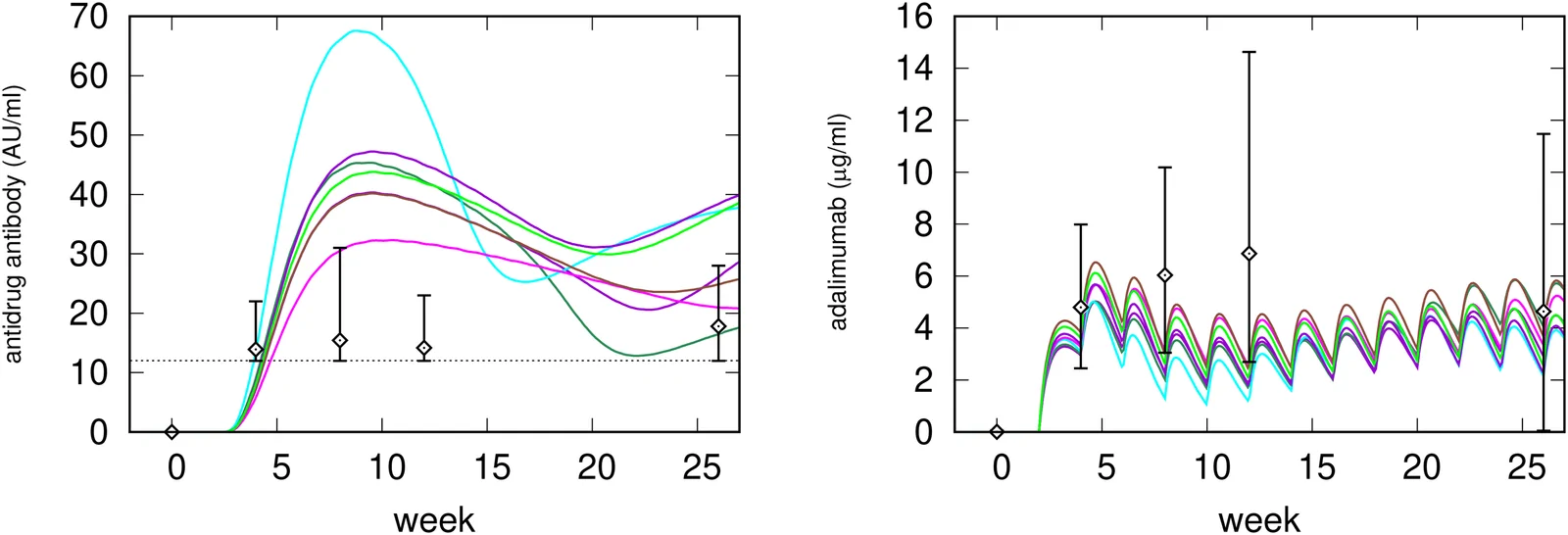
Selected virtual patients from the MTX + ADA-low cohort under scenario no. 28 (MTX effect on activated T lymphocytes only). Error bars represent minimum and maximum of the CoMARIS data (14 patients). Trajectories use parameter values listed in Section D in S1 Appendix and drawn from LH15 with MTX concentration dose $D_{X}=10$ mg/mL. The LOD of the antibody assay (12 AU/mL) is indicated as a dotted line.

**Fig 10:**
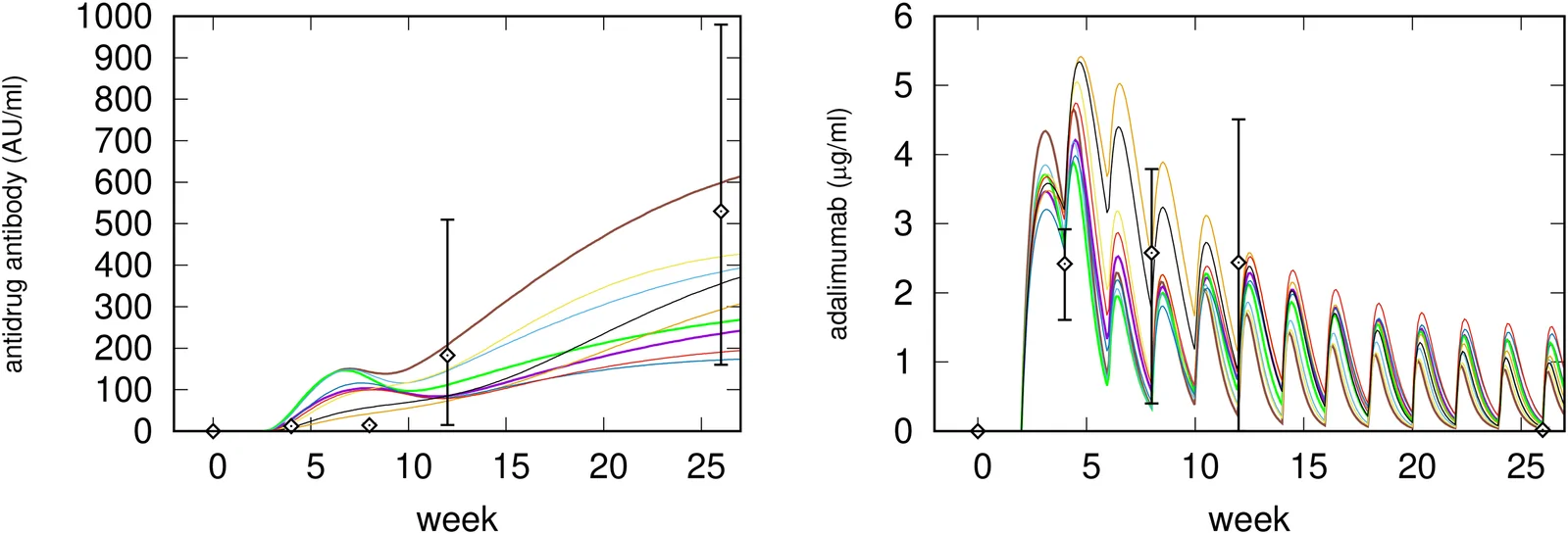
Selected virtual patients from the MTX + ADA-high cohort under scenario no. 28 (MTX effect on activated T lymphocytes only). Error bars represent minimum and maximum of the CoMARIS data (3 patients). Trajectories use parameter values listed in Section D in S1 Appendix and drawn from LH15 with MTX concentration dose $D_{X}=10$ mg/mL.

Illustrations of simulated adalimumab and ADA dynamics for selected virtual patients under scenario no. 28 are plotted in Fig 8 (ADA-negative), Fig 9 (ADA-low), and Fig 10 (ADA-high). The profiles of adalimumab and ADA for the entire virtual patient cohort under this scenario are shown in Fig I in S1 Appendix. Section E.2 in S1 Appendix containes the profiles for several scenarios with lowest IC (no. 13, 21, 25, 27 and 29) as illustration. Cell population dynamics for these four scenarios are illustrated in Sections E.5-E.9 in S1 Appendix.

### 3.3 Simulation of reactive co-administration of MTX

We employ the model to simulate a setting of reactive co-administration of MTX. That involves monitoring of adalimumab concentration and ADA titre for the patients and introduction of MTX if ADA are detected in the patient blood sample.

**Fig 11:**
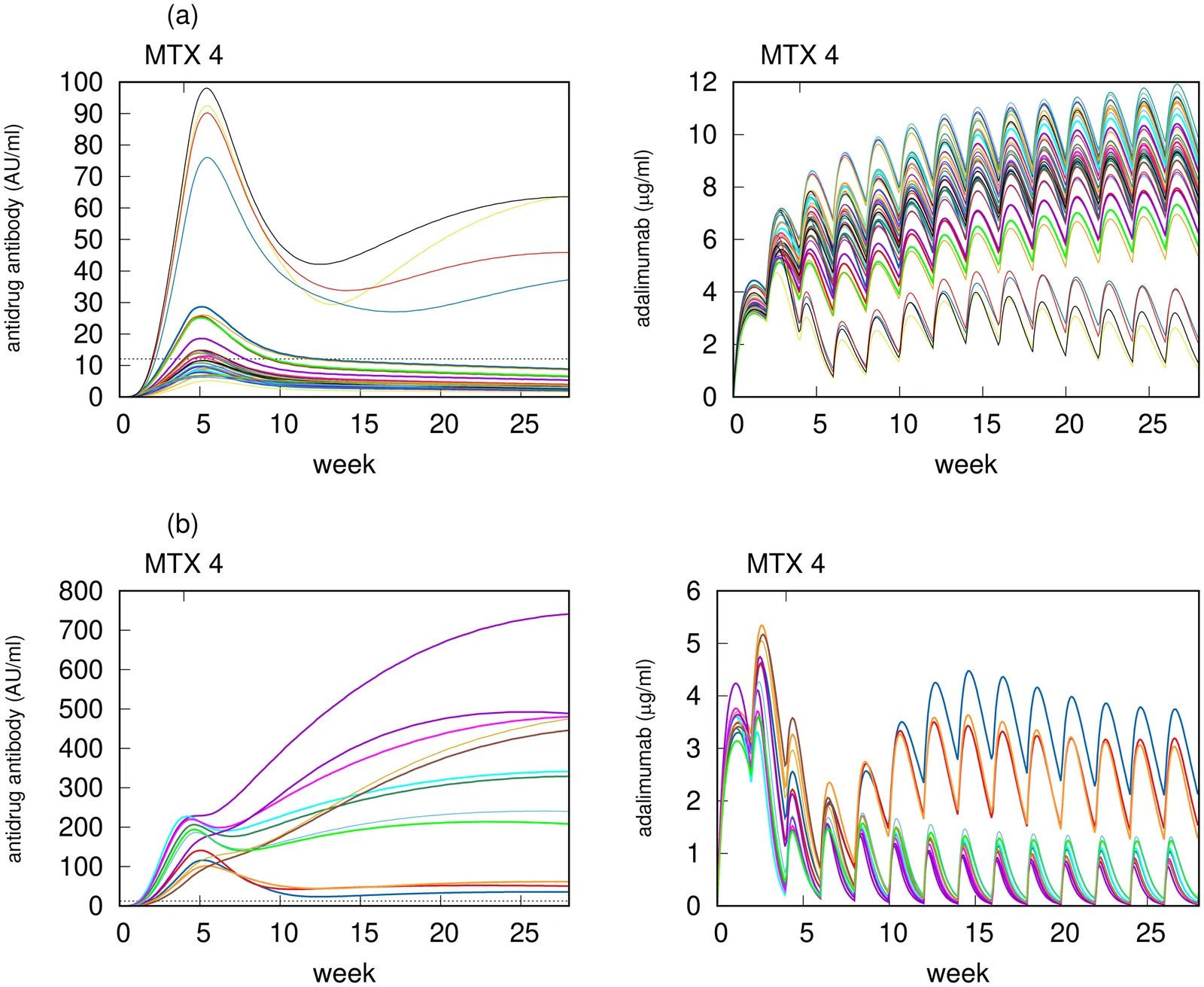
Simulation of the temporal dynamics for ADA titre and adalimumab concentrations for virtual patients from the ADA-low (A) and ADA-high (B) cohorts under scenario 28, with co-administration of MTX starting in week 4. The LOD of the antibody assay (12 AU/mL) is indicated as a dotted line.

We run the model under scenarios no. 28, 13, 24, and 29, which have the lowest values of the ${\text{IC}}_{i}$ (Table 4) and introduce a delay in MTX co-administration ($D_{X}=10$ mg/mL) in weeks 4, 8, or 12 (Figs 11, 13, and S-X in S1 Appendix), and simulate the dynamics over the following weeks.

Under scenario no. 28, which our analysis suggested as the most likely mechanism whereby MTX acts on immune cell populations, the results show that if MTX co-administration begins in week 4, some ADA-low virtual patients remain below the ADA titre threshold of 12 AU/mL, or have a rapid reduction in ADA titre following the MTX co-administration, whereby the adalimumab levels (Fig 11A) are similar to those in the CoMARIS MTX^+^ cohort [9]. Thus, on the one hand, for an ADA-low patient the decrease in adalimumab concentration is already latent and MTX maintains a concentration that tends to increase after 7 weeks. In the ADA-high virtual patient cohort, on the other hand, the group splits into two: some that maintain very high ADA titre (>200 AU/mL) and some that have a lower ADA titre, and adalimumab levels maintained very low or close to half of those in the ADA-low cohort respectively (Fig 11B). Thus, for some ADA-high patients delayed co-administration of MTX results in a partial restoration of adalimumab levelss, but for others, there is no significant impact on adalimumab concentration compared to the case when MTX is administered from week −2, before the TNF inhibitor therapy begins.

**Fig 12:**
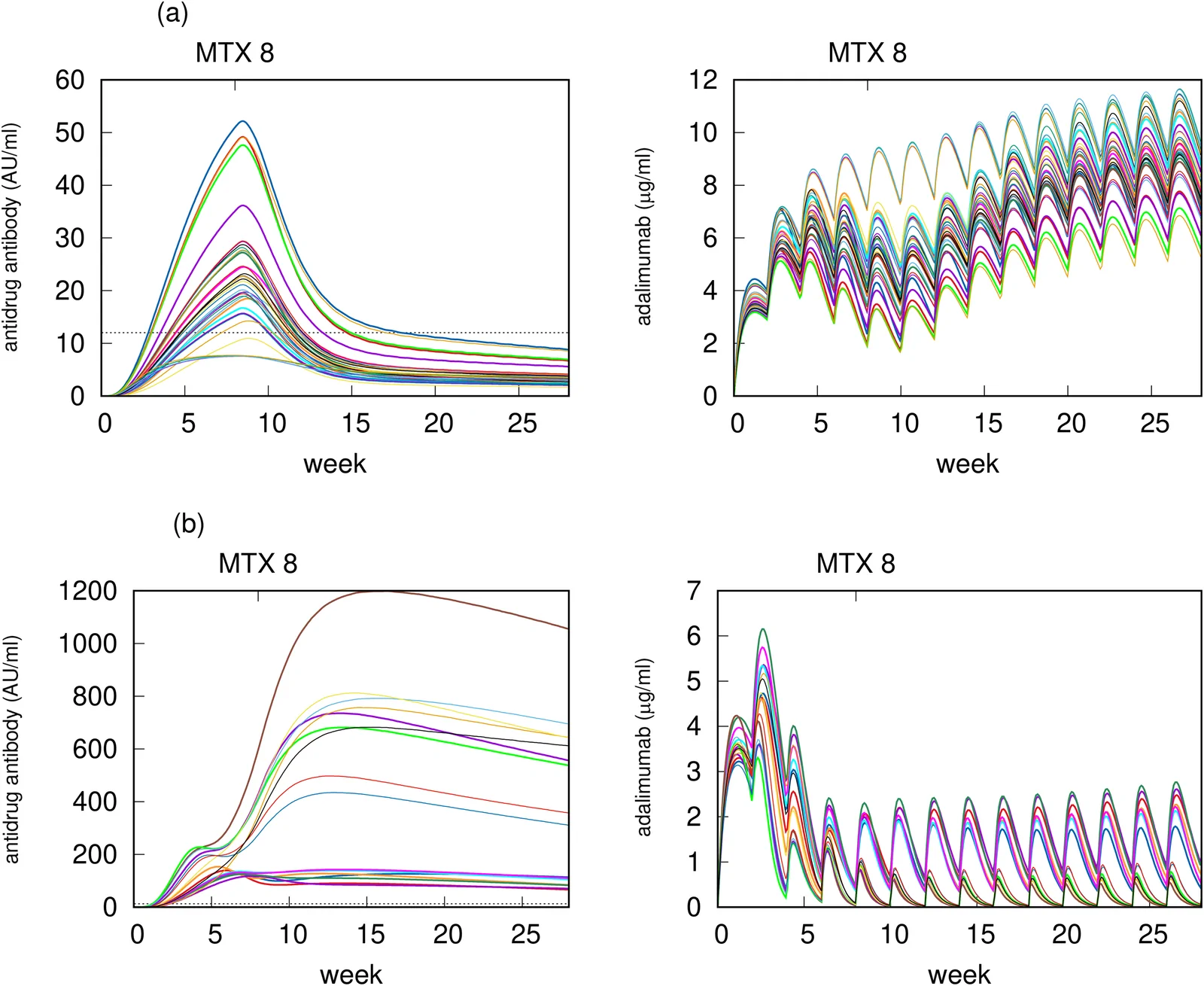
Simulation of the temporal dynamics for ADA titre and adalimumab concentrations for virtual patients from the ADA-low (A) and ADA-high (B) cohorts under scenario 28, with co-administration of MTX starting in week 8. The LOD of the antibody assay (12 AU/mL) is indicated as a dotted line.

**Fig 13:**
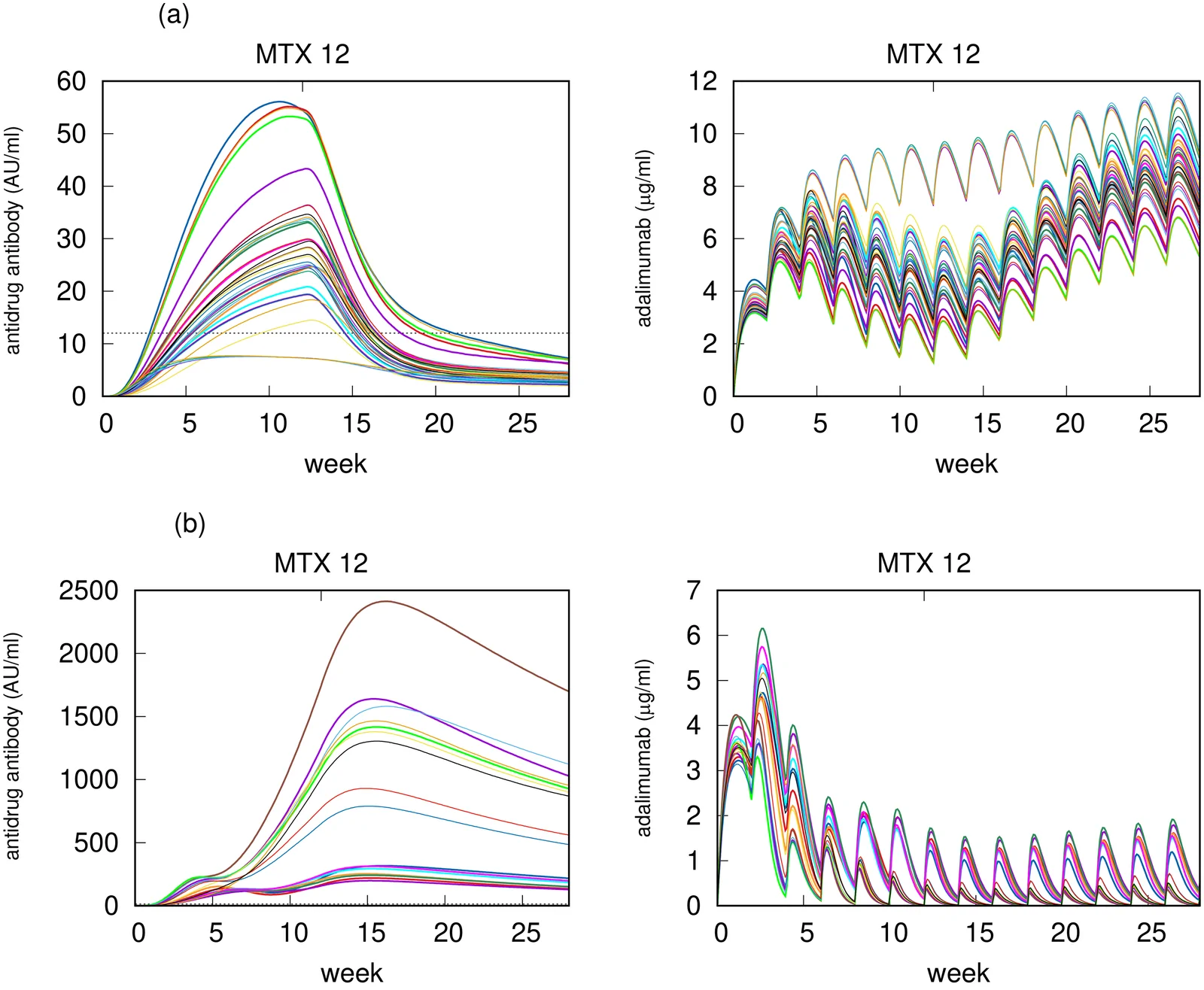
Simulation of the temporal dynamics for ADA titre and adalimumab concentrations for virtual patients from the ADA-low (A) and ADA-high (B) cohorts under scenario 28, with co-administration of MTX starting in week 12. The LOD of the antibody assay (12 AU/mL) is indicated as a dotted line.

On the contrary, if MTX co-administration begins in weeks 8 or 12, there is a decrease in the concentration of adalimumab compared to the case where MTX is introduced from week −2 in the ADA-high cohort. If the patient is classified as ADA-low, then the dynamics resembles more closely the one that observed in the CoMARIS data set, and the levels of adalimumab is eventually restored towards week 20 and beyond if MTX co-administration starts in week 8 or 12 (Figs 12A and 13A). However, if the patient is classified as ADA-high and has ADA titre > 1000 AU/mL at week 12, then MTX co-administration does not bring any restoration of adalimumab levels, which remain below 2–3 μg/mL (Figs 12B and 13B). This difference may be explained by the time lag that is required for the adaptive immune response (emergence of adalimumab-specific T and B lymphocytes and plasma cells). If the MTX co-administration begins after the start of the adaptive immune response, for the ADA-high patients it would cause no effect in counteracting immunogenicity. Results for delayed MTX co-administration in the scenarios no. 13, 24 and 29 are similar and shown in Section E.8 in S1 Appendix.

## 4 Discussion

We develop and simulate a mathematical model that describes the mechanism of the immune response to human monoclonal antibody adalimumab in treatment of axial spondyloarthritis patients and the changes on immune cell responses that arise under methotrexate co-administration based on CoMARIS patient data [9]. The model adapts ideas for the mechanisms of interaction between different immune cell populations based on previous models of the immune response [14,15]. Simulations of the model are performed to assess the potential impact of co-administration of methotrexate in reducing the immunogenicity of adalimumab in AxSpA by considering different scenarios of MTX acting on different immune cells (antigen-presenting cells (dendritic cells and macrophages), natural killer (NK) cells, B regulatory cells, activated T or B cells, or combinations thereof). The data used to calibrate the model have been sourced from the CoMARIS patient study [9] as well as from *in vitro* studies on MTX activity on different types of lymphocytes [27,36] and motivated by experimental observations [10,26,48]. The model is able to capture the different categories of anti-drug antibody response (ADA-negative, ADA-low, ADA-high, transient immunogenicity) as set out by the criteria presented in [9]. Sensitivity analysis has been used to select those parameters that exert the largest effect on the temporal dynamics of adalimumab and antidrug antibodies.

The system of ODEs has been numerically integrated to generate the immune response for virtual patient cohorts classified as ADA-low, ADA-high or transient immunogenicity (transient ADA) – 550 virtual patients in total – whose frequency is in accordance with their respective observed frequency within the MTX– group of patients in the CoMARIS study [9]. Then simulations of the immune response dynamics have been performed for the patient cohorts across all 31 scenarios for potential mechanisms of MTX action. The resulting MTX+ patients have been classified again as ADA-low, ADA-high or ADA-positive at week 26. The conditional probabilities of switching between categories under MTX co-medication have been computed, revealing the frequencies of MTX+ virtual patients who are ADA-low, ADA-high or ADA-positive at week 26, and these have been compared against the reported values in CoMARIS [9]. A residual deviation between the simulated and observed frequencies has been employed as a metric to rank the possible scenarios.

As expected from the sensitivity analysis, the model simulations predict that the most likely candidates for MTX action on reducing immunogenicity are combinations of: MTX-mediated apoptosis of activated naive and memory B and T cell populations. In particular, scenario no. 28 in Table 4 is the one that produces a best fit to the patient distribution surveyed in [9] with minimal complexity. Furthermore, the importance of low-dose MTX in induction of immune tolerance by action of Breg cells (second best scenario no. 25) is also observed in an experimental setting [48] independent of our dataset and model. The third best fit (scenario no. 21) includes in addition MTX effect on APCs, while in the fourth bestr scenario no. 13, MTX induces apoptosis of activated B and T lymphocytes as observed in [12,36], although the later study is done with lymphocytes from healthy donors. In scenario no. 24, MTX increases apoptosis of activated naive and memory T cells as well as suppresses mature cytotoxic NK cells, and in scenario no. 9 MTX-induced apoptosis of activated B and memory B cells is also included.

It may seem unexpected that NK cells do not participate in any of the 4 most likely scenarios of MTX action in the context of our mathematical model even though the data set revelas significant changes in their abundance for the MTX+ cohort. That may be due to the fact that TNF-α levels are barely detected at baseline, and the model is not in a position to capture links between MTX and its putative effect on NK cells in modulating the adaptive immune response. Reports in the literature on the role of Bregs in suppressing immunogenicity via IL-10 [11,48] have prompted us to include their effect on other immune cells in a rather rough mechanistic manner because measurements of IL-10 are not available in the CoMARIS data set. Such scenarios do not appear among the most likely candidates in the context of our mathematical model, in fact, scenario no. 30 whereby the MTX action is restricted to Bregs ranks only ninth in Table 4. Potential cross-effects via the mechanism of B cell activating factor BLyS/BAFF as reported by [11] have also been omitted due to inconclusive evidence from the CoMARIS data set on BAFF concentrations (Fig D in S1 Appendix), probably due to small sample size.

Looking at the frequency of the three status categories in the virtual patient cohorts across all 31 scenarios, in most scenarios the frequency of ADA-low patients and ADA-positive at week 26 patients is lower than observed by [9]. We note that many patients in the ADA-low cohort have ADA titres slightly above the assay threshold of 12 AU/mL, so in fact, those could be considered as ADA-negative instead, and the frequencies of patients who are ADA-low or ADA-positive at week 26 would decrease. Furthermore, this discrepancy could be due to the small number of patients (for instance only 3 CoMARIS patients in the MTX+ cohort were reported as ADA-high).

We compare the cellular dynamics between the virtual MTX– ADA-low and ADA-high patient cohorts (Figs K and L in S1 Appendix). The dynamics of mature APC in the MTX– ADA-low is such that these cells reach a plateau while in ADA-high cohort the dynamics is biphasic – growth during the initial weeks followed by a decrease, due to the suppressed concentration of adalimumab. The activated naive T, T memory and effector T cells curves are similar in shape for the ADA-low and ADA-high cohort (biphasic trend) because of the corresponding APC dynamics, on which they closely depend.

Importantly, the CD4 + CD25 + population measured in the CoMARIS study likely includes both activated effector T cells and regulatory T cells (Tregs), as previously reported in rheumatoid arthritis and other inflammatory settings [55]. Therefore, a reduction in activated CD4 + T cells induced by MTX may not necessarily translate into a measurable decrease in the total CD4 + CD25 + compartment if other CD25-expressing subsets remain stable. This may explain why the simulations predict a substantial reduction in activated T cells whereas the experimental CD4 + CD25 + counts do not significantly differ between MTX– and MTX+ patients.

The counts of activated APC are lower in the ADA-high cohort compared to the ADA-low cohort (Fig L). The activated naive B lymphocytes and activated memory B lymphocytes are significantly higher in the ADA-high cohort compared to the ADA-low cohort, as well as both short- and long-lived plasma cells. The transient non-monotone dynamics for the memory B cells observed around week 10 for the ADA-low MTX– cohort and week 4 for the ADA-high MTX– cohort is due to the start of their T-cell dependent activation and differentiation into long-lived plasma cells. In Fig K we observe low abundance of B memory and long-lived plasma cells, which we interpret as indicators of very weak long-term immunity against adalimumab, manifested via low ADA titres. Unfortunately, counts of the long-lived plasma cells are absent from the data, and due to the deterministic nature of the model we are not able to prove or disprove their extinction which may result from stochastic effects in a small population.

Next, we compare the cellular dynamics between the virtual MTX– and MTX + ADA-low and ADA-high patient cohorts under scenario of MTX action no. 28 (Fig M in S1 Appendix). The MTX + ADA-low virtual patient cohort has lower counts of T memory, activated T memory and effector T cells than the MTX– ADA-low cohort, but slightly higher levels of the activated naive B cells. MTX + ADA-low patients have comparable counts of activated B memory cells, and short-lived plasma cells and lower counts of long-lived plasma cells.

The MTX + ADA-high virtual patient cohort (Fig M) has comparable counts of mature APCs, which may be due to the lower concentration of circulating adalimumab in both cases. The activated T cells and the T effector cells are significantly reduced (4–5-fold at week 26). We note that the model predicted counts for activated T cells are lower than the counts of CD4 + CD25 + T cells from the CoMARIS dataset. In fact, CD4 + CD25 + T cell counts are already positive in both MTX– and MTX+ patient cohorts two weeks prior to the first adalimumab infusion (Fig A in S1 Appendix). Furthermore, according to [36] cell surface expression of CD69, CD25 and CD95 that are typical markers of activated T cells in the G1 phase of the cell cycle is not decreased by MTX. The observed difference likely reflects the model’s representation of T cell activation and MTX effects rather than a direct discrepancy with the underlying biology.

Activated naive B cells are slightly lower than those in the MTX– ADA-high cohort. B memory are reduced and especially activated B memory cells are 4–5-fold lower compared to the MTX– ADA-high cohort. Both short-lived and long-lived plasma cells are decreased. However, some virtual patients exhibit very high counts of activated B memory cells, leading to high counts of long-lived plasma cells, and persistent immunogenicity and elevated production of ADA of >200 AU/mL by week 12 (Figs 10 and M). The simulated dynamics for the immune cells are similar for the remaining scenarios (plots shown in Sections E.5-E.9 in S1 Appendix). A prospective study covering a wider range of cell types would be helpful tool in validating the model.

We also make model-based predictions on the adalimumab levels and ADA titre dynamics under a delayed co-administration of MTX, according to a reactive protocol when therapeutic monitoring of ADA is undertaken. We simulate four scenarios corresponding to the modes of MTX action on the immune cell populations (scenarios no. 28, 13, 21 and 25) combined with delayed co-administration starting at week 4, 8 and 12 compared to MTX being introduced 2 weeks prior to the biological therapy as described in CoMARIS [9] using the virtual patient cohorts. In the case of virtual patients from the ADA-low cohort the model does not predict significant differences in the outcome between the different delays of MTX introduction. The computational results show that the earlier the co-administration of MTX starts, the better is the outcome for some patients in the virtual ADA-high cohort. This may be explained by the time lag in the emergence of adaptive immune response, which may be counterbalanced by early MTX co-administration. To validate the findings of our mathematical model and the predictions based on reactive protocols, we suggest a prospective patient study as future line of research. We also observe from our model that immunogenicity to adalimumab is never fully suppressed, which is corroborated by studies using this TNF-α inhibitor [5,9,13].

Next, we discuss our model’s limitations deriving from the assumptions and the availability of data. While overproduction or inappropriate production of TNF-α (produced largely by macrophages in response to inflammatory stimuli such as lipopolysaccharide) may underlie various chronic inflammatory diseases [56], the amount of TNF-α for the majority of patients in the data set remains below baseline and cells such as macrophages are not measured. A major point is the patient samples lack measurements of antigen-presenting cells and antibody-secreting plasma cells, for which we have used estimates from the literature when available. This may be a result from the fact that dendritic cells and macrophages normally reside in tissue, and not in the serum which is used for the lymphocyte analysis.

Furthermore, T and B lymphocytes are presented as total counts according to CD markers in the flow cytometry, and not examined for antigen-specificity, making it difficult to estimate the extent of adalimumab-specific immune cell response. Moreover, several ADA measurements are affected by the assay detection limits, further reducing the quantitative information available for model calibration and limiting the achievable agreement between simulations and experimental observations. The model does not include regulatory T cells because of several reasons: functional defects related to the AxSpA pathology [57], and limited biochemistry data on cytokine levels in the CoMARIS dataset that prevents robust formulation of assumptions on the regulatory T cell response in immunogenicity and ADA production.

Potential differences in MTX pharmacokinetics between patients are not addressed as no temporal data on concentration of MTX nor of its metabolites (MTX-polyglutamate) are available in the CoMARIS data set. Again this can be a product of technical limitations since MTX has a relatively rapid clearance from the organism [58,59]. To overcome this gap in the data, we use the estimated value for the dilution coefficient of adalimumab $V_{U}$ from the adalimumab data to infer the value for the dilution coefficient of MTX $V_{X}$ and to simulate the temporal dynamics of MTX. This modelling approach, nevertheless, may be masking the true pharmacokinetics of MTX or its metabolites which seem to have a slower clearance and prolonged effects once absorbed by the cells [10].

To parametrise the effects of MTX, we have relied on *in vitro* studies based on different protocols, to parametrise simplified models. This approach may cause under- or overestimation of MTX effects, and lead to additive, and not synergistic effects in the analysis of the different scenarios for MTX mode of action. Such issues should be addressed by more detailed analysis of immune cell subpopulations.

Last, we raise attention to the paucity of reported biomarkers in AxSpA which are relevant to the immune cell kinetics. Most patient studies report values of CRP, VEGF, MMP-3 or IL-6 concentrations at one or two time points only, erythrocyte sedimentation rate, or HLA-B27 status [60,61]. In the CoMARIS dataset few TNF-α measurements were detected at baseline and most of them could not be quantified, so they are not used in the model. No IL-10 was measured, which could be used to fine-tune the modelled mechanism of suppression of different immune cell populations. For that reason, we have modelled IL-10 effects indirectly and primarily via the B10 precursor, CD24^high^CD27 + cells. In particular, the model’s predictive power could be expanded with a broader data set of biomarkers and cell types, as well as increased frequency of time samples.

Our model corroborates experimental findings that MTX modulates the apoptosis of activated T lymphocytes (as represented by scenario no. 28). Even though a mathematical model can serve as best as a complementary tool in any biological experiment, our results may be useful as guidelines for design of a future study performing a targeted analysis of these immune cell populations and validating *in vitro* or *in vivo* the respective mechanisms of action whereby MTX reduces immunogenicity towards the monoclonal antibody adalimumab. A long-term goal would be to derive criteria for selecting the right therapeutic dose in patient-tailored, precision dosing. However, this should be based on a prospective study that includes an expanded longitudinal set of cellular data, biochemistry, and biomarkers, as well as a potentially expanded set of selection criteria for the different patient cohorts.

## Supporting information

S1 Appendix Additional information and figures. Contains summary of auxiliary models, parameter values and numerical simulations.
(PDF)
