# Supplementary material for: Methotrexate’s effect on cells and adalimumab immunogenicity in axial spondyloarthritis: A mathematical study
Source: PLoS Comput Biol. 2026 Sep 21;22(9):e1014740. doi: 10.1371/journal.pcbi.1014740 (PMC13592724; doi:10.1371/journal.pcbi.1014740)
Supplement: Supplementary file 1 [file pcbi.1014740.s001.pdf]

Additional information supplementing the manuscript 'Methotrexate's Effect on Cells and Adalimumab Immunogenicity in Axial Spondyloarthritis: A Mathematical Study'

S. Sottile, C. Paul, R. Audo, T. Rispens, D. Mulleman, P. Rashkov

Contents

A Parameter values and scenario selection **2**

B Analytical solution for PK models **5**

C Parameter estimation from auxiliary low-dimensional models **7**

C.1 ADA-adalimumab system . . . 7
C.2 Estimation of the PK parameters for adalimumab V_U, δ_U . . . 8
C.3 Estimation of the MTX dilution coefficient V_X . . . 8
C.4 Estimation of the MTX-induced apoptotic effect on activated T lymphocytes . . . 9
C.5 Estimation of the MTX-induced apoptotic effect on activated B lymphocytes . . . 10
C.6 Estimation of the effect of MTX on APC differentiation . . . 10
C.7 Estimation of the effect of MTX on regulatory B cell activation . . . 11
C.8 Estimation of the suppressive effect of MTX on NK cells . . . 11

D Model Parameters **13**

E Supplementary Figures **18**

E.1 Cell counts . . . 18
E.2 Model-predicted adalimumab and ADA dynamics under MTX co-administration . . . 22
E.3 Model-predicted cell dynamics . . . 25
E.4 MTX+ virtual patient cohort under scenario no. 28 . . . 27
E.5 MTX+ virtual patient cohort under scenario no. 13 . . . 28
E.6 MTX+ virtual patient cohort under scenario no. 21 . . . 29
E.7 MTX+ virtual patient cohort under scenario no. 25 . . . 30
E.8 MTX+ virtual patient cohort under scenario no. 27 . . . 31
E.9 MTX+ virtual patient cohort under scenario no. 29 . . . 32
E.10 Simulations for delayed introduction of MTX . . . 33

E.11 Simulations of MTX- patients over a period of 52 weeks 39
E.12 Conditional probabilities 40

A Parameter values and scenario selection

Total cell counts. Concentration of dendritic cells (DCs) in healthy subjects is 13000-37000 cells/mL [15], not significantly different in patients with spondyloarthritis as reported by [40]. In patients with rheumatoid arthritis, numbers reported are 6000-45000 cells/mL [9].

Reported concentration of macrophages in healthy subjects is 200000 cells/mL [23], but due to the low-levels of IFNγ in synovial fluid reported in patient samples [38], we consider a reduced contribution of activated macrophages to antigen presentation. Thus we set a carrying capacity for APCs to be $K_D = 40000$ cells/mL to reflect that assumption.

We perform statistical hypothesis testing on the CoMARIS dataset to determine whether MTX alters the dynamics of different lymphocyte subpopulations. We denote by $\bar{\mu}_i$ the sample mean of the respective lymphocyte count in week $i = 0, 4, 12, 26$. We employ a paired t-test to test the null hypothesis $\mathrm{H}_0 : \bar{\mu}_{-2} \geq \bar{\mu}_i$ or $\mathrm{H}_0 : \bar{\mu}_{-2} \leq \bar{\mu}_i$ in order to infer whether MTX affects the abundance of the respective lymphocyte subpopulation.

There is no statistically significant difference in the counts of naive $CD4^+CD25^-$ T cells, nor in $CD4^+CD25^+$ T cells between the visits (Fig A). We assume that a small fraction of naive T cells is available for antigen presentation at the beginning of the therapy. Hence, the initial value of the $CD4^+CD25^-$ T lymphocyte $\bar{H}_0$ is chosen less than 10% of the mean value of the $CD4^+CD25^-$ cells across both patients group over all visits shown in Fig A.

A similar assumption is made for the initial concentration of naive B cells ($CD19^+CD27^-$) $\bar{B}_0$, which is chosen to be less than 5000 cells/mL.

Furthermore, the data reveal that co-administration of MTX brings about a significant reduction in NK cell and mature, cytotoxic NK cell counts (Fig B), suggesting a potential MTX-mediated suppressive effect as observed in mouse models or in human patients with rheumatoid or psoriatic arthritis [19, 29, 35]. Reported total levels of NK cells in another patient study [2] are between 50000-240000 cells/mL. We take the value of the carrying capacity K_{NK} to be close to the mean value at week -2 in each cohort of the $CD3^-CD56^+$ cells.

Finally, MTX co-administration brings a significant increase in $CD19^+CD27^+$ B memory cells, and $CD24^{hi}CD27^+$ cells but not in $CD24^+CD38^{hi}$ cells. Those last two subsets can differentiate into IL-10 producing B regulatory cells (Fig C) [7].

Antibody production. The value for the antibody production rate p_A was approximated based on data reported in [17]. Using the conversion of 10^7 molecules to 0.166×10^{-16} mol and molar weight of IgG (150000 g/mol) and IgM (900000 g/mol), we assume p_A as average value between the average rates of production of antibody in the ranges $(2.989 \times 10^{-4}, 5.978 \times 10^{-4}$μg/(cell day) for IgG and $(1.793 \times 10^{-3}, 3.58 \times 10^{-3})$μg/(cell day) for IgM. Although for most simulations we use the value $p_A = 2.69 \times 10^{-3}$ μg/(cell day), occasionally a higher value may be used 4×10^{-3} μg/(cell day) for the ADA-high

cohort.

Transient immunogenicity. To model the instances of transient immunogenicity observed for three patients in the ADA-low cohort of CoMARIS, we set the model parameters characterising the probability of differentiation to memory B and T cells $p = r = 0$, the activation of memory T cells $\alpha_{\mathrm{Tm|APC}} = 0$, the activation of memory B cells $\alpha_{\mathrm{B_m|T}} = 0$, and the maximum proliferation rates of memory B and T lymphocytes $\rho_{Bm} = \rho_{Hm} = 0$. Thus, we ensure that no immune memory cells form, in other words,

$$B_m(t) = B_{ma}(t) = H_m(t) = H_{ma}(t) = 0$$

for all $t \geq 0$.

In addition, we suppose that in this case short-lived plasma cells play a central role in the ADA production, and so assume ρ_{Ps} to be significantly higher than in the other cases. These assumptions correspond to the potential mechanism of immune tolerance as suggested in [1] namely, the absence of co-stimulatory pro-inflammatory signals prevents the activation of antigen-specific B cells by antigen-specific effector T cells, and the differentiation of antigen-specific B cells to plasma cells is suppressed.

Calibration of parameter values. In order to calibrate the values for the maximum activation rates of T and B lymphocytes $\alpha_{\mathrm{T|APC}}, \alpha_{\mathrm{T_m|APC}}, \alpha_{\mathrm{B_m|T}}, \alpha_{\mathrm{B|T}}$, and the weights for the activation thresholds h_n, h_m, b_n, b_m, we perform a parameter search such that the resulting maximum values of H_a in weeks 4, 12, 26 do not exceed 50% of the reported values of $CD4^+CD25^+$ T cells (Fig A), and the maximum of $B_m + B_{ma}$ in weeks 4, 12, 26 do not exceed the reported values of $CD27^+$ memory B cells in the CoMARIS study (Fig C).

The rate for loading of APCs with antigen $\alpha_{\mathrm{APC|U}}$ is taken to be similar to the value taken in [14] for the transient ADA case, and 2.5-fold for the ADA-low and ADA-high cases to achieve comparable temporal dynamics for the maturation of APCs.

The carrying capacities for the memory T and B cells K_{Hm}, K_{Bm} are assumed to be 100 cells/mL, with the carrying capacity K_{Bm} for ADA-low cohort given values 10, 20 or 100 in the parameter fit.

Selection of virtual patients. Based on Latin hypercube samples, the virtual patients are placed into the ADA-low and ADA-cohorts following the data ranges of ADA titre status realised in weeks 4, 8, 12 and 26 and the criteria for ADA-low and ADA-high patients (13-100 and > 100 AU/mL at any time respectively [10]), if their adalimumab concentration in weeks 4, 8, 12 and 26 lies in the reported range for the cohort.

Interpretation of the information criterion. The information criterion, introduced in equation (24) in the main text, is inspired by the Akaike Information Criterion (AIC), but it is not derived from a likelihood function. Rather, it is a heuristic score designed to compare competing mechanistic scenarios by balancing the discrepancy with the experimental data against the number of active MTX mechanisms. Consequently, the standard interpretation of ΔAIC values and the corresponding thresholds (e.g. the two-unit criterion proposed by Burnham and Anderson [5]) are not directly applicable.

Nevertheless, the criterion admits a straightforward interpretation in terms of the discrepancy measure. Consider two scenarios i and j, with information criteria

$$IC_i = n \ln\left(\frac{\Delta_i}{n}\right) + 2k_i, \text{ and } IC_j = n \ln\left(\frac{\Delta_j}{n}\right) + 2k_j \,, \tag{1}$$

where Δ_i and Δ_j denote the discrepancy measures, k_i and k_j the numbers of active mechanisms, and $n = 3$ the number of summary statistics.

Subtracting the two expressions yields

$$IC_i - IC_j = n \ln\left(\frac{\Delta_i}{\Delta_j}\right) + 2(k_i - k_j). \tag{2}$$

Therefore, when two scenarios have the same complexity ($k_i = k_j$), the difference between their information criteria depends only on the ratio of their discrepancy measures,

$$\frac{\Delta_i}{\Delta_j} = \exp\left(\frac{IC_i - IC_j}{n}\right). \tag{3}$$

As an illustration, scenarios 27 (APC only), 28 (activated T-cell apoptosis only), and 29 (NK-cell suppression only) each contain a single active mechanism. Since $k_{27} = k_{28} = k_{29} = 1$, no complexity penalty distinguishes these scenarios. Their reported information criteria are

$$IC_{28} = -11.951, \qquad IC_{27} = IC_{29} = -10.524,$$

which implies

$$\frac{\Delta_{27}}{\Delta_{28}} = \frac{\Delta_{29}}{\Delta_{28}} = \exp\left(\frac{1.427}{3}\right) \approx 1.61. \tag{4}$$

Hence, although the corresponding information criteria differ by only 1.43 units, the discrepancy measure of scenarios 27 and 29 is approximately 61% larger than that of scenario 28. This illustrates that relatively small differences in the information criterion may correspond to substantially larger differences in the underlying discrepancy because of the logarithmic transformation.

The complexity penalty also has a natural interpretation. Suppose that one additional biological mechanism is introduced, increasing the complexity term by two units. Such an increase is justified only if the reduction in discrepancy satisfies

$$n \ln\left(\frac{\Delta_{\text{new}}}{\Delta_{\text{old}}}\right) + 2 < 0, \tag{5}$$

which is equivalent to

$$\frac{\Delta_{\text{new}}}{\Delta_{\text{old}}} < e^{-2/n}. \tag{6}$$

Since $n = 3$ in the present study,

$$\frac{\Delta_{\text{new}}}{\Delta_{\text{old}}} < e^{-2/3} \approx 0.51. \tag{7}$$

Therefore, the inclusion of one additional mechanism is favoured only if it reduces the discrepancy measure by approximately 49%, thereby compensating for the increase in model complexity.

B Analytical solution for PK models

For an ADA-negative patient, there are no antibodies present ($A(t) = 0, \forall t > 0$), and we solve the system describing the adalimumab pharmacokinetics (8)-(9) for U_0, U analytically.

$$U_0' = D_U \sum_{j=0}^{n} \delta(t - \tau_U j) - a_U U_0, \quad U_0(0) = 0 \tag{8}$$

$$U' = a_U \frac{U_0}{V_U} - k_{101} A U - \delta_U U, \quad U(0) = 0 \; . \tag{9}$$

Assuming the initial doses to be $U_0(0) = 0, U(0) = 0$, we have

$$\begin{aligned}
U_0(t) &= D_U e^{-a_U t}, \quad 0 \le t < \tau_U \\
U_0(t) &= D_U (1 + e^{-a_U \tau_U}) e^{-a_U (t - \tau_U)}, \quad \tau_U \le t < 2\tau_U \\
U_0(t) &= D_U (1 + e^{-a_U \tau_U} + e^{-2 a_U \tau_U}) e^{-a_U (t - 2\tau_U)}, \quad 2\tau_U \le t < 3\tau_U \\
&\vdots \\
U_0(t) &= D_U \frac{1 - e^{-a_U (n+1) \tau_U}}{1 - e^{-a_U \tau_U}} e^{-a_U (t - n\tau_U)}, \quad n\tau_U \le t < (n+1)\tau_U
\end{aligned}$$

Substituting into the equation for U' we have

$$U(t) = \frac{a_U}{V_U} \left(\int_0^t U_0(s) e^{\delta_U s} \, ds \right) e^{-\delta_U t} \; ,$$

which can be computed in turn by splitting the integral over the intervals $[0, \tau_U), [\tau_U, 2\tau_U)$, etc. This approach gives us

$$\begin{aligned}
U(t) &= e^{-\delta_U t} \frac{a_U}{V_U} D_U \int_0^t e^{(\delta_U - a_U) s} ds = \frac{a_U}{V_U} D_U \frac{e^{-a_U t} - e^{-\delta_U t}}{\delta_U - a_U}, \quad 0 \le t < \tau_U \\
U(t) &= \frac{a_U}{V_U} e^{-\delta_U t} D_U \left(\int_0^{\tau_U} e^{(\delta_U - a_U) s} ds \right) + \frac{a_U}{V_U} (1 + e^{-a_U \tau_U}) e^{-\delta_U t} D_U \left(\int_{\tau_U}^{t} e^{-a_U (s - \tau_U) + \delta_U s} ds \right) \\
&= \frac{a_U}{V_U} D_U e^{-\delta_U t} \frac{e^{(\delta_U - a_U)\tau_U} - 1}{\delta_U - a_U} + \frac{a_U}{V_U} D_U (1 + e^{-a_U \tau_U}) \frac{e^{-a_U (t - \tau_U)} - e^{-\delta_U (t - \tau_U)}}{\delta_U - a_U}, \\
&\qquad \tau_U \le t < 2\tau_U, \\
U(t) &= \frac{a_U}{V_U} e^{-\delta_U t} D_U \left(\int_0^{\tau_U} e^{(\delta_U - a_U) s} ds \right) + \frac{a_U}{V_U} (1 + e^{-a_U \tau_U}) e^{-\delta_U t} D_U \left(\int_{\tau_U}^{2\tau_U} e^{-a_U (s - \tau_U) + \delta_U s} ds \right) \\
&+ \frac{a_U}{V_U} (1 + e^{-a_U \tau_U} + e^{-2 a_U \tau_U}) e^{-\delta_U t} D_U \left(\int_{2\tau_U}^{t} e^{-a_U (s - 2\tau_U) + \delta_U s} ds \right) \\
&= \frac{a_U}{V_U} D_U e^{-\delta_U t} \frac{e^{(\delta_U - a_U)\tau_U} - 1}{\delta_U - a_U} + \frac{a_U}{V_U} D_U (1 + e^{-a_U \tau_U}) e^{-\delta_U (t - \tau_U)} \frac{e^{(\delta_U - a_U)\tau_U} - 1}{\delta_U - a_U} \\
&+ \frac{a_U}{V_U} D_U (1 + e^{-a_U \tau_U} + e^{-2 a_U \tau_U}) \frac{e^{-a_U (t - 2\tau_U)} - e^{-\delta_U (t - 2\tau_U)}}{\delta_U - a_U}, \quad 2\tau_U \le t < 3\tau_U
\end{aligned}$$

By induction, the solution on the time interval $[n\tau_U, (n+1)\tau_U)$ takes the closed form

$$\begin{aligned}
U(t) &= \frac{D_U a_U}{V_U} \frac{e^{(\delta_U - a_U)\tau_U} - 1}{\delta_U - a_U} \sum_{i=0}^{n-1} \frac{1 - e^{-a_U(i+1)\tau_U}}{1 - e^{-a_U\tau_U}} e^{-\delta_U(t - i\tau_U)} \\
&+ \frac{D_U a_U}{V_U} \frac{1 - e^{-a_U(n+1)\tau_U}}{1 - e^{-a_U\tau_U}} \frac{e^{-a_U(t-n\tau_U)} - e^{-\delta_U(t-n\tau_U)}}{\delta_U - a_U} \\
&= \frac{D_U a_U}{V_U} \frac{e^{(\delta_U - a_U)\tau_U} - 1}{\delta_U - a_U} \left(\frac{1 - e^{\delta_U n\tau_U}}{(1 - e^{\delta_U\tau_U})(1 - e^{-a_U\tau_U})} - \frac{e^{-a_U\tau_U}(1 - e^{(\delta_U - a_U)n\tau_U})}{(1 - e^{-a_U\tau_U})(1 - e^{(\delta_U - a_U)\tau_U})} \right) e^{-\delta_U t} \\
&+ \frac{D_U a_U}{V_U} \frac{1 - e^{-a_U(n+1)\tau_U}}{1 - e^{-a_U\tau_U}} \frac{e^{-a_U(t-n\tau_U)} - e^{-\delta_U(t-n\tau_U)}}{\delta_U - a_U}, \quad n\tau_U \le t < (n+1)\tau_U \, .
\end{aligned}$$

For the MTX scheduling regime we have to change the formulation a little.

$$X_0' = \left(D_X \delta(t + 2\tau_X) + \sum_{j=0}^{n} D_X \delta(t - j\tau_X) \right) - a_X X_0, \quad X_0(-14) = 0 \tag{10}$$

$$X' = a_X \frac{X_0}{V_X} - \delta_X X, \quad X(-14) = 0, \tag{11}$$

For the initial conditions $X_0(-2\tau_X) = 0, X(-2\tau_X) = 0$, we have the following solution of (10)

$$\begin{aligned}
&X_0(t) = D_X e^{-a_X(t+2\tau_X)}, \quad -2\tau_X \le t < 0 \\
&X_0(t) = D_X(1 + e^{-2a_X\tau_X}) e^{-a_X t}, \quad 0 \le t < \tau_X \\
&X_0(t) = D_X(1 + e^{-a_X\tau_X} + e^{-3a_X\tau_X}) e^{-a_X(t-\tau_X)}, \quad \tau_X \le t < 2\tau_X \\
&\qquad \vdots \\
&X_0(t) = D_X \left(\frac{1 - e^{-na_X\tau_X}}{1 - e^{-a_X\tau_X}} + e^{-(n+2)a_X\tau_X} \right) e^{-a_X(t-(n-1)\tau_X)}, \\
&\qquad\qquad\qquad\qquad (n-1)\tau_X \le t < n\tau_X \, .
\end{aligned}$$

These expressions can be substituted into equation (11) for X', and we solve (11) for X using numerical integration because the closed form of the solution is algebraically involved.

C Parameter estimation from auxiliary low-dimensional models

In the parameter identification process we employ several targeted models in order to be estimate specific parameter values. Due to the sparsity of data points in the time series we use a nonlinear least squares approach, based on the FME toolbox [32], for this task.

Using small auxiliary models, evidence from the literature – further supported by the trends observed in the CoMARIS dataset – indicates that methotrexate (MTX) affects several immune cell populations, including activated T cells, antigen-presenting cells (APCs), B10 cells, and natural killer (NK) cells. To quantify these effects and estimate the corresponding parameters, we develop simple, targeted models focusing on each of these cell populations individually or in small interacting subsystems. The estimated parameters are then incorporated into the full model to ensure mechanistic consistency across scales.

C.1 ADA-adalimumab system

The available data does not contain information on the ADA isotype (IgM or IgG) produced during the administration of adalimumab. Since the units for $A(t)$ are μg/mL, but the ADA concentration has been evaluated in AU/mL, we have to covert the units. Indeed, based on the type of assay used, it is assumed that 1 AU $\sim$ 12 ng, and we convert all the values by the constant 0.012μg/AU [37, 4].

All data are subject to the assay's sensitivity range, with detection limits between 12 and 980 AU/m:. No values below 12 AU/mL were recorded, and values equal to 12 were interpreted as absence of antibodies. Some values above 980 AU/mL were present and carefully evaluated, as they may reflect true biological variability between patients.

Since the model (8) is a system of impulsive ODEs, solutions are not differentiable at all time points. Hence, using a least-squares minimisation or Bayesian Monte Carlo Markov Chain approach for parameter estimation of the binding rate k_{101} on few data would not produce satisfactory results and may cause convergence problems with the algorithm.

To circumvent such issues, we shall ignore the PK dynamics for adalimumab, and use an approximate, simplified system for its dynamics, which mimics a continuous infusion approach over time. We employ the following reduced model for the dynamics of adalimumab $U(t)$ and anti-drug antibody $A(t)$ in order to estimate the binding rate k_{101}

$$\begin{aligned} U' &= \beta_U - k_{101}AU - \mu_U U, \quad U(0) = 0 \\ A' &= \sigma_0 U + \sigma_1 A\left(1 - \frac{A}{K_a}\right), \quad A(0) = 0 \end{aligned} \tag{12}$$

In (12) the source term β_U is measured in μg/(mL day), and μ_U is the clearance rate obtained from the model fit, and not from the reported PK values in the literature. We also assume longevity of the antibody-producing plasma cells, so the dynamics of anti-drug antibody can be approximated by a logistic equation, as long as $U > 0$. Also we assume that the dynamics (12) would approximate the process for low-antibody titre (the patient cohort classified as having antidrug antibody titre less than 100 AU/mL). We ignore the removal of antibody due to formation of complexes.

We have a separation in the data on distribution of the antibody titres at week 28 within the *ADA-low* group (first sub-cohort with $<$ 30 AU/mL with multiple counts below the limit of detection of the assay, and second sub-cohort with titres between 30 AU/mL and 100 AU/mL). Hence, we use the data from the second sub-cohort to estimate the value of k_{101}, and assume a small value for $\sigma_0 = 0.001$.

A value $\sigma_0 = 0$ produces results for the model without antibodies ($A \equiv 0$) and estimates for β_U, μ_U can be readily obtained. The parameter fit is performed simultaneously using the mean values from the *ADA-negative*-MTX– cohort ($\sigma_0 = 0$) and the *ADA-low*-MTX– cohort with final ADA titres between 30 AU/mL and 100 AU/mL ($\sigma_0 = 0.001$). We estimate β_U, μ_U from the data of the *ADA-negative*-MTX– patients, as well as the effect of the antibody binding to its target simultaneously. The results are presented in Table A.

Parameter	Unit	Estimate	Std. Error	t-value	p-value
k_{101}	(μg/mL day)$^{-1}$	0.166	0.127	1.306	0.221
σ_1	(day)$^{-1}$	0.027	0.035	0.785	0.450
β_U	μg/(mL day)	0.230	0.063	3.644	0.005
μ_U	(day)$^{-1}$	0.025	0.009	2.826	0.018
K_a	μg/mL	1.120	0.856	1.401	0.191

Table A: Estimates of parameters for model (12).

C.2 Estimation of the PK parameters for adalimumab V_U, δ_U

We perform the estimation using a nonlinear least squares method for the adalimumab PK model with $a_U = 0.28, D_U = 50(\times 10^3)$μg/mL, we have the estimates for V_U and δ_U shown in Table B for the two cohorts of ADA-negative patients across all visits, respectively MTX– and MTX+.

Table B: Estimation of V_U and δ_U for the group of ADA-negative patients across all visits.

Parameter	Unit	Mean	Std. Error	t-value	p-value
		MTX– patients			
V_U	dimensionless	10.2021×10^3	238	428.21	$< 10^{-4}$
δ_U	(day)$^{-1}$	0.0394	0.0015	26.78	0.0001
		MTX+ patients			
V_U	dimensionless	10.03×10^3	95	1049.40	$< 10^{-4}$
δ_U	(day)$^{-1}$	0.03191	0.001	32.19	$< 10^{-4}$

We see that the estimates of V_U, δ_U in both ADA-negative cohorts are similar.

C.3 Estimation of the MTX dilution coefficient V_X

Since data on MTX concentration in the central compartment (blood) are not available, we use the values for the adalimumab dilution coefficient V_U for the same virtual patients, but must compute the respective dilution coefficient V_X for MTX from the mass balance equation.

If clearance of U, X is neglected, for a single injection we have

$$\lim_{t\to+\infty} U(t) = \frac{D_U}{V_U}, \quad (13)$$

$$\lim_{t\to+\infty} X(t) = \frac{D_X}{V_X}. \quad (14)$$

Then due to the 4:1 ratio between the adalimumab mass and the MTX mass in a single injection, from the equation [mass]=[concentration]×[volume] we obtain

$$\frac{D_U}{V_U} \times [\text{blood volume}] = 4\left(\frac{D_X}{V_X} \times [\text{blood volume}]\right),$$

that simplifies to

$$V_X = 4\frac{D_X}{D_U}V_U,$$

or finally,

$$V_X = 0.8V_U \quad \text{for } D_X = 10 \times 10^3 \mu\text{g/mL}.$$

C.4 Estimation of the MTX-induced apoptotic effect on activated T lymphocytes

The authors of the study [12] examine the effect of MTX on viable cell recovery *in vitro*. T lymphocytes were stimulated with antigen for three days, and then MTX was administered to samples at different concentrations: 0.01 to 10 μM.

The generated PK dynamics of MTX (11) indicates that the range of free MTX in patients' plasma is 0.2-0.8 μg/mL, so we use a model fit with MTX dose $X = 0.045, 0.45 \mu\text{g/mL}$ (equivalent to $0.1, 1 \mu\text{M}$). The equation governing lymphocyte growth in the absence of MTX ($X = 0$) is a logistic growth law (16) with $T(t)$ being the population size of viable cells at time t ($\times 10^5$ cells) and initial data $T(0) = 1$. The apoptotic action of MTX on activated T lymphocytes is modelled using a Hill function

$$\phi_1(X) = \frac{\gamma_4 X^2}{c_4^2 + X^2}, \quad (15)$$

so the dynamics of the *in-vitro* system is described by

$$T' = aT - bT^2 - \phi_1(X)T, \quad T(0) = 1. \quad (16)$$

We perform a parameter estimation for the three data series from [12] simultaneously.

In Table C, we summarize the estimated values for a, b, and γ_4, c_4.

Parameter	Unit	Estimate	Std. Error	t-value	p-value
a	$(\text{day})^{-1}$	0.706	0.165	4.27	0.002
b	$(10^5 \text{ cells}\times\text{day})^{-1}$	0.357	0.106	3.34	0.01
γ_4	$(\text{day})^{-1}$	0.704	0.102	6.89	0.0001
c_4	$(\text{day})^{-1}$	0.015	0.006	2.75	0.025

Table C: Parameter estimates for model (16).

Consequently, we use the function ϕ_1 with the parameters listed in (16) in the corresponding equations in the full model.

C.5 Estimation of the MTX-induced apoptotic effect on activated B lymphocytes

The author of the study [20] examines the effect of MTX on proliferation and apoptosis of B lymphocytes *in vitro*. $CD19^+$ B lymphocytes were treated with MTX at concentration 2 µM (0.9 µg/mL) for 24 hours prior to stimulation with CD40 ligand.

The study reports that for $CD19^+IgD^+CD27^-$ cells (naive B lymphocytes), apoptosis increases rapidly at 96 hours (43.1 ± 10.2% versus 8.3 ± 3.7% in MTX-untreated cells, p-val $< 10^{-4}$), whereas for $CD19^+IgD^-CD27^+$ (B memory) cells, apoptosis increases significantly already at 48 hours compared to untreated cells (82.8 ± 7.5% versus 14.1 ± 6.2%, p-val $< 10^{-4}$).

Using an exponential decay model for the scaled viable and apoptotic naive B cell populations $C_n(t), A_n(t) = 1 - C_n(t)$:

$$C_n(T_{end}) = e^{-(c_{40}X+\mu_n)T_{end}},$$
$$A_n(T_{end}) = 1 - e^{-(c_{40}X+\mu_n)T_{end}}, X = 0, 0.9 \mu\text{g/mL}, T_{end} = 4 \text{ days},$$

and for the viable and apoptotic B memory cell populations $C_m(t), A_m(t) = 1 - C_m(t)$

$$C_m(T_{end}) = e^{-(c_{41}X+\mu_m)T_{end}},$$
$$A_m(T_{end}) = 1 - e^{-(c_{41}X+\mu_m)T_{end}}, X = 0, 0.9 \mu\text{g/mL}, T_{end} = 2 \text{ days},$$

we estimate the parameters for the death rates μ and the rate for MTX-induced apoptosis c as follows: $\mu_n = 0.021$ day^{-1}, $c_{40} = 0.133$ (µg/mL day)$^{-1}$, $\mu_m = 0.076$ day^{-1}, $c_{41} = 0.893$ (µg/mL day)$^{-1}$.

C.6 Estimation of the effect of MTX on APC differentiation

As demonstrated in [30], MTX acts on the differentiation of immature and undifferentiated monocytes. Using the data on human monoblastic leukemia U937 cell line, which differentiates into macrophage-like cells from the study [30], we estimate the effect of MTX on the production rate of immature/resting APCs $D_0(t)$ to calibrate the functional response to MTX in the model.

We use the following growth law

$$D_0' = \mu_D(D_{max} - D_0) - \gamma_1 D_0 X \tag{17}$$

to model the suppressive effect of MTX (X) on the differentiation of the U937 cells towards macrophages. We use the value $\mu_D = 0.2$ from the Table in Section D

The estimated values for the parameters $X = 0$, 9.08 ng/mL $[0, 2 \times 10^{-8}$ Mol/L] are summarised in Table D.

Parameter	Unit	Estimate	Std. Error	t-value	p-value
D_{max}	cells	2.966	0.13	22.6	$< 10^{-5}$
γ_1	(ng/mL day)$^{-1}$	0.2094	0.06	3.482	0.013
	(µg/mL day)$^{-1}$	209.4	60	3.482	0.013

Table D: Parameter estimates for model (17).

C.7 Estimation of the effect of MTX on regulatory B cell activation

Rescaling the equation for regulatory B cells of the model in the main text via letting $z = \frac{B_{10}}{K_{10}}$, we have

$$z' = \mu_{10}(1 + \alpha_{10} X - z)\ . \tag{18}$$

We used patient data on CD24^{high}CD27^{+} cells to estimate the parameter values of α_{10} in equation (18), using the dose scheduling of 10 mg/mL per week. Because the kinetics of MTX is much faster than the lymphocyte population kinetics, and due to the sparsity of data points (4 in total), we assume that MTX is administered continuously at a dose $\bar{X}(t) \equiv 0.066$ mg/mL, $\forall t \geq 0$ in (18). The value $\bar{X}$ is obtained by averaging the solution $X(t)$ of (11) over the duration of therapy (parameter values used are $D_X = 10, V_X = 8, a_X = 8.64, \delta_X = 2.07$).

The results are summarised in Table E.

Parameter	Unit	Mean	Std. Error	t-value	p-value
	group average ($V_X = 8.0, D_X = 10$ mg/mL)				
α_{10}	(mg/mL)$^{-1}$	9.393	0.88	10.6	0.002

Table E: Parameter estimates for model (18).

C.8 Estimation of the suppressive effect of MTX on NK cells

For the parameter estimation, we assume TNFα is at equilibrium and the model (19) is fit to CoMARIS data with two values of F_α. In the MTX+ group we assume $F_\alpha \approx 0$ (due to the action of TNF inhibitor), and in the MTX– group we set $F_\alpha \approx \kappa_{\text{TNF}}$. Estimates are made by assuming that the MTX dose is administered continuously at $X(t) \equiv 0.066, \forall t \geq 0$ as done in the previous section.

$$\begin{aligned} K_0' &= \mu_{NK}(K_{NK} - K_0) - \gamma_2 X K_0 \\ K_1' &= \alpha_2 \left(1 + \frac{F_\alpha}{F_\alpha + \kappa_{\text{TNF}}}\right)(K_0 - K_1) - \mu_{NK} K_1 - \gamma_3 X K_1 \end{aligned} \tag{19}$$

The results are summarised in Table F.

Parameter	Unit	Estimate	Std. Error	t-value	p-value
		MTX^{-}			
α_2	day^{-1}	0.05			
		MTX^{+} $D_X = 10$ mg/mL$, V_X = 8.0$			
α_2	day^{-1}	0.1167			
γ_2	(µg/mL day)$^{-1}$	0.323	0.026	12.3	$< 10^3$
γ_3	(µg/mL day)$^{-1}$	0.423	0.138	3.07	0.022

Table F: Parameter estimates for model (19).

D Model Parameters

Table G: Parameters describing the dynamics of antigen presenting cells (APCs).

Parameter	Description	Units	Value (±SE)/Range		Reference
μ_D	death rate, naive APC	(day)$^{-1}$	0.2		[27]
μ_{D_1}	death rate, mature APC	(day)$^{-1}$	0.75		[14]
α_1	maturation rate, APC	(day)$^{-1}$	0.5		[36]
K_D	carrying capacity, APC	cells/mL	50000		[9]
$\alpha_{\mathrm{APC}\vert\mathrm{U}}$	loading of APC with antigen	(µg/mL day)$^{-1}$	transient	0.01	similar to [14]
			ADA-pos	0.025	
γ_1	suppressive effect of MTX on APC	(µg/mL day)$^{-1}$	209.4 ± 60		Table D

Table H: Parameters describing the dynamics of adalimumab, methotrexate and TNFα.

Parameter	Description	Units	Value (±SE)/Range	Reference
κ_{TNF}	Michaelis constant, TNFα[1]	µg/mL	$9.7 \times 10^{-6}; 14 \pm 11 (\times 10^{-6})$	[8, 33, 13]
a_U	absorption rate, subcutaneous adalimumab	(day)$^{-1}$	0.28	[34]
δ_U	removal rate, adalimumab	(day)$^{-1}$	0.03	[39], Table B
δ_X	removal rate, methotrexate	(day)$^{-1}$	$(2.08, 3.33)$	[3, 16]
a_X	absorption rate, subcutaneous methotrexate	(day)$^{-1}$	8.64	[18]
V_U	dilution coefficient, depot to central compartment, adalimumab	dimensionless	$(8 \times 10^3, 12 \times 10^3)$	Table B
V_X	dilution coefficient, depot to central compartment, MTX	dimensionless	depends on V_U	Section C
p_A	production rate, anti-drug antibody (ADA)	(µg/cell)/day	$2.69 \times 10^{-3}, 4 \times 10^{-3}$	[17]
k_{100}	binding rate, TNFi to TNFα	(µg/mL day)$^{-1}$	1.01×10^3	[21]
k_{101}	binding rate, ADA to TNFi	(µg/mL day)$^{-1}$	0.166 ± 0.127	Table A
δ_A	clearance rate, antibody	(day)$^{-1}$	2.07	[31]
δ_α	TNFα clearance rate	(day)$^{-1}$	161.5 $(124, 199)$	[41]

[1] Corresponding to the measured concentration in AxSpA patients reported in the literature.

κ_0	background production rate of TNFα	(µg/mL)/day	10^{-6}	similar to [28]
κ_1	TNFα production by APC	(µg/cell)/day	$10^{-9}(9.6 \times 10^{-10}, 1.6 \times 10^{-8})$	[28]
κ_3	TNFα production by $CD4^+$ T cells	(µg/cell)/day	$10^{-9}(1.5 \times 10^{-10}, 1.9 \times 10^{-9})$	[28]
κ_5	TNFα production by mature NK cells	(µg/cell)/day	2.5×10^{-8}	[28]

Table I: Parameters describing the dynamics of T lymphocytes.

Parameter	Description	Units	Value (±SE)/Range		Reference
$\bar{H}_0(0)$	initial supply, naive T cells	cells/mL	10000-24000		data $CD4^+CD25^-$
ρ_{Ha}	maximum proliferation rate, activated T cell	$(\text{day})^{-1}$	0.05		similar to [24]
ρ_{Hm}	maximum proliferation rate, activated memory T cell	$(\text{day})^{-1}$	ADA-pos	0.047	similar to [6, 25]
			transient	0	
$\alpha_{\mathrm{T\|APC}}$	maximum activation rate of T cell by APC	$(\text{day})^{-1}$	ADA-low	0.003 ± 0.001	fit to data
			ADA-high	0.004 ± 0.001	
			transient	0.001 ± 0.00015	
$\alpha_{\mathrm{T_m\|APC}}$	maximum activation rate of memory T cell by APC	$(\text{day})^{-1}$	ADA-low	0.004 ± 0.001	fit to data
			ADA-high	0.004 ± 0.001	
			transient	0	
μ_{Ha}	Death rate, activated $CD4^+$ T cell	$(\text{day})^{-1}$	0.05		similar to [24, 25]
μ_{He}	Death rate, effector T cell	$(\text{day})^{-1}$	0.113		[25]
K_{Hm}	carrying capacity, memory T cell	cells/mL	ADA-low	100	assumed
			ADA-high	100	
p	Probability of differentiation to memory T cell	dimensionless	ADA-pos	0.5	similar to [6]
			transient	0	
q	Probability of proliferation, activated T cell	dimensionless	ADA-pos	0.5	similar to [6]
			transient	0.9	
h_n	weight for activation threshold for naive T cells by APC	dimensionless	ADA-low	0.001 ± 0.0002	fit to data
			ADA-high	0.01 ± 0.002	
			transient	0.0005 ± 0.00005	
h_m	weight for activation threshold for memory T cells by APC	dimensionless	ADA-low	0.0009 ± 0.0002	fit to data
			ADA-high	0.0093 ± 0.002	
K_{NK}	supply of NK cells	(cells/mL)/day	MTX−	10^5	data
			MTX+	1.2×10^5	
α_2	maximum maturation rate NK cells		MTX−	0.05	Table F
			MTX+	0.1167	
μ_{NK}	death rate, NK cells	$(\text{day})^{-1}$	0.05		[42]

Table J: Parameters describing the dynamics of B lymphocytes.

Parameter	Description	Units	Value (±SE)/Range		Reference
$\bar{B}_0(0)$	initial supply, naive B cells	cells/mL	2000-5000		data
ρ_{Ba}	maximum proliferation rate, activated B cell	(day)$^{-1}$	ADA-pos	0.35	[11]
			transient	0.29 ± 0.03	fit to data
ρ_{Bm}	maximum proliferation rate, memory B cell	(day)$^{-1}$	ADA-pos	0.5	similar to [11]
			transient	0	
$\alpha_{\mathrm{B}\vert\mathrm{T}}$	maximum activation rate of B cell by effector T cell	(day)$^{-1}$	ADA-pos	0.1 ± 0.014	similar to [6], fit to data
			transient	0.006 ± 0.0001	
$\alpha_{\mathrm{Bm}\vert\mathrm{T}}$	maximum activation rate of memory B cell by effector T cell	(day)$^{-1}$	ADA-low	0.15 ± 0.014	fit to data
			ADA-high	1.25 ± 0.5	
			transient	0	
μ_{Ba}	death rate, activated B cell	(day)$^{-1}$	0.032		[26]
K_{Bm}	carrying capacity, memory B cell	cells/mL	ADA-low	$[10, 100]$	assumed
			ADA-high	100	
r	Probability of differentiation of activated B to memory B cell	dimensionless	ADA-pos	0.5	similar to p
			transient	0	
s	Probability of proliferation, activated B cells	dimensionless	ADA-pos	0.5	similar to q
			transient	0.9	
μ_{Ps}	Death rate, short-lived plasma cell	(day)$^{-1}$	0.13		[22]
μ_P	Death rate, long-lived plasma cell	(day)$^{-1}$	0.01		similar to [6]
ρ_{Ps}	Rate of differentiation to short-lived plasma	dimensionless	ADA-low	1 ± 0.41	fit to data
			ADA-high	0.25	
			transient	3.08 ± 0.56	
b_n	weight for activation threshold for naive B cells by effector T cells	dimensionless	ADA-low	0.79 ± 0.23	fit to data
			ADA-high	0.54 ± 0.25	
			transient	0.0013 ± 0.0002	
b_m	weight for activation threshold for memory B cells by effector T cells	dimensionless	ADA-low	1.18 ± 0.47	fit to data
			ADA-high	0.36 ± 0.19	
K_{10}	supply Breg cells	cells/mL	1		scaled
μ_{10}	proliferation rate B10 cells	(day)$^{-1}$	0.032		assumed $= \mu_{Ba}$

Since each virtual cohort is selected according to parameter values drawn from a Latin hypercube, we perform a test for correlation between pairs of parameters. Table K shows the Pearson correlation coefficients for those pairs with statistical significance at level 1% in the ADA-low, ADA-high and ADA-transient virtual patient cohorts.

Table K: Pearson's correlation coefficients for parameter pairs with statistical significance at level 1%. Values in brackets indicate the 99% confidence intervals.

Cohort	Parameter pair	Pearson's (r)
ADA-low	$(\alpha_{\mathrm{T\|APC}}, b_m)$	0.311 (0.116, 0.483)
	(b_n, b_m)	-0.334 (-0.503, -0.141)
	$(\alpha_{\mathrm{T\|APC}}, b_n)$	0.411 (0.227, 0.566)
ADA-high	$(\alpha_{\mathrm{B\|T}}, h_n)$	0.205 (0.047, 0.353)
	$(\alpha_{\mathrm{B\|T}}, h_m)$	0.198 (0.040, 0.346)
	$(\alpha_{\mathrm{B\|T}}, b_m)$	0.348 (0.200, 0.481)
	$(\alpha_{\mathrm{B_m\|T}}, h_n)$	0.482 (0.350, 0.595)
	$(\alpha_{\mathrm{B_m\|T}}, h_m)$	0.483 (0.350, 0.596)
	$(\alpha_{\mathrm{T\|APC}}, b_n)$	0.302 (0.150, 0.440)
	$(\alpha_{\mathrm{T\|APC}}, b_m)$	0.377 (0.231, 0.506)
	(h_n, h_m)	0.941 (0.921, 0.957)
ADA-transient	No significant correlations	

E Supplementary Figures

E.1 Cell counts

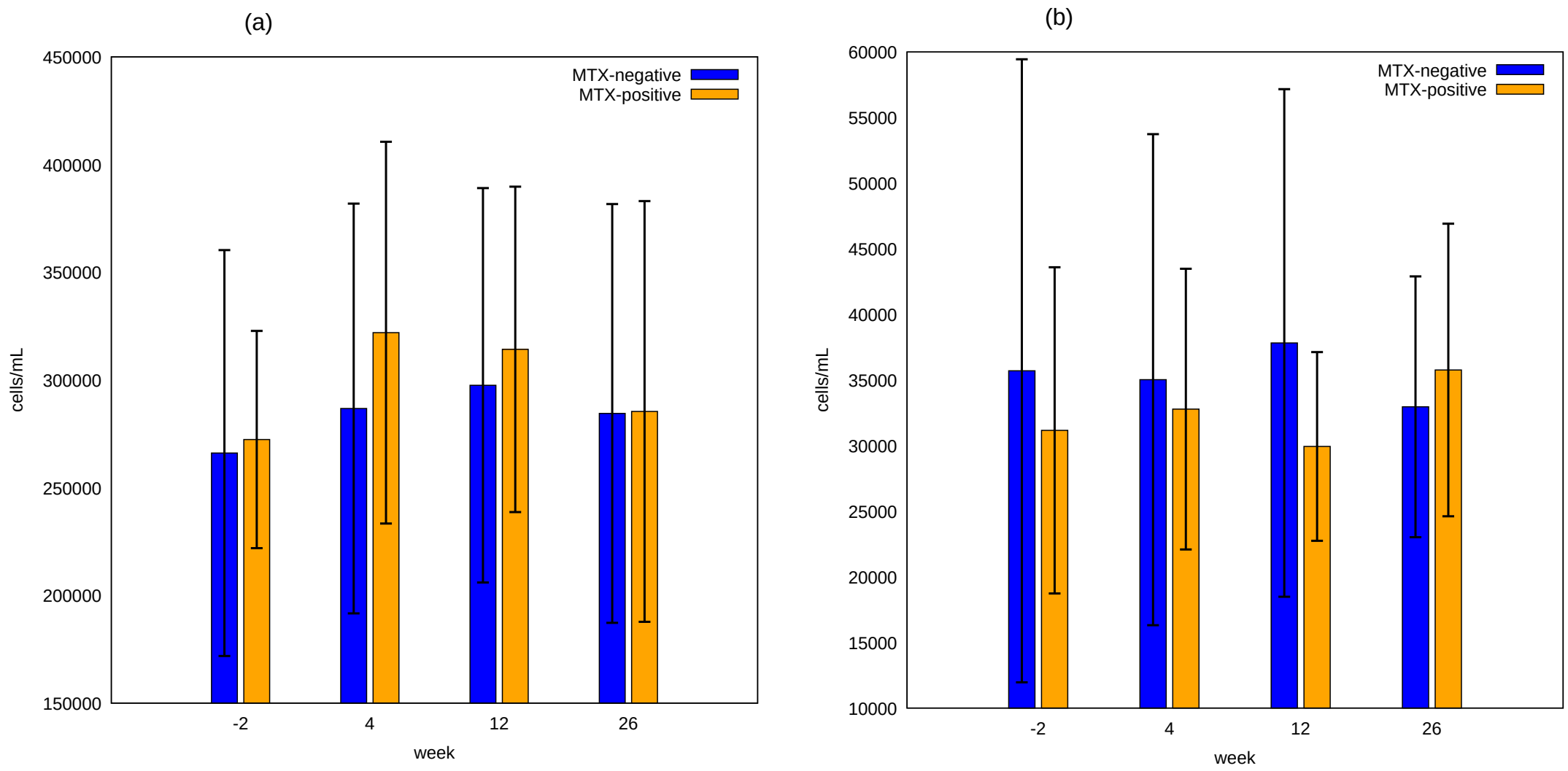

Fig A: Cell counts of (a) naive CD4$^+$CD25$^-$ T lymphocytes, (b) CD4$^+$CD25$^+$ T lymphocytes (mean $\bar{\mu}$ and standard error) in the patients who received only adalimumab ($MTX-$, 11 patients) or co-administration of methotrexate ($MTX+$, 8 patients). No statistically significant differences in the MTX+ group.

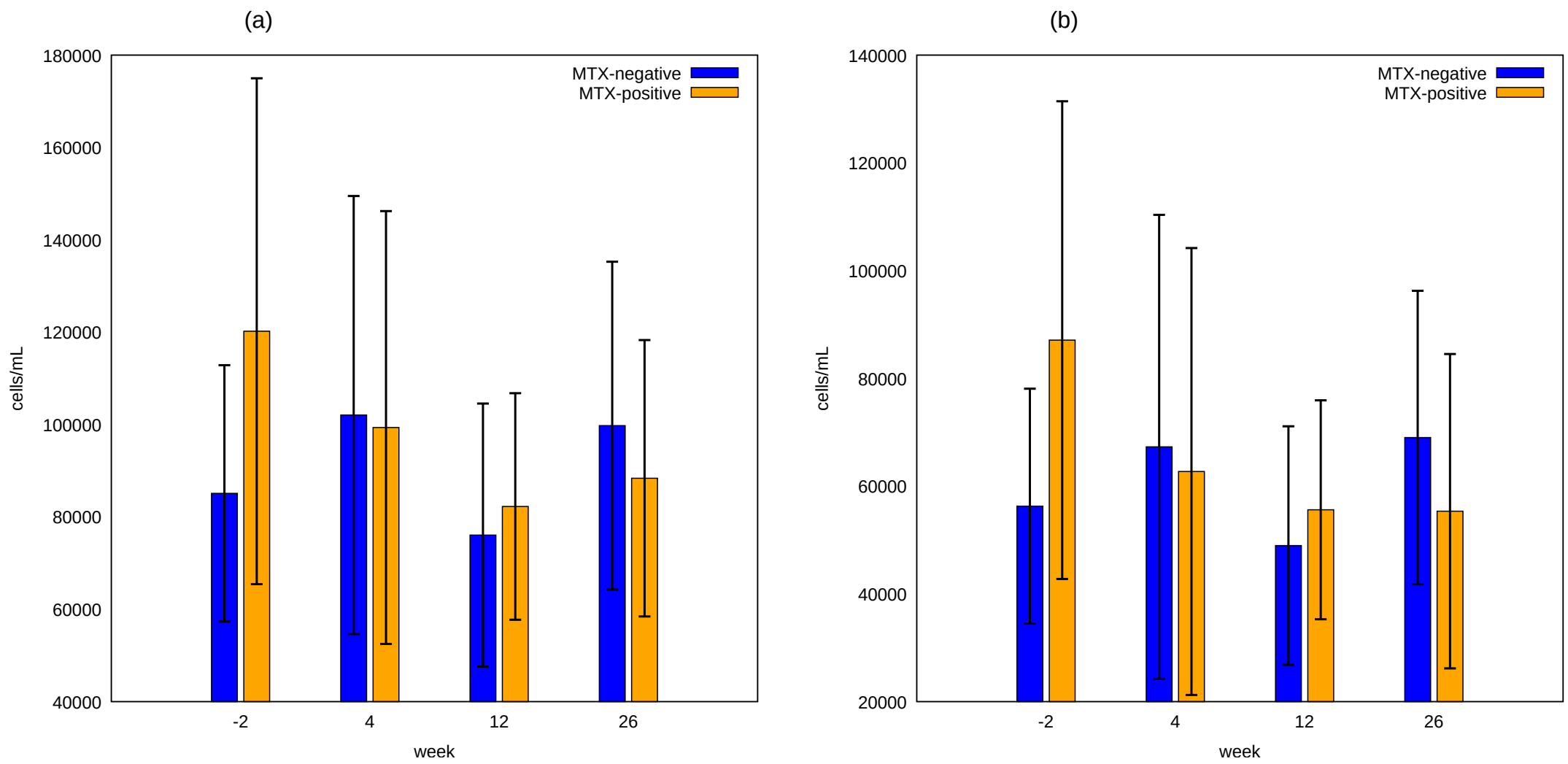

Fig B: Natural killer (NK) cell counts (mean $\bar{\mu}$ and standard error) in the patients who received only adalimumab (MTX–, 11 patients) or co-administration of methotrexate (MTX+, 8 patients). (a) **NK cells ($CD3^-CD56^+$):** In the MTX+ group, the null hypothesis $H_0 : \bar{\mu}_{-2} \leq \bar{\mu}_4$ is rejected at 5% (p-value=0.027), $H_0 : \bar{\mu}_{-2} \leq \bar{\mu}_{12}$ is rejected at 5% (p-value=0.022), and $H_0 : \bar{\mu}_{-2} \leq \bar{\mu}_{26}$ is rejected at 5% (p-value=0.027). (b) **Mature NK cells ($CD3^-CD56^+CD16^+$):** In the MTX+ group, the null hypothesis $H_0 : \bar{\mu}_{-2} \leq \bar{\mu}_4$ is rejected at 1% (p-value=0.007), $H_0 : \bar{\mu}_{-2} \leq \bar{\mu}_{12}$ is rejected at 5% (p-value=0.031), and $H_0 : \bar{\mu}_{-2} \leq \bar{\mu}_{26}$ is rejected at 5% (p-value=0.014).

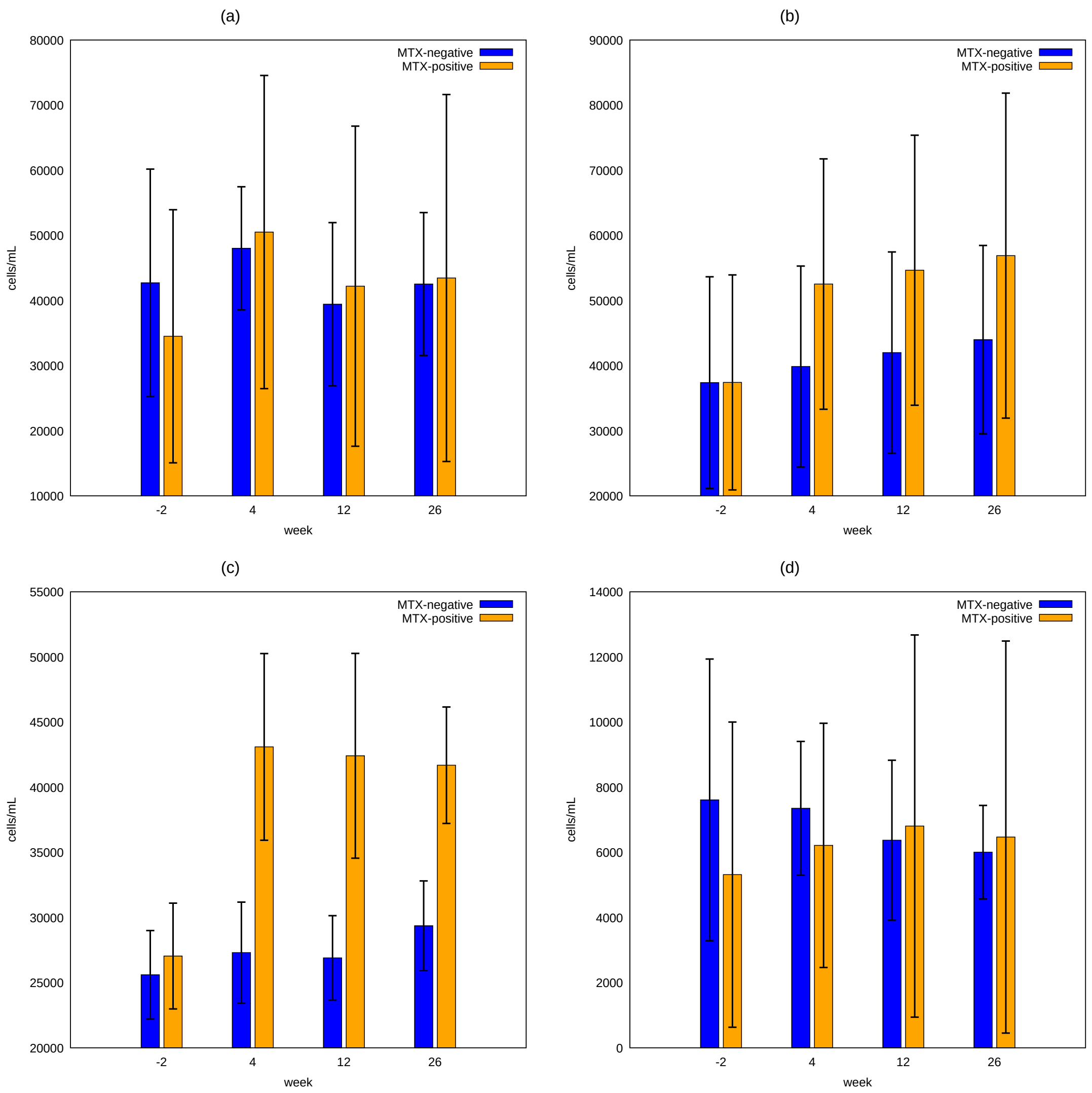

Fig C: Cell counts of the B lymphocyte subsets (mean $\bar{\mu}$ and standard error) in the patients who received only adalimumab (*MTX–*, 11 patients) or co-administration of methotrexate (*MTX+*, 8 patients). Differences between week -2 and week $i = 4, 12, 26$ were tested using the paired t-test. (a) **Naive B $CD19^{+}CD27^{-}$ cells:** No statistically significant differences in the MTX+ group. (b) **B memory $CD19^{+}CD27^{+}$ cells:** In the MTX+ group, the null hypothesis $H_0 : \bar{\mu}_{-2} \geq \bar{\mu}_4$ is rejected at 10% (p-value=0.059), $H_0 : \bar{\mu}_{-2} \geq \bar{\mu}_{12}$ is rejected at 5% (p-value=0.021), and $H_0 : \bar{\mu}_{-2} \geq \bar{\mu}_{26}$ is rejected at 5% (p-value=0.033). (c) **$CD24^{hi}CD27^{+}$ cells:** No statistically significant differences in the MTX– group. In the MTX+ group, the null hypothesis $H_0 : \bar{\mu}_{-2} \geq \bar{\mu}_4$ is rejected at 5% (p-value=0.022), the null hypothesis $H_0 : \bar{\mu}_{-2} \geq \bar{\mu}_{12}$ is rejected at 5% (p-value=0.04), and the null hypothesis $H_0 : \bar{\mu}_{-2} \geq \bar{\mu}_{26}$ is rejected at 5% (p-value=0.026). (d) **$CD24^{+}CD38^{hi}$ cells:** No statistically significant differences in either group.

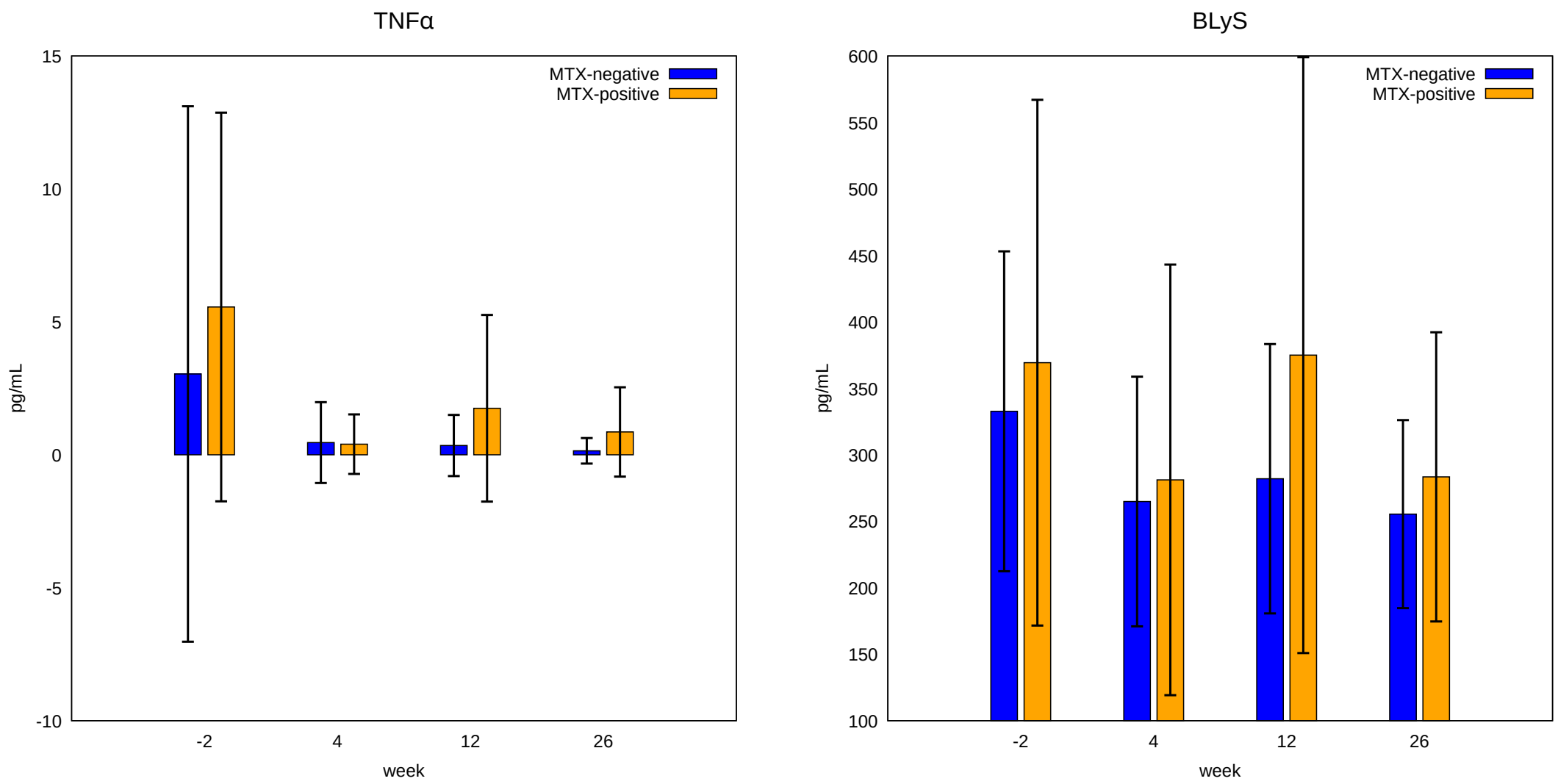

Fig D: Serum data (mean $\bar{\mu}$ and standard error): **TNFα:** *MTX-* 10/11 patients below LOD at all visits, *MTX+*: 4/8 patients below LOD at all visits. **BLyS:** No statistically significant differences in MTX+ group.

E.2 Model-predicted adalimumab and ADA dynamics under MTX co-admini-stration

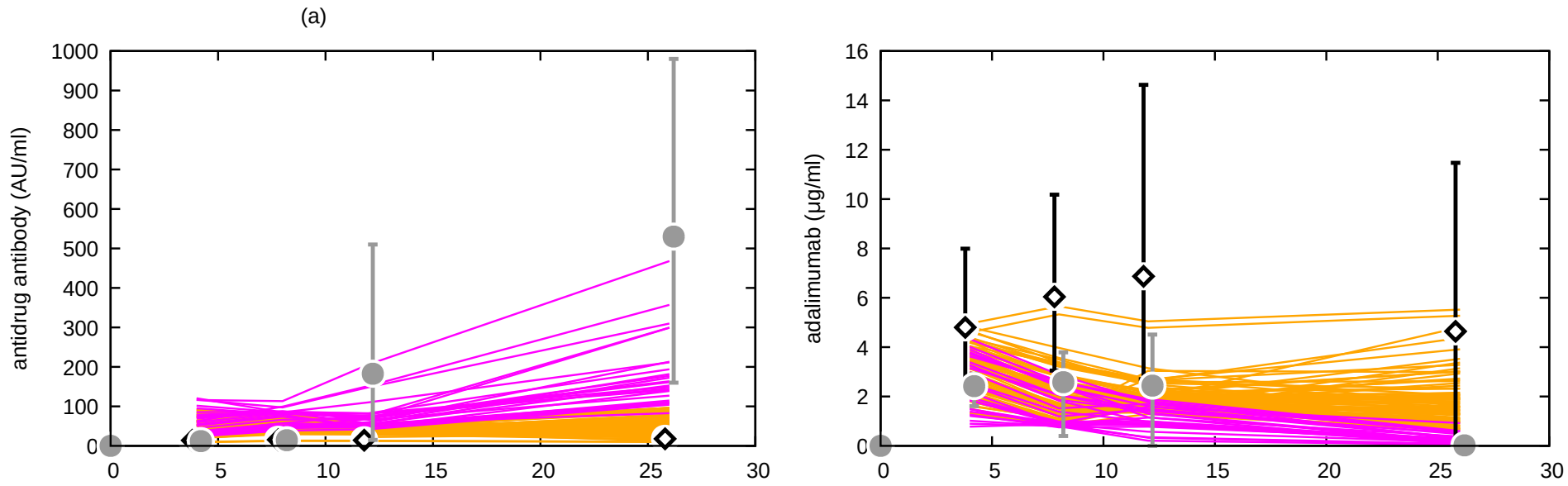

Fig E: Profiles of antidrug antibody and adalimumab for MTX+ virtual patients from ADA-low cohort (orange) and ADA-high cohort (magenta) under scenario no. 13 (MTX effect on activated T and B lymphocytes only). Error bars represent minimum and maximum for 14 MTX+ ADA-low patients (black ⋄), 3 MTX+ ADA-high patients (grey •) in the CoMARIS data.

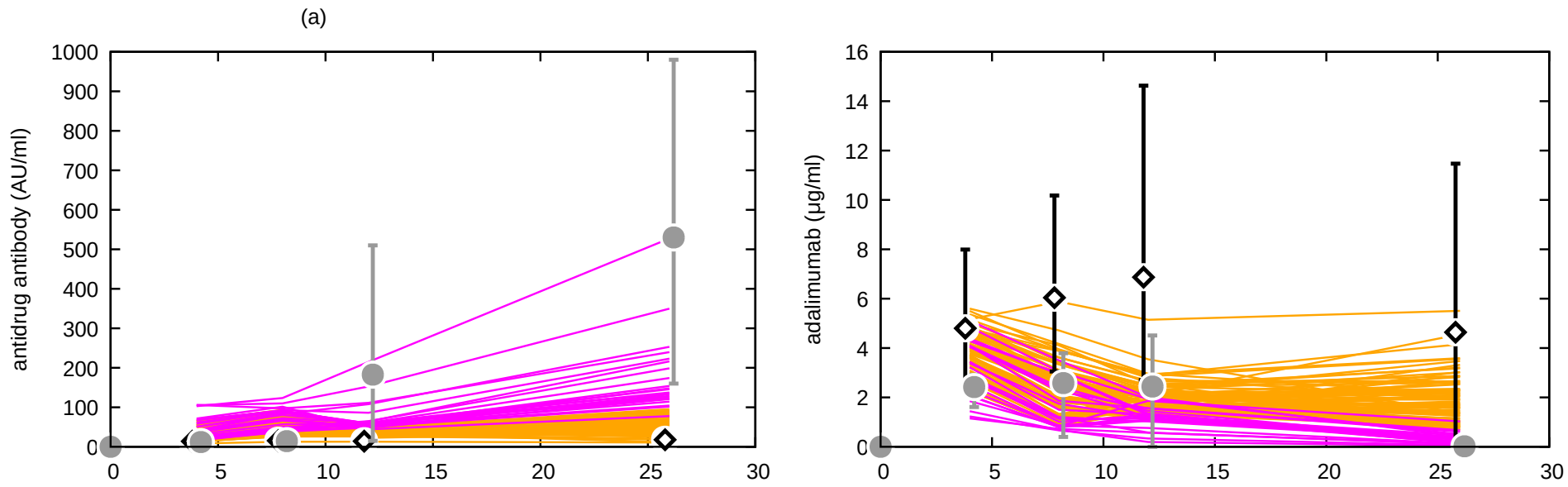

Fig F: Profiles of antidrug antibody and adalimumab for MTX+ virtual patients from ADA-low cohort (orange) and ADA-high cohort (magenta) under scenario no. 21 (MTX effect on APCs and activated T lymphocytes). Error bars represent minimum and maximum for 14 MTX+ ADA-low patients (black ⋄), 3 MTX+ ADA-high patients (grey •) in the CoMARIS data.

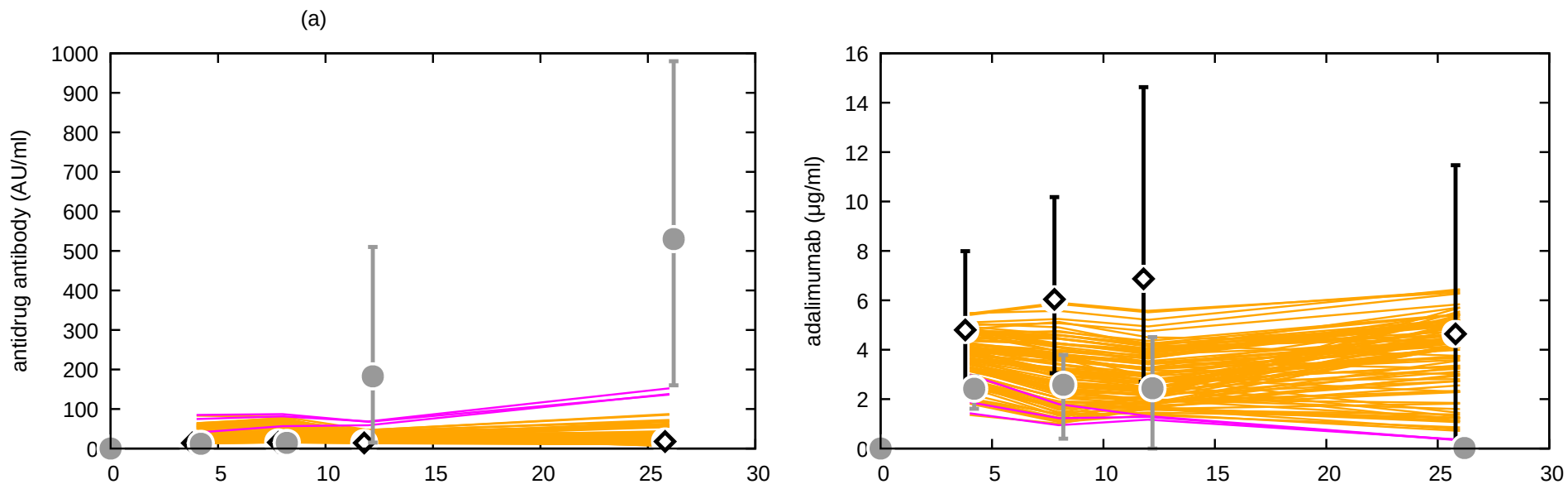

Fig G: Profiles of antidrug antibody and adalimumab for MTX+ virtual patients from ADA-low cohort (orange) and ADA-high cohort (magenta) under scenario no. 25 (MTX effect on activated T lymphocytes and B regs). Error bars represent minimum and maximum for 14 MTX+ ADA-low patients (black ⋄), 3 MTX+ ADA-high patients (grey •) in the CoMARIS data.

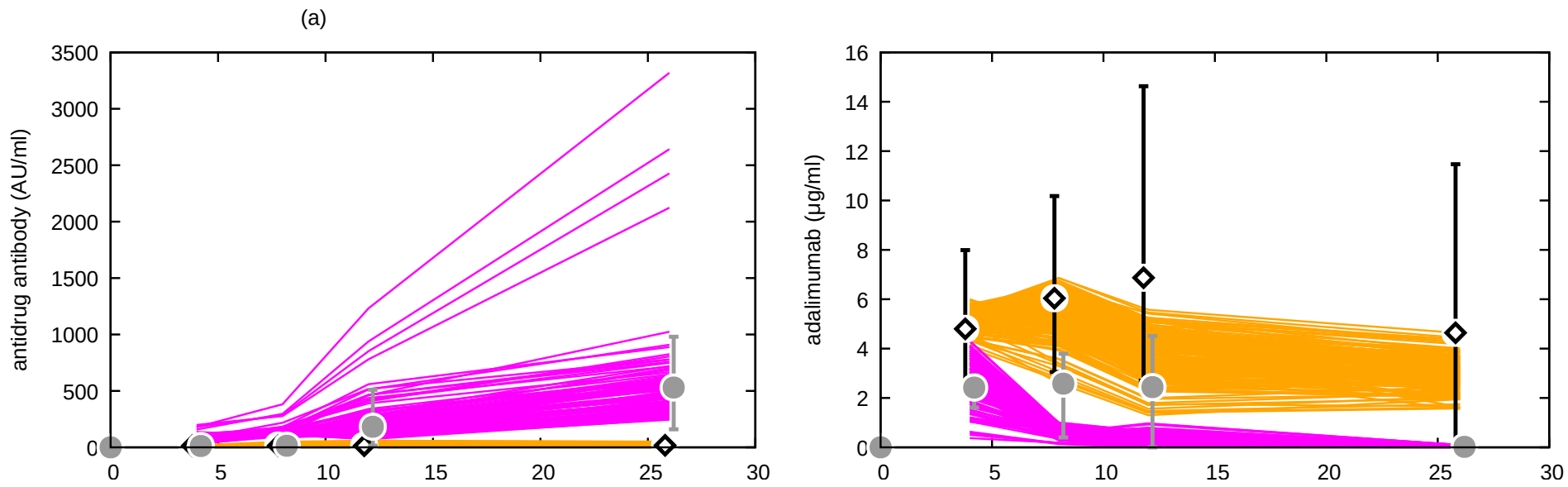

Fig H: Profiles of antidrug antibody and adalimumab for MTX+ virtual patients from ADA-low cohort (orange) and ADA-high cohort (magenta) under scenario no. 27 (MTX effect on APCs only). Error bars represent minimum and maximum for 14 MTX+ ADA-low patients (black ⋄), 3 MTX+ ADA-high patients (grey •) in the CoMARIS data.

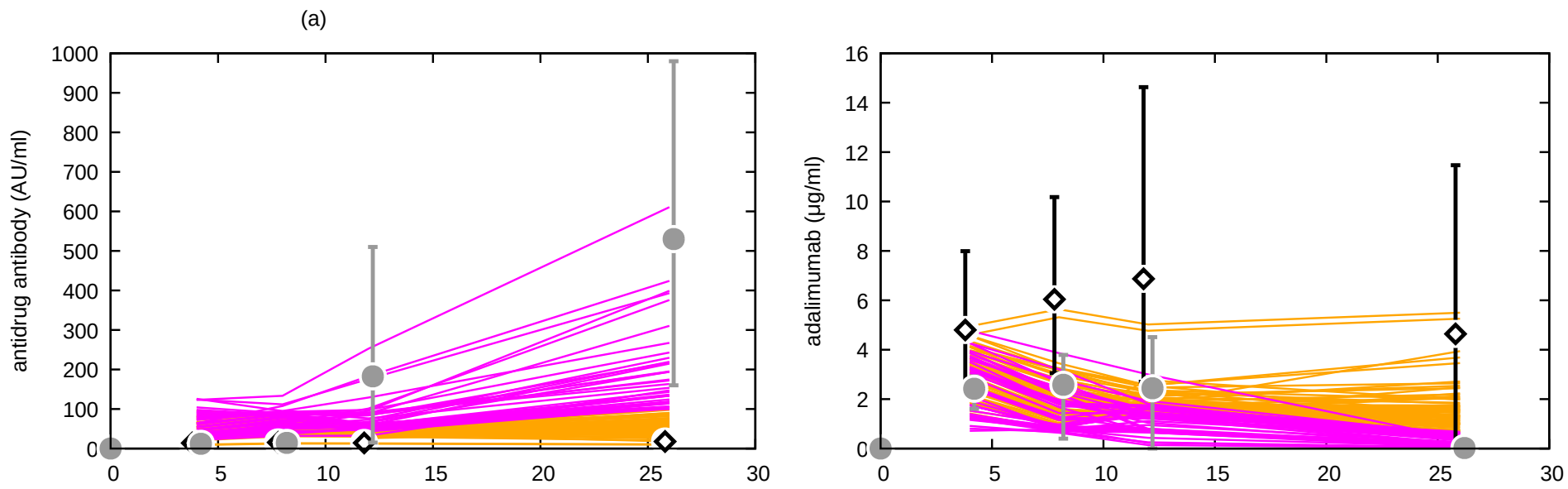

Fig I: Profiles of antidrug antibody and adalimumab for MTX+ virtual patients from ADA-low cohort (orange) and ADA-high cohort (magenta) under scenario no. 28 (MTX effect on activated T lymphocytes only). Error bars represent minimum and maximum for 14 MTX+ ADA-low patients (black ◇), 3 MTX+ ADA-high patients (grey ●) in the CoMARIS data.

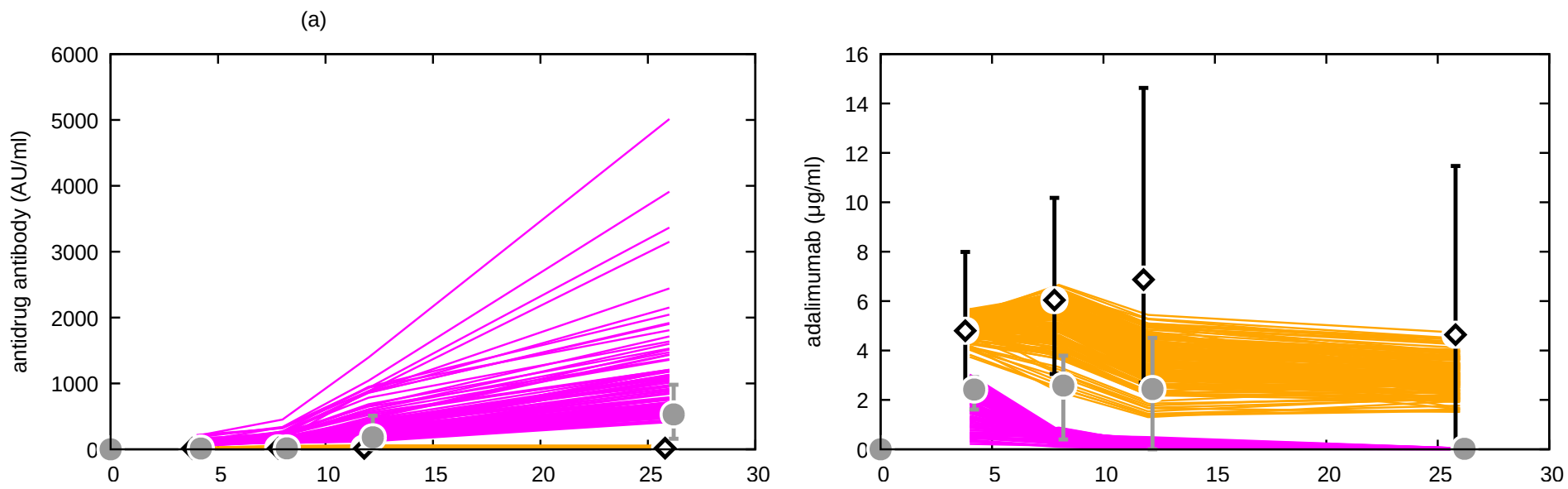

Fig J: Dynamics of antidrug antibody and adalimumab for MTX+ virtual patients from ADA-low cohort (orange) and ADA-high cohort (magenta) under scenario no. 29 (MTX effect on NK cells only). Error bars represent minimum and maximum for 14 MTX+ ADA-low patients (black ◇), 3 MTX+ ADA-high patients (grey ●) in the CoMARIS data.

E.3 Model-predicted cell dynamics

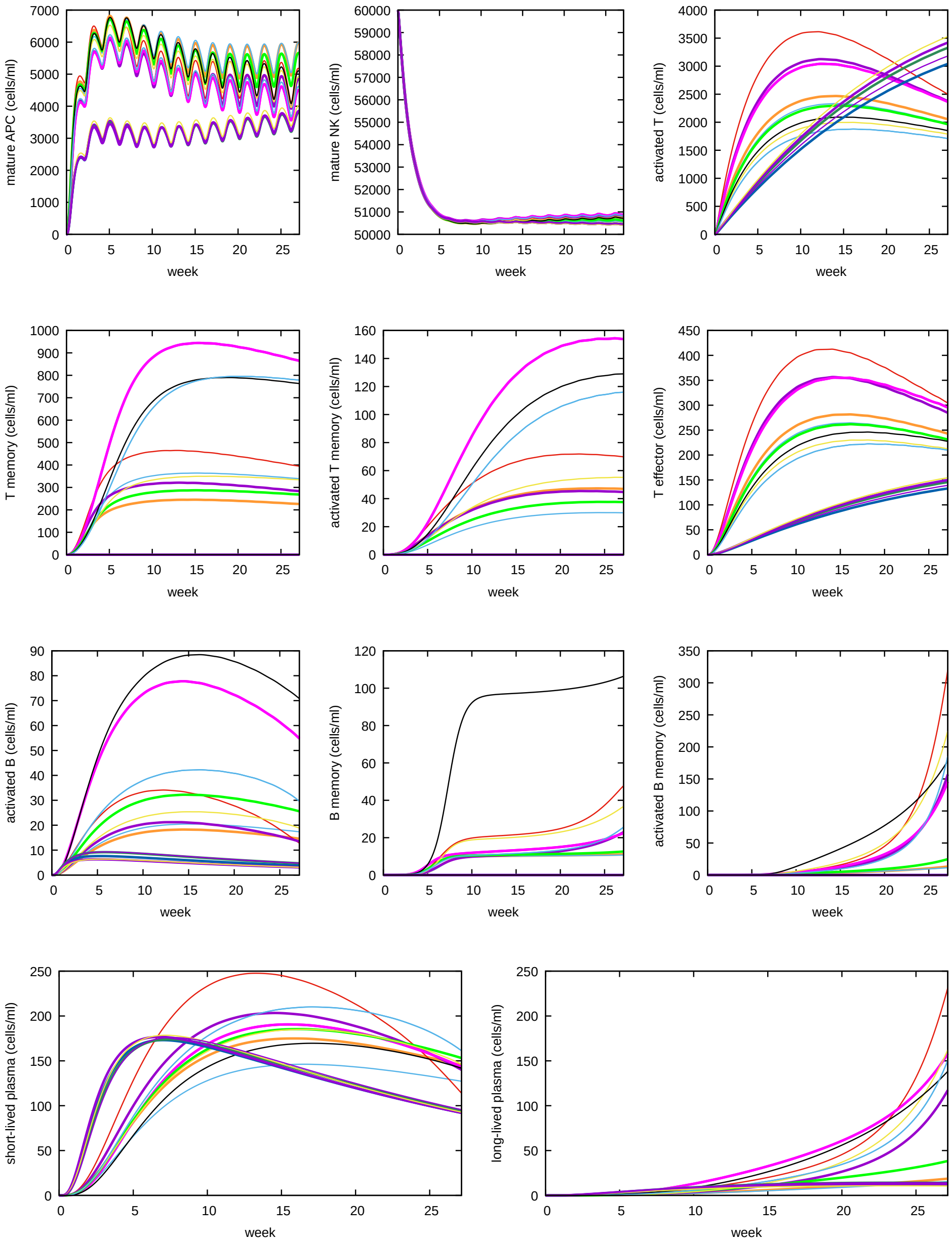

Fig K: Selected virtual MTX– patients from ADA-low cohort (including transient ADA). Trajectories use parameter values listed in Section D and drawn from LH15. The total simulated counts for activated CD4^{+} T cells, B cells, B memory cells, short-lived and long-lived plasma cells are in agreement with the totals reported in Figs A-C.

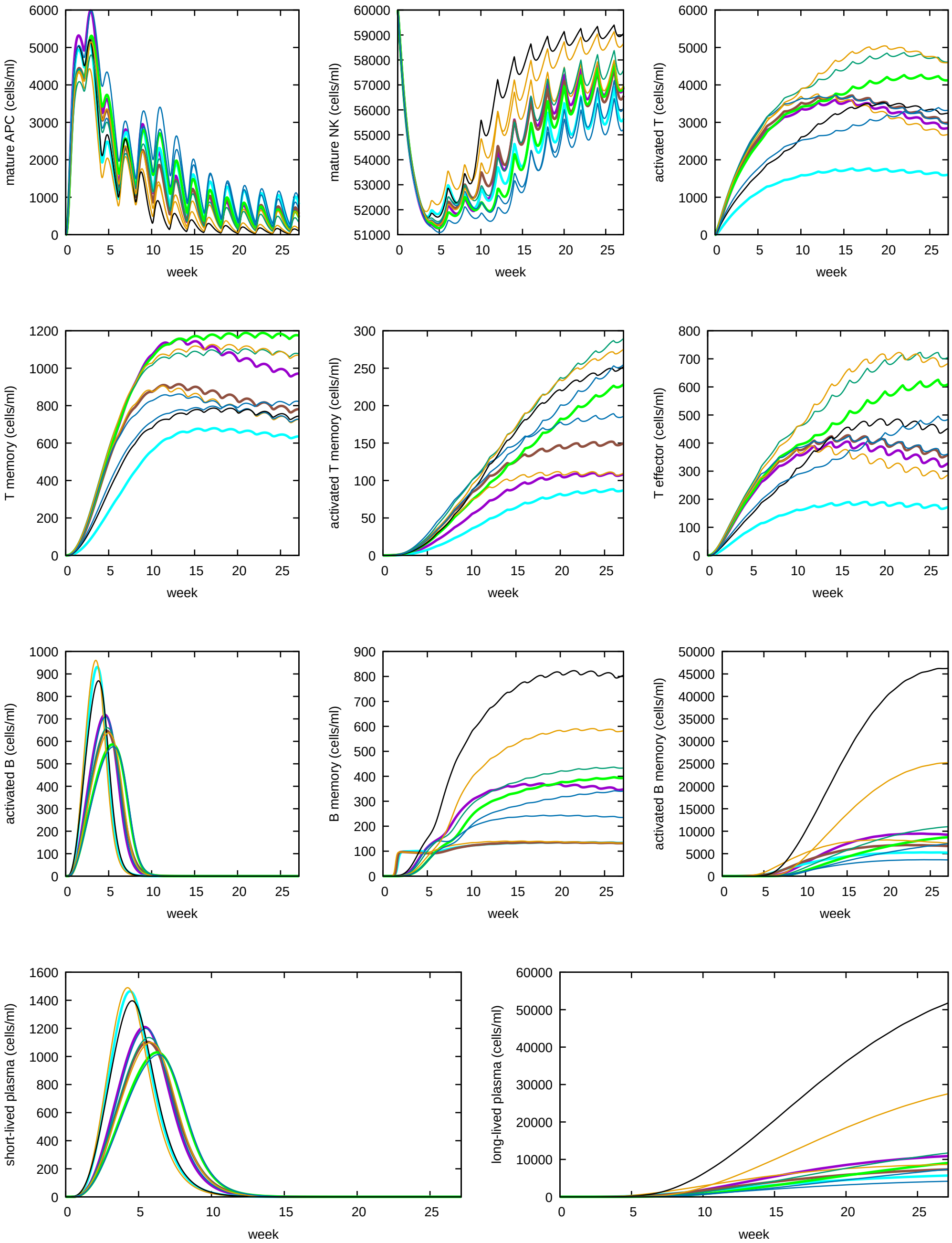

Fig L: Selected virtual MTX– patients from ADA-high cohort. Trajectories use parameter values listed in Section D and drawn from LH15. The total simulated counts for activated CD4^{+} T cells, B cells, B memory cells, short-lived and long-lived plasma cells are in agreement with the totals reported in Figs A-C.

E.4 MTX+ virtual patient cohort under scenario no. 28

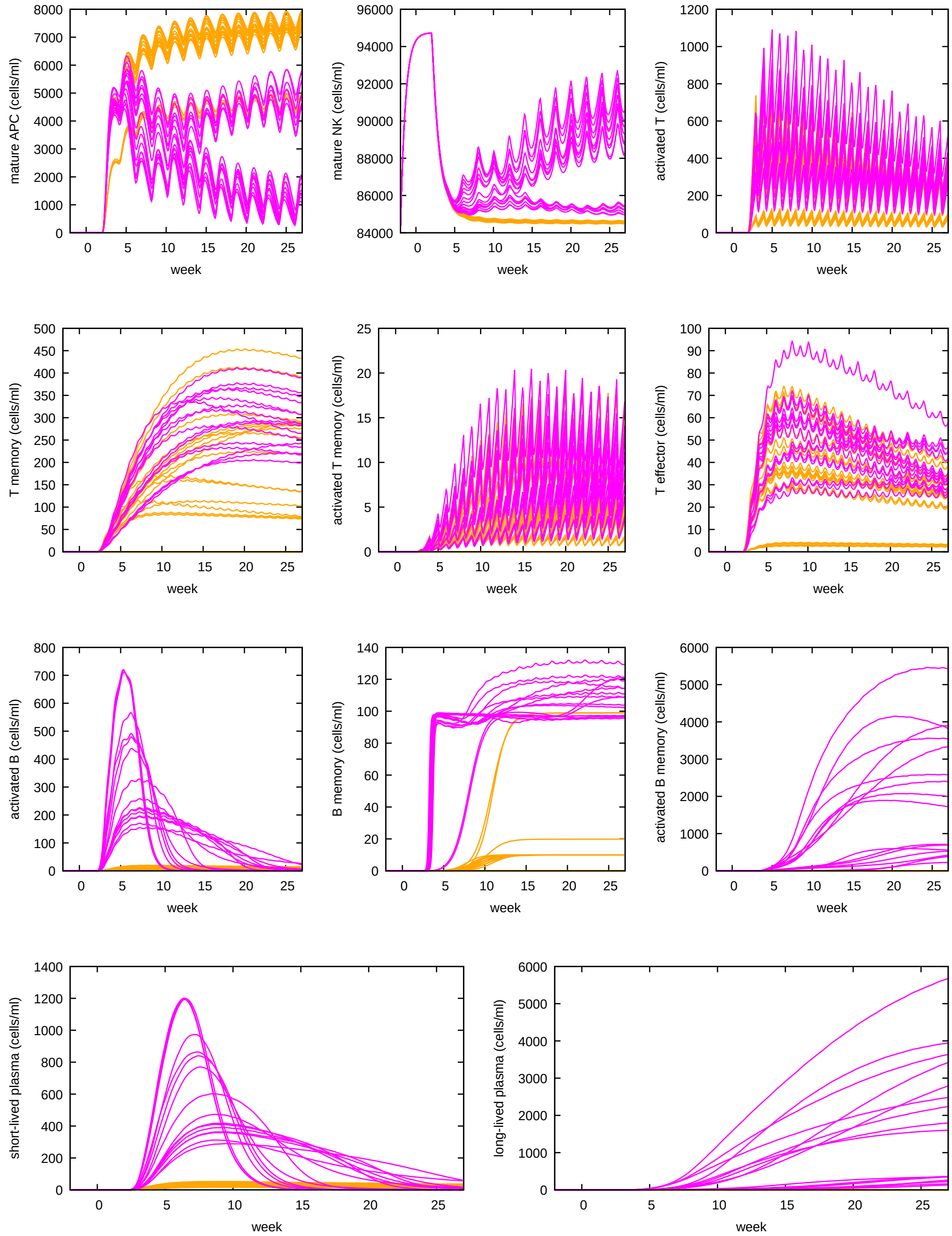

Fig M: Selected virtual MTX+ patients from ADA-low (orange) and ADA-high (magenta) cohort under scenario no. 28 ($D_X = 10$ mg/mL). Trajectories use parameter values listed in Section D and drawn from LH15. The total simulated counts for activated $CD4^+$ T cells, B cells, B memory cells, short-lived and long-lived plasma cells are in agreement with the totals reported in Figs A-C.

E.5 MTX+ virtual patient cohort under scenario no. 13

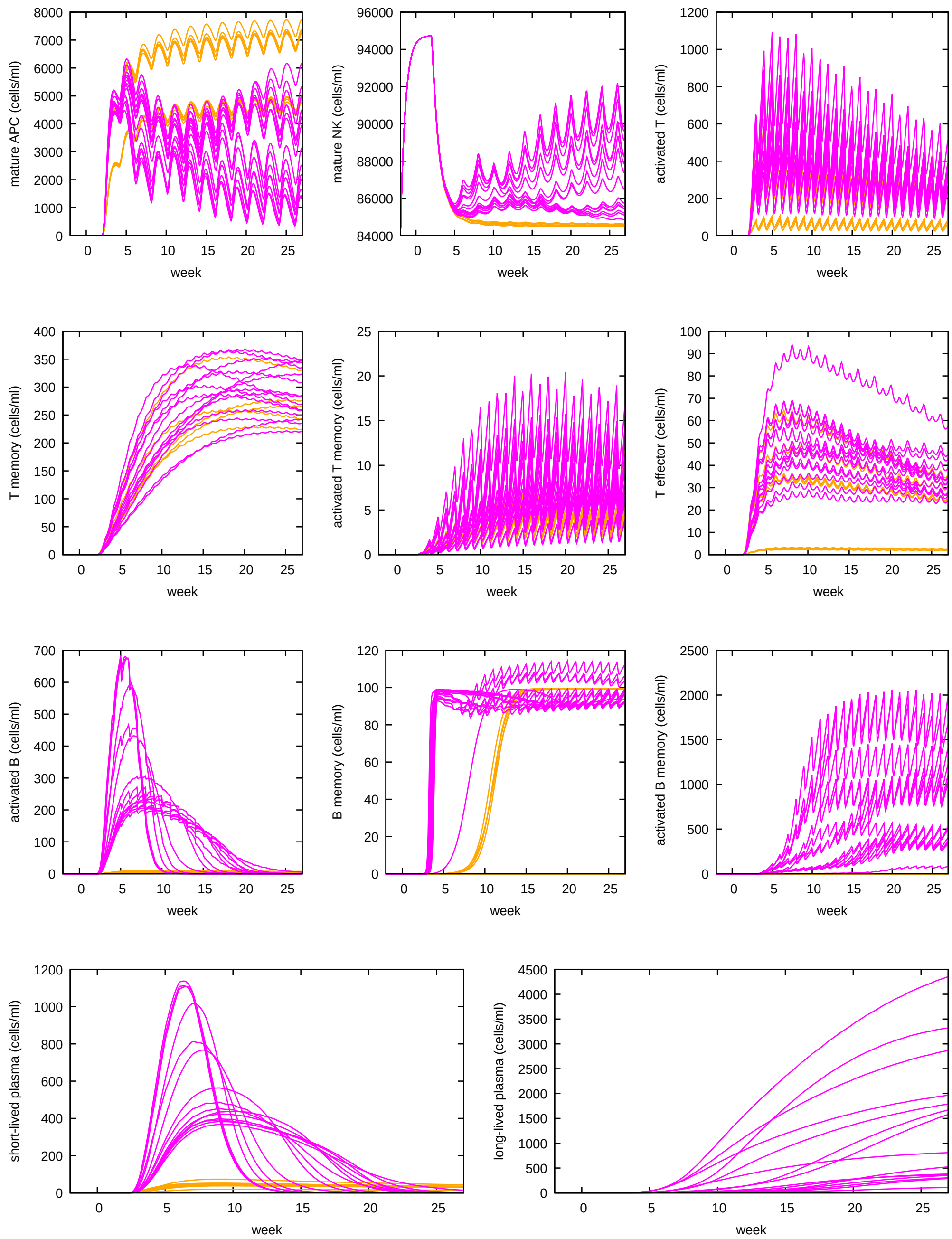

Fig N: Selected virtual MTX+ patients from ADA-low (orange) and ADA-high (magenta) cohort under scenario no. 13 ($D_X = 10$ mg/mL). Trajectories use parameter values listed in Section D and drawn from LH15. The total simulated counts for activated $CD4^+$ T cells, B cells, B memory cells, short-lived and long-lived plasma cells are in agreement with the totals reported in Figs A-C.

E.6 MTX+ virtual patient cohort under scenario no. 21

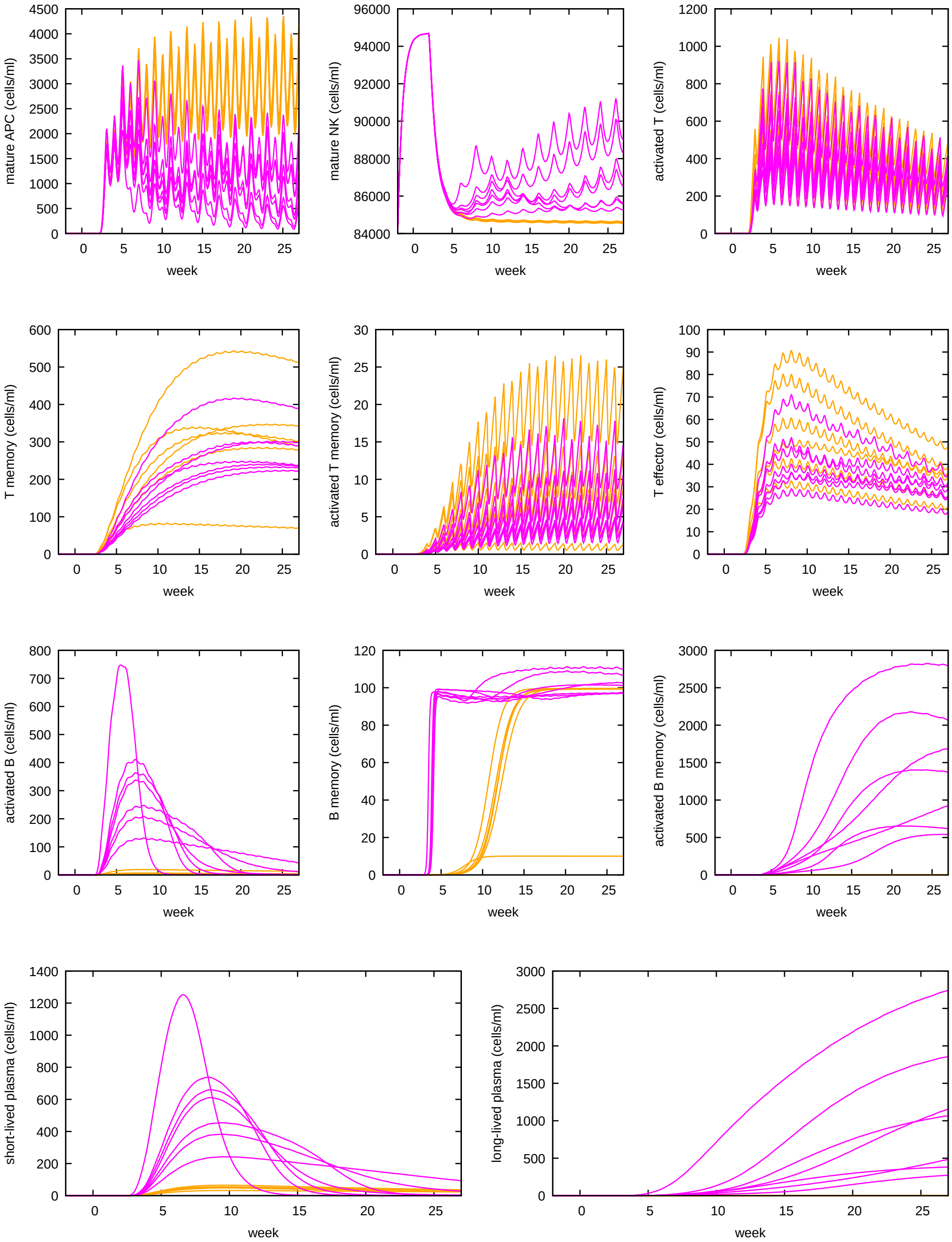

Fig O: Selected virtual MTX+ patients from ADA-low (orange) and ADA-high (magenta) cohort under scenario no. 21 ($D_X = 10$ mg/mL). Trajectories use parameter values listed in Section D and drawn from LH15. The total simulated counts for activated CD4$^+$ T cells, B cells, B memory cells, short-lived and long-lived plasma cells are in agreement with the totals reported in Figs A-C.

E.7 MTX+ virtual patient cohort under scenario no. 25

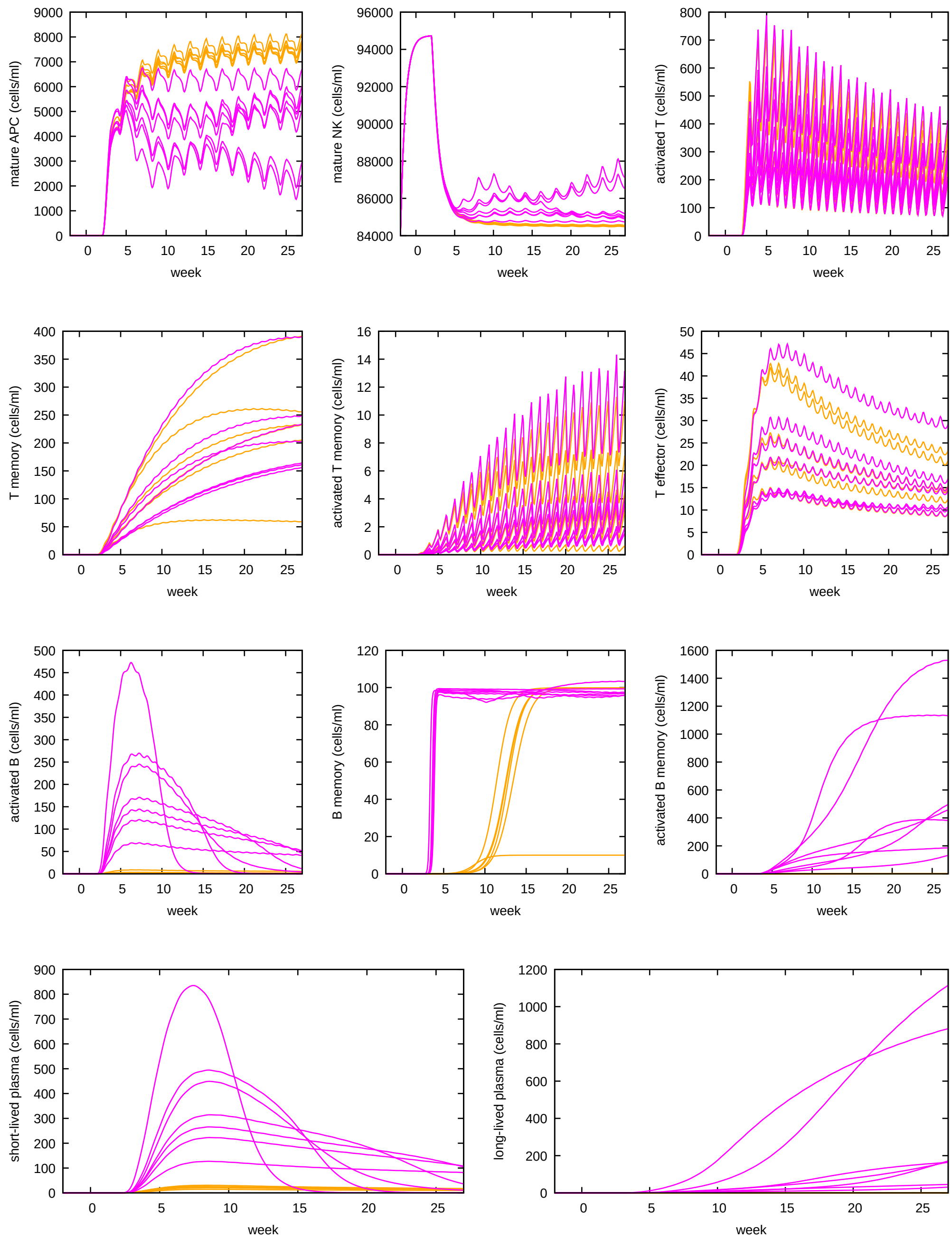

Fig P: Selected virtual MTX+ patients from ADA-low cohort under scenario no. 25 (D_X = 10 mg/mL). Trajectories use parameter values listed in Section D and drawn from LH15. The total simulated counts for activated CD4$^+$ T cells, B cells, B memory cells, short-lived and long-lived plasma cells are in agreement with the totals reported in Figs A-C.

E.8 MTX+ virtual patient cohort under scenario no. 27

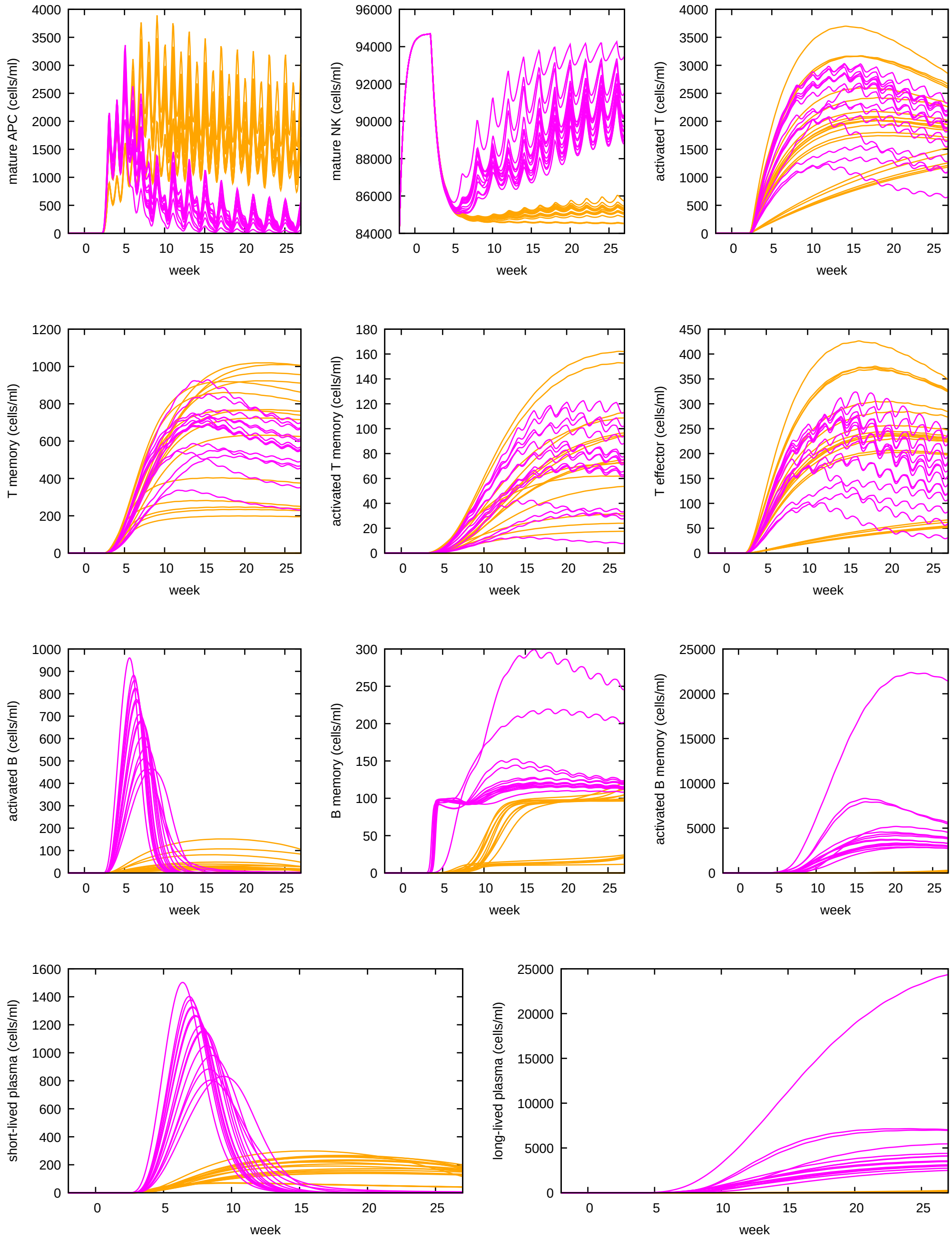

Fig Q: Selected virtual MTX+ patients from ADA-low (orange) and ADA-high (magenta) cohort under scenario no. 27 ($D_X = 10$ mg/mL). Trajectories use parameter values listed in Section D and drawn from LH15. The total simulated counts for activated $CD4^+$ T cells, B cells, B memory cells, short-lived and long-lived plasma cells are in agreement with the totals reported in Figs A-C.

E.9 MTX+ virtual patient cohort under scenario no. 29

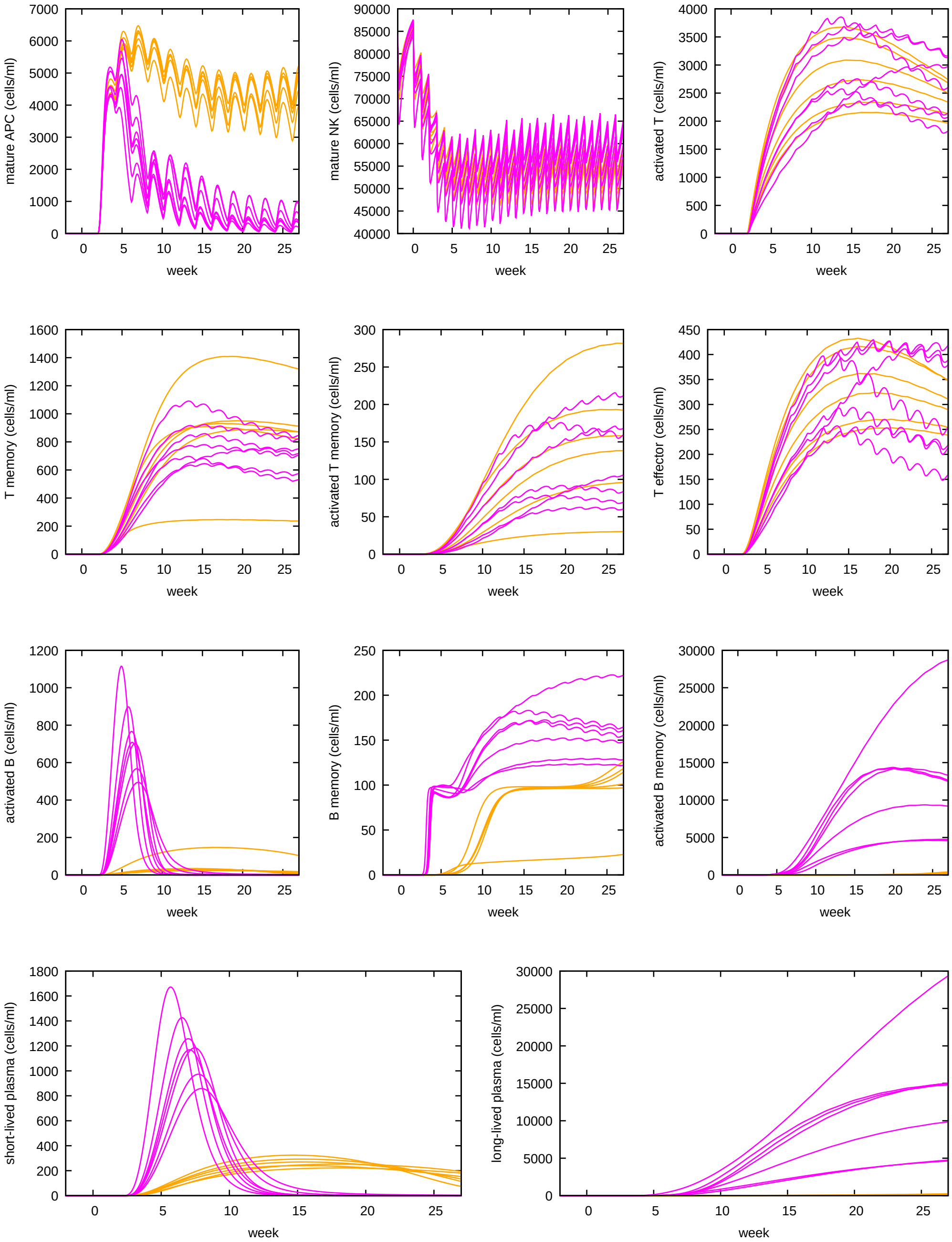

Fig R: Selected virtual MTX+ patients from ADA-low (orange) and ADA-high (magenta) cohort under scenario no. 29 ($D_X = 10$ mg/mL). Trajectories use parameter values listed in Section D and drawn from LH15. The total simulated counts for activated $CD4^+$ T cells, B cells, B memory cells, short-lived and long-lived plasma cells are in agreement with the totals reported in Figs A-C.

E.10 Simulations for delayed introduction of MTX

All trajectories plotted in this Section use parameter values listed in Section D and represent virtual patients (ADA-low or ADA-high cohort) drawn from LH15.

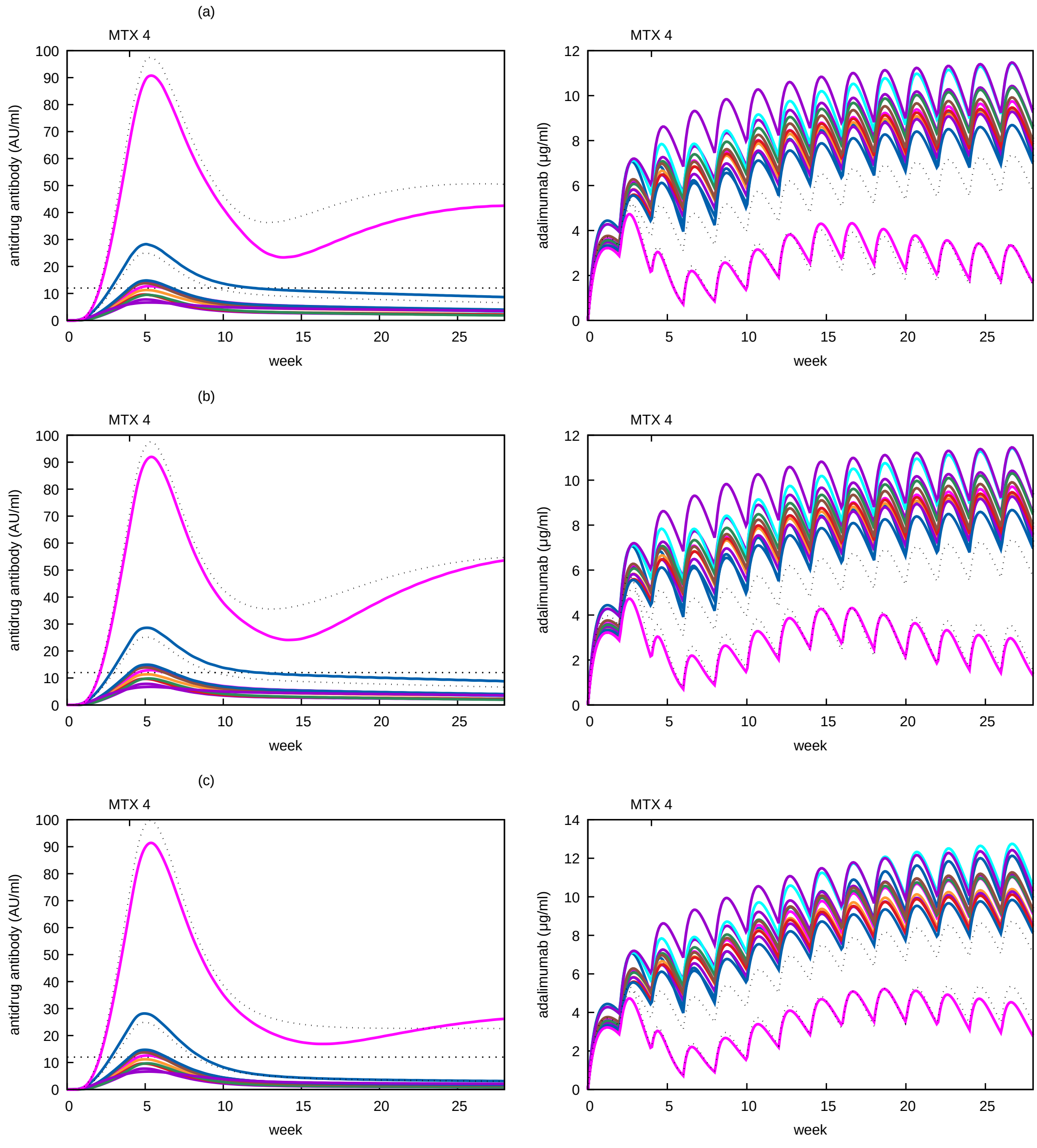

Fig S: Simulations of the temporal dynamics of ADA titre and adalimumab for selected virtual patients from the ADA-low cohort under scenarios (a) no. 13 , (b) no. 21, (c) no. 25, with co-administration of MTX ($D_X = 10$ mg/mL) starting in week 4.

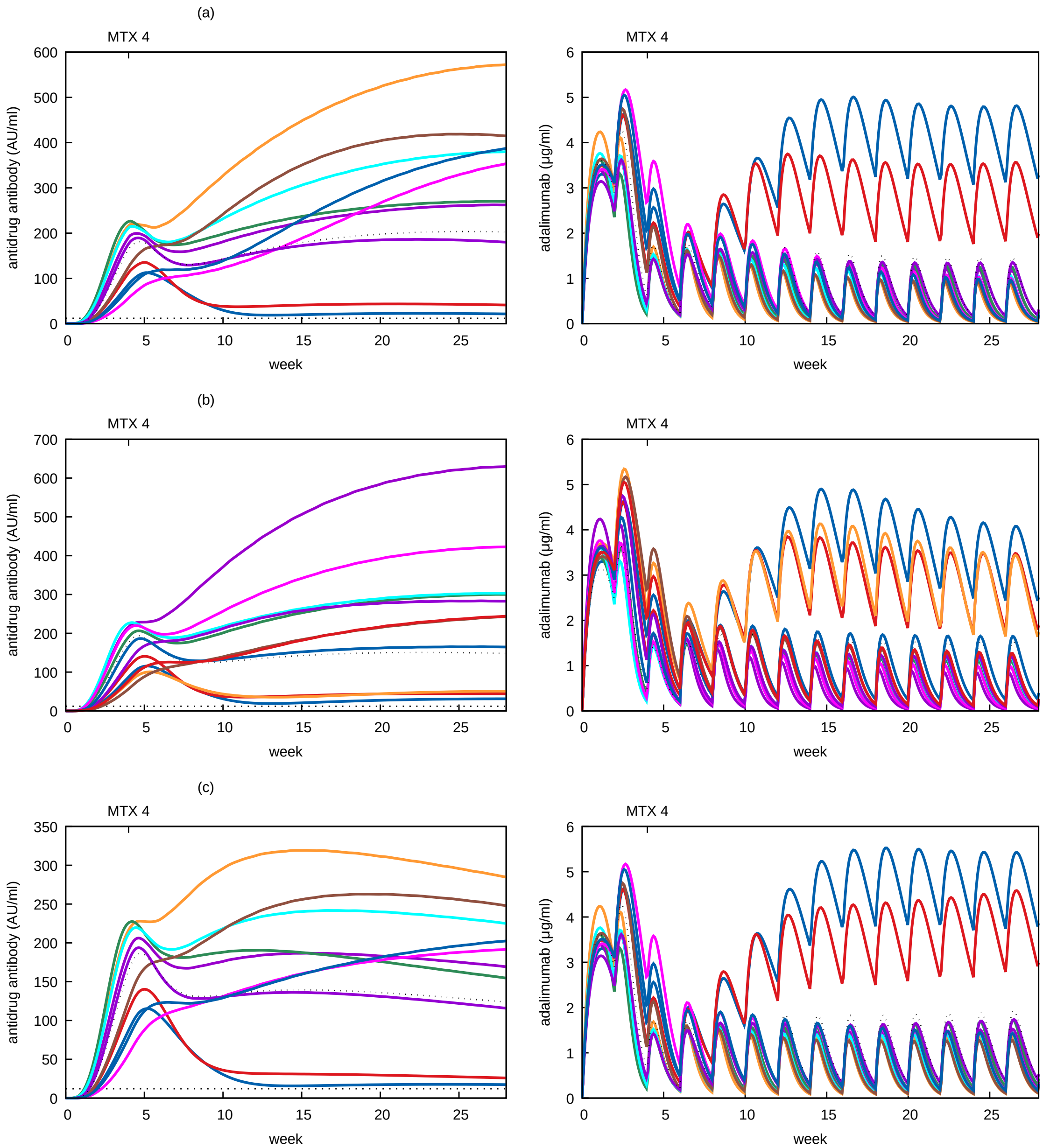

Fig T: Simulations of the temporal dynamics of ADA titre and adalimumab for virtual patients from the ADA-high cohort under scenarios (a) no. 13 , (b) no. 21, (c) no. 25, with co-administration of MTX ($D_X = 10$ mg/mL) starting in week 4.

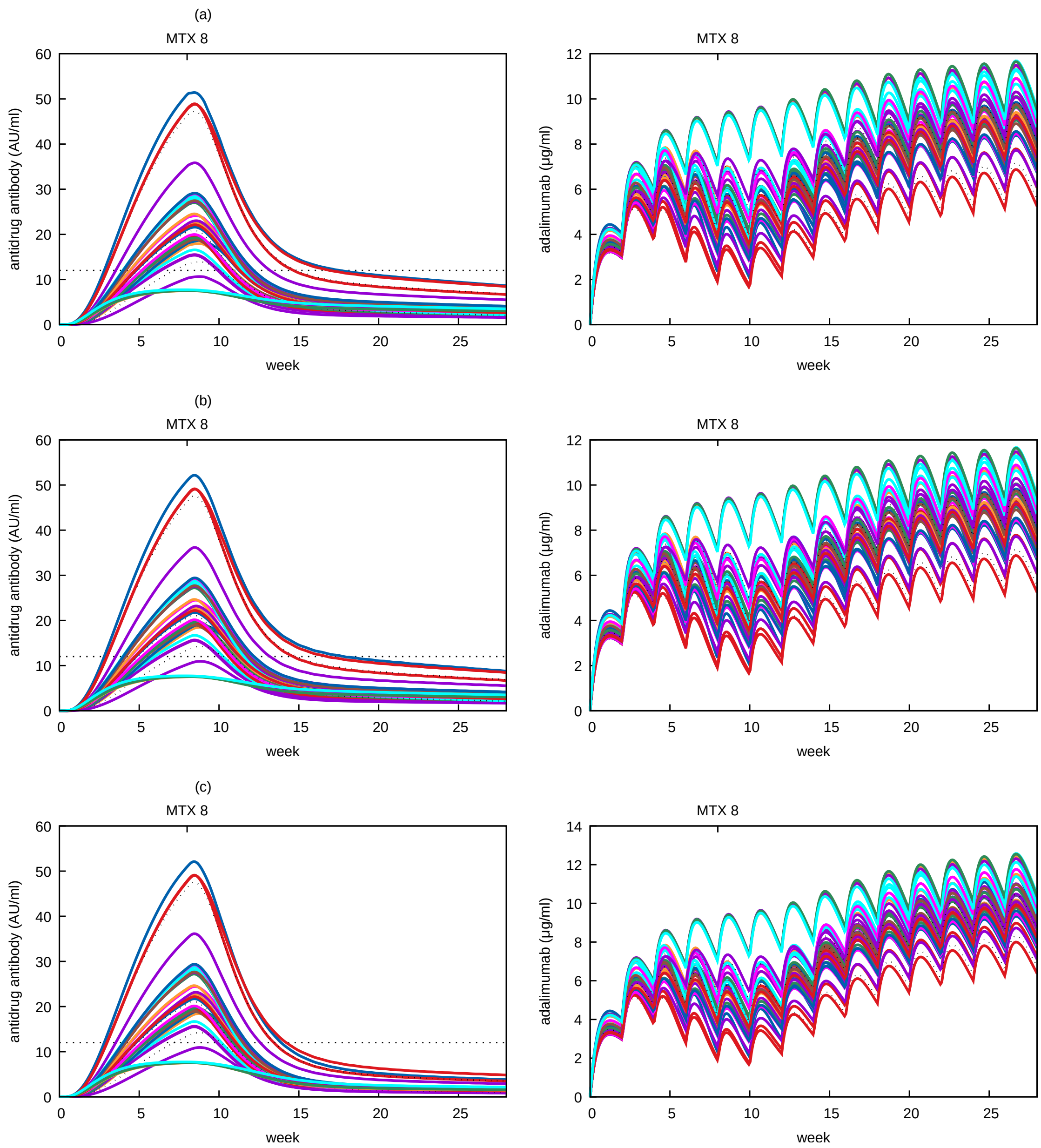

Fig U: Simulations of the temporal dynamics of ADA titre for virtual patients from the ADA-low cohort under scenarios (a) no. 13 , (b) no. 21, (c) no. 25, with co-administration of MTX ($D_X = 10$ mg/mL) starting in week 8.

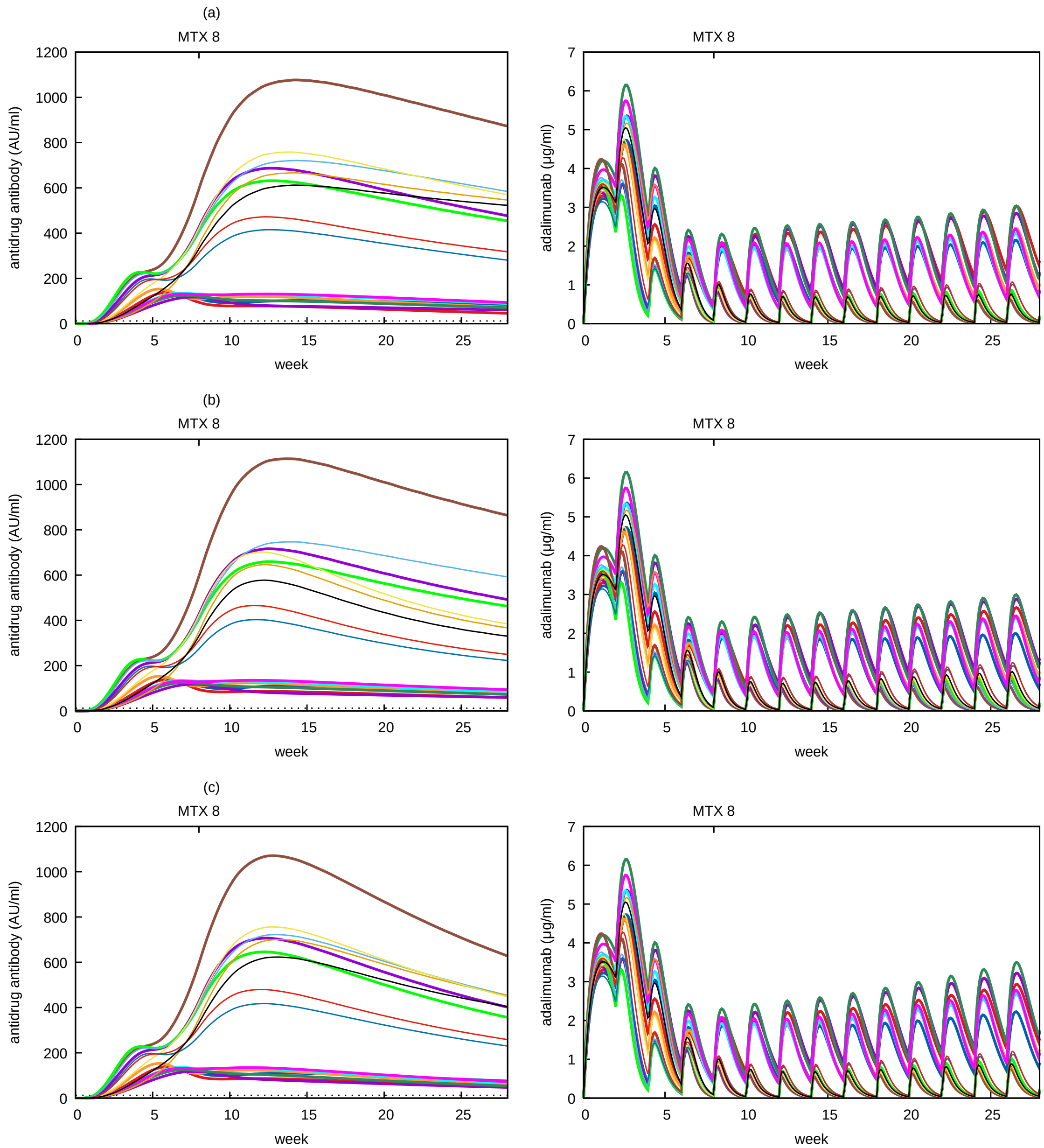

Fig V: Simulations of the temporal dynamics of ADA titre for virtual patients from the ADA-high cohort under scenarios (a) no. 13 , (b) no. 21, (c) no. 25, with co-administration of MTX ($D_X = 10$ mg/mL) starting in week 8.

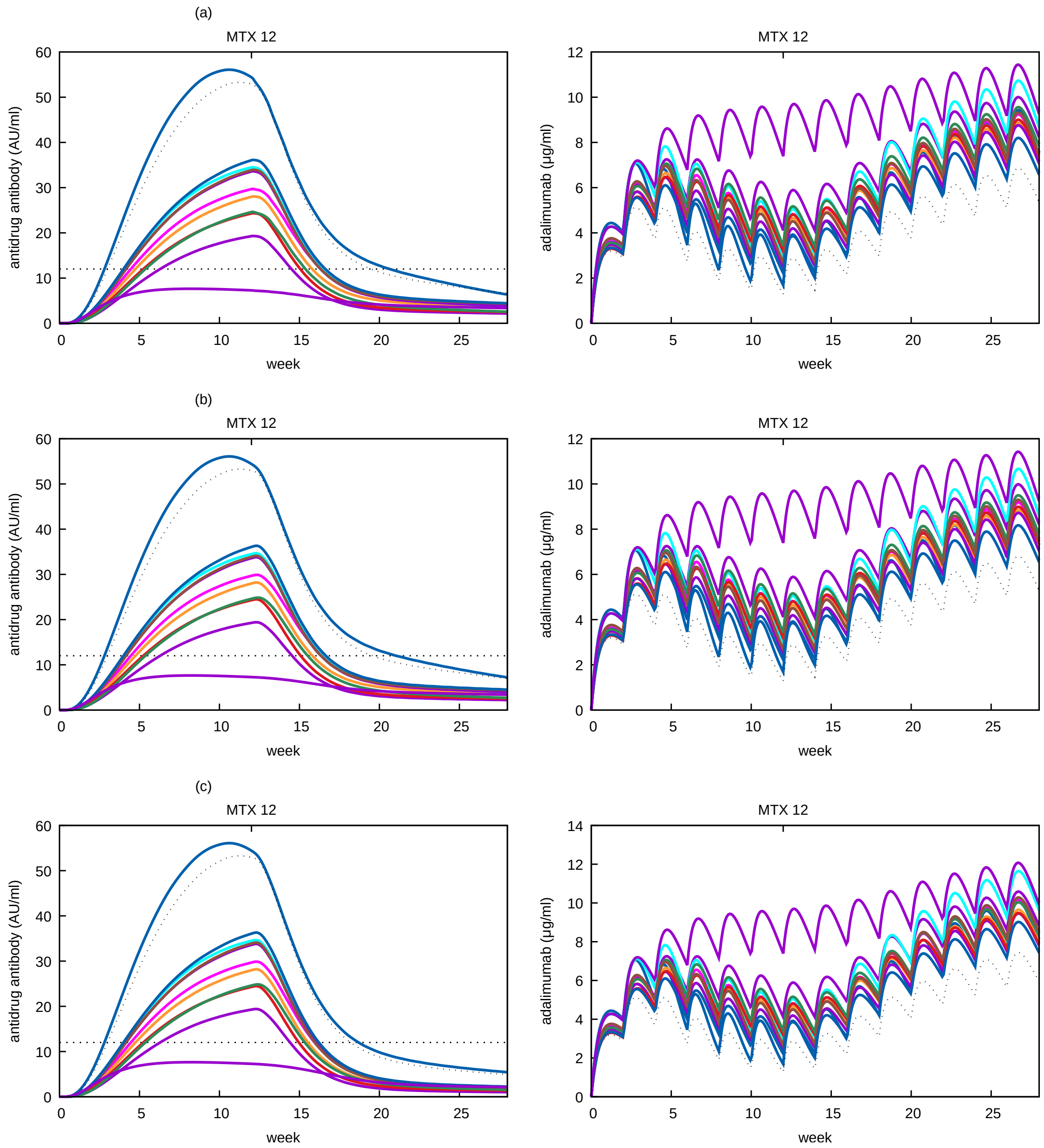

Fig W: Simulations of the temporal dynamics of ADA titre for virtual patients from the ADA-low cohort under scenarios (a) no. 13 , (b) no. 21, (c) no. 25, with co-administration of MTX ($D_X = 10$ mg/mL) starting in week 12.

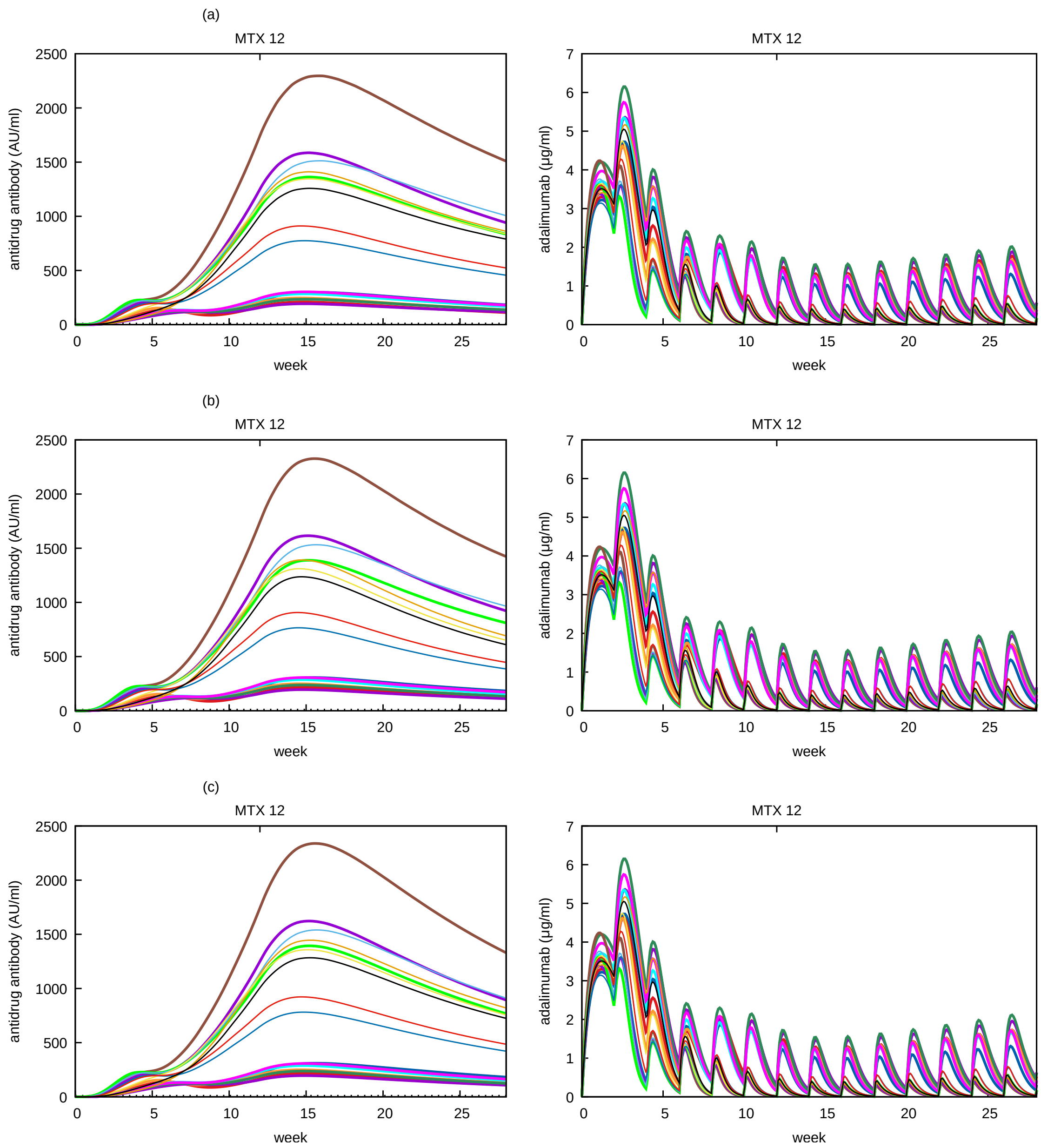

Fig X: Simulations of the temporal dynamics of ADA titre for virtual patients from the ADA-high cohort under scenarios (a) no. 13 , (b) no. 21, (c) no. 25, with co-administration of MTX ($D_X = 10$ mg/mL) starting in week 12.

E.11 Simulations of MTX- patients over a period of 52 weeks

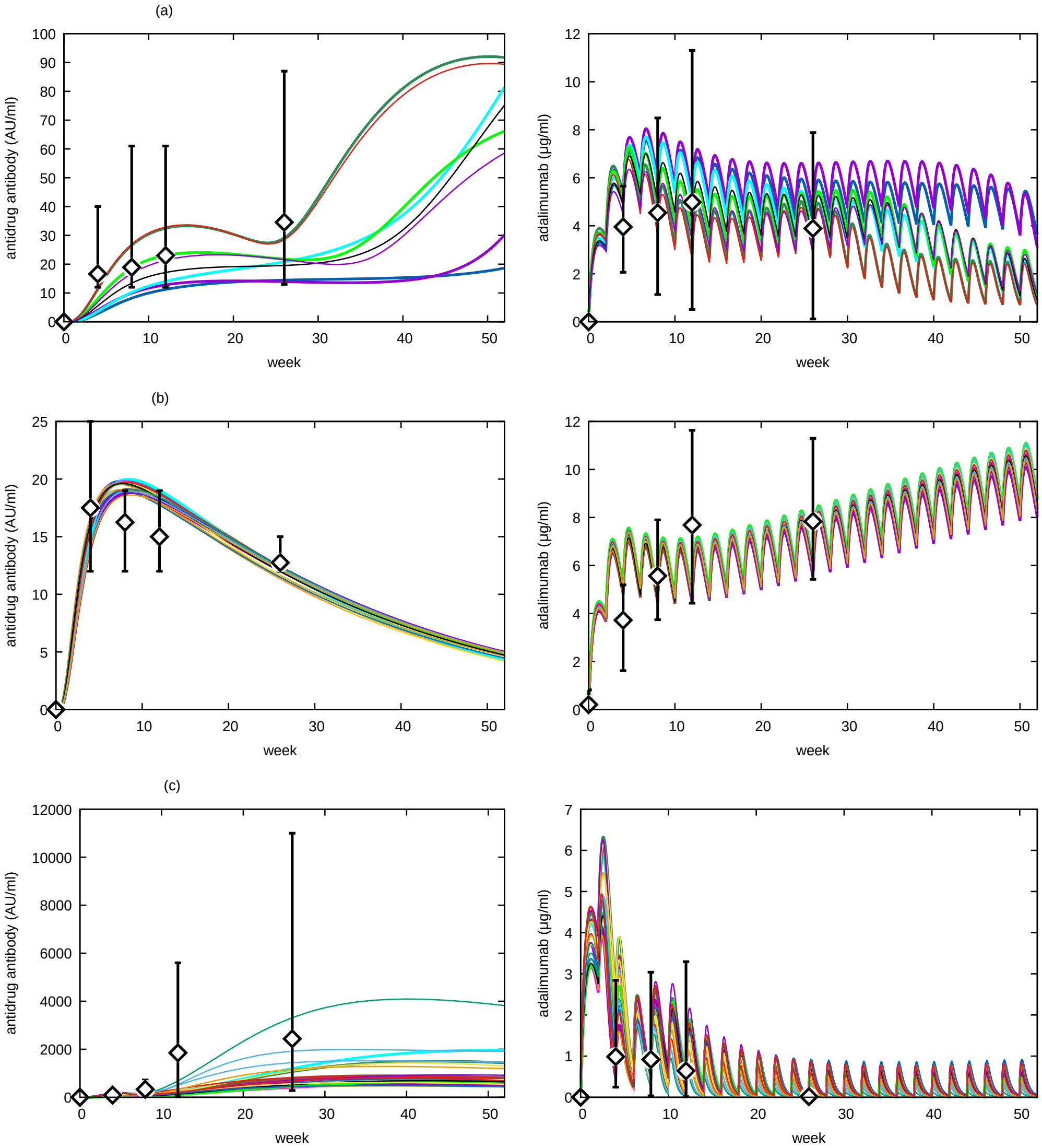

Fig Y: Selected virtual MTX– patients from ADA-low (a), transient ADA (b), and ADA-high (c) cohort. Error bars represent minimum and maximum of CoMARIS patient data (16 patients (a); 3 patients (b); 7 patients (c)). Trajectories use parameter values listed in Section D and drawn from LH15.

E.12 Conditional probabilities

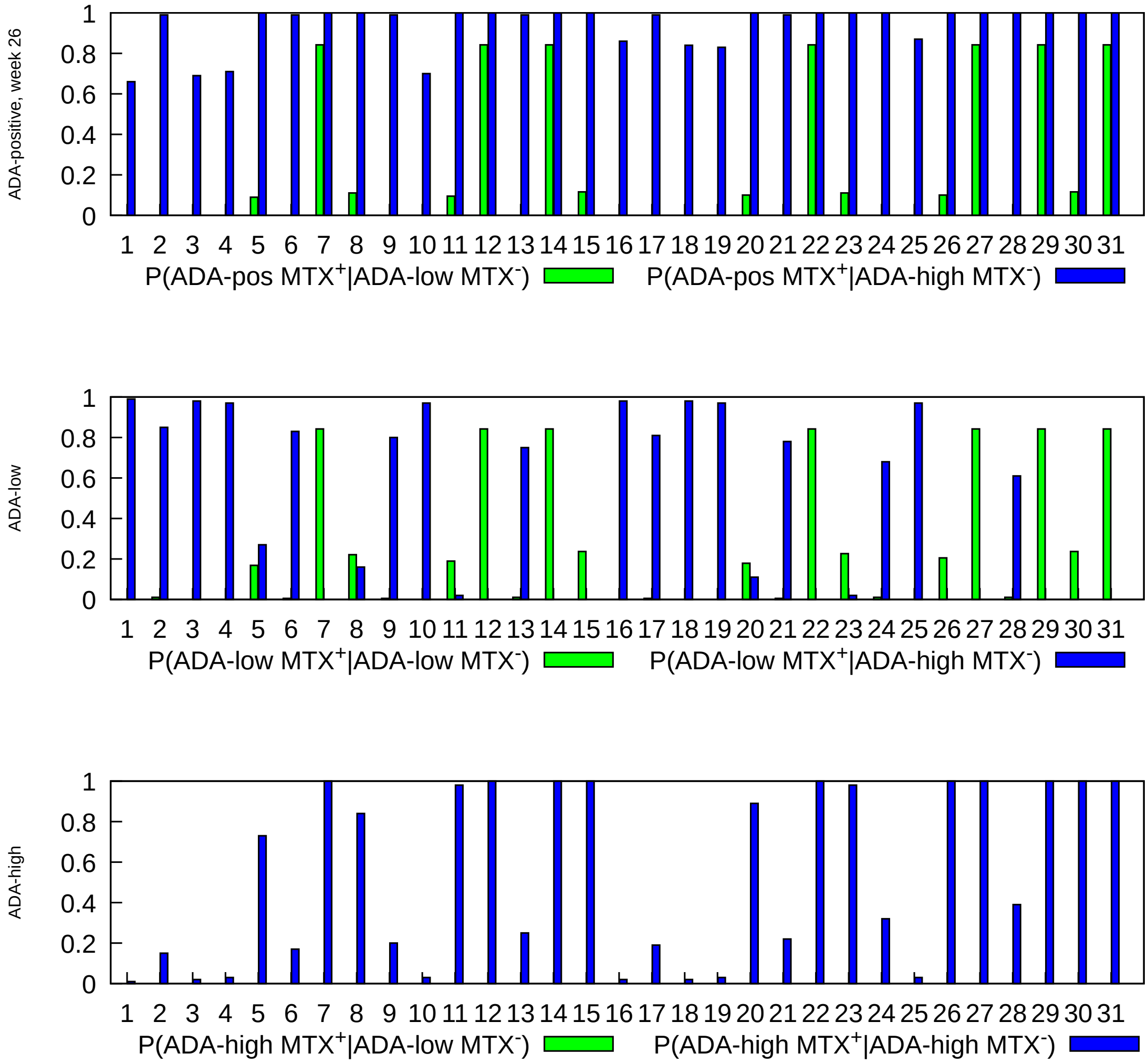

Fig Z: Conditional probabilities for the virtual patients drawn from LH15. Horizontal axis: list of scenarios.

References

[1] S. Atiqi, F. Hooijberg, F. Loeff, T. Rispens, and G. Wolbink. Immunogenicity of TNF-inhibitors. *Front. Immunol.*, 11:312, 2020. doi: 10.3389/fimmu.2020.00312.

[2] N. Azuz-Lieberman, G. Markel, S. Mizrahi, R. Gazit, J. Hanna, H. Achdout, R. Gruda, G. Katz, T. Arnon, S. Battat, E. Zamir, M. Adawi, R. Mader, and O. Mandelboim. The involvement of NK cells in ankylosing spondylitis. *Int. Immunol.*, 17(7):837–845, 2005. doi: 10.1093/intimm/dxh270.

[3] B. Bannwarth, F. Péhourcq, T. Schaeverbeke, and J. Dehais. Clinical pharmacokinetics of low-dose pulse methotrexate in rheumatoid arthritis. *Clin. Pharmacokinet.*, 30:194–210, 1996. doi: 10.2165/00003088-199630030-00002.

[4] G. M. Bartelds, C. L. Krieckaert, M. T. Nurmohamed, P. A. van Schouwenburg, W. F. Lems, J. W. Twisk, B. A. Dijkmans, L. Aarden, and G. J. Wolbink. Development of antidrug antibodies against adalimumab and association with disease activity and treatment failure during long-term follow-up. *J. Am. Med. Assoc.*, 305 (14):1460–1468, 2011. doi: 10.1001/jama.2011.406.

[5] K. P. Burnham and D. R. Anderson, editors. *Model Selection and Multimodel Inference*. Springer New York, NY, 2002. doi: 10.1007/b97636.

[6] X. Chen, T. Hickling, and P. Vicini. A mechanistic, multiscale mathematical model of immunogenicity for therapeutic proteins. *CPT Pharmacometrics Syst. Pharmacol.*, 3:e134, 2014. doi: 10.1038/psp.2014.31.

[7] C. I. Daien, S. Gailhac, T. Mura, R. Audo, B. Combe, M. Hahne, and J. Morel. Regulatory B10 cells are decreased in patients with rheumatoid arthritis and are inversely correlated with disease activity. *Arthritis Rheumatol.*, 66(8):2037–2046, 2014. doi: 10.1002/art.38666.

[8] H. Deveci, A. Turk, Z. Ozmen, A. Demir, and S. Say Coskun. Biological and genetic evaluation of il-23/il-17 pathway in ankylosing spondylitis patients. *Cent. Eur. J. Immunol.*, 44(4):433–439, 2019. doi: 10.5114/ceji.2019.92805.

[9] E. Dombrecht, N. Aerts, A. Schuerwegh, M. Hagendorens, D. Ebo, J. Van Offel, C. Bridts, W. Stevens, and L. De Clerck. Influence of anti-tumor necrosis factor therapy (adalimumab) on regulatory T cells and dendritic cells in rheumatoid arthritis. *Clin Exp Rheumatol*, 24(1):31—37, 2006.

[10] E. Ducourau, T. Rispens, M. Samain, E. Dernis, F. L. Guilchard, L. Andras, A. Perdriger, E. Lespessailles, A. Martin, G. Cormier, T. Armingeat, V. Devauchelle-Pensec, E. Gervais, B. L. Goff, A. de Vries, E. Piver, G. Paintaud, C. Desvignes, D. Ternant, H. Watier, P. Goupille, and D. Mulleman. Methotrexate effect on immunogenicity and long-term maintenance of adalimumab in axial spondyloarthritis: a multicentric randomised trial. *RMD Open*, 6(1):e001047, 2020. doi: 10.1136/rmdopen-2019-001047.

[11] J. Fecteau, A. Roy, and S. Néron. Peripheral blood cd27$^+$igg$^+$ B cells rapidly proliferate and differentiate into immunoglobulin-secreting cells after exposure to low CD154interaction. *Immunology*, 128(1pt2):e353–e365, 2009. doi: 10.1111/j.1365-2567.2008.02976.x.

[12] L. Genestier, R. Paillot, S. Fournel, C. Ferraro, P. Miossec, and J. P. Revillard. Immunosuppressive properties of methotrexate: apoptosis and clonal deletion of activated peripheral T cells. *J. Clin. Invest.*, 102(2):322–328, 1998. doi: 10.1172/JCI2676.

[13] J. Gratacós, A. Collado, X. Filella, R. Sanmartí, J. Cañete, J. Llena, R. Molina, A. Ballesta, and J. Muñoz Gómez. Serum cytokines (IL-6, TNF-α, IL-1β and IFN-γ) in ankylosing spondylitis: a close correlation between serum IL-6 and disease activity and severity. *Rheumatology*, 33(10):927–931, 10 1994. ISSN 1462-0324. doi: 10.1093/rheumatology/33.10.927.

[14] Y. Ha, D. Topham, S. Park, J. Hollenbaugh, J. Treanor, T. Mosmann, X. Jin, B. Ward, H. Miao, J. Holden-White, A. Perelson, M. Zand, and H. Wu. Simulation and prediction of the adaptive immune response to influenza A virus infection. *J. Virology*, 83:7151–7165, 2009. doi: 10.1128/JVI.00098-09.

[15] J. Haller Hasskamp, J. Zapas, and E. Elias. Dendritic cell counts in the peripheral blood of healthy adults. *Am. J. Hematol.*, 78(4):314–315, 2005. doi: 10.1002/ajh.20296.

[16] R. A. Herman, P. Veng-Pedersen, J. Hoffman, R. Koehnke, and D. E. Furst. Pharmacokinetics of low-dose methotrexate in rheumatoid arthritis patients. *J. Pharm. Sci.*, 78(2):165–171, 1989. ISSN 0022-3549. doi: 10.1002/jps.2600780219.

[17] T. Hibi and H.-M. Dosch. Limiting dilution analysis of the B cell compartment in human bone marrow. *Eur. J. Immunol.*, 16(2):139–145, 1986. doi: 10.1002/eji.1830160206.

[18] M. Hoekstra, C. Haagsma, C. Neef, J. Proost, A. Knuif, and M. van de Laar. Bioavailability of higher dose methotrexate comparing oral and subcutaneous administration in patients with rheumatoid arthritis. *J. Rheumatol.*, 31(4):645–648, 2004. ISSN 0315-162X.

[19] S. Jain, B. Laishangtham, A. Khullar, B. Saikia, and V. Dhir. Peripheral blood and synovial fluid natural killer (NK) and natural killer T (NKT) cell immunophenotypes in active rheumatoid athritis and their role as biomarkers of methotrexate response (abstract). *Arthritis Rheum.*, 74(suppl 9), 2022.

[20] H.-J. Jeong. Impact of methotrexate on immune response. Masters thesis, Seoul National University, Korea, 2025. URL `https://dcollection.snu.ac.kr/common/orgView/000000186797`.

[21] Z. Kaymakcalan, P. Sakorafas, S. Bose, S. Scesney, L. Xiong, D. K. Hanzatian, J. Salfeld, and E. Sasso. Comparisons of affinities, avidities, and complement activation of adalimumab, infliximab, and etanercept in binding to soluble and membrane

tumor necrosis factor. *J Clin Immunol*, 131(2):308–316, 2009. ISSN 1521-6616. doi: 10.1016/j.clim.2009.01.002.

[22] L. Khodadadi, Q. Cheng, A. Radbruch, and F. Hiepe. The maintenance of memory plasma cells. *Front. Immunol.*, 10:721, 2019. doi: 10.3389/fimmu.2019.00721.

[23] G. Krikorian, W. Marshall, S. Simmons, and F. Stratton. Counts and characteristics of macrophage precursors in human peripheral blood. *Cell. Immunol.*, 19(1):22–31, 1975. ISSN 0008-8749. doi: 10.1016/0008-8749(75)90288-9.

[24] D. Macallan, B. Asquith, A. Irvine, D. Wallace, A. Worth, H. Ghattas, Y. Zhang, G. Griffin, D. Tough, and P. Beverley. Measurement and modeling of human T cell kinetics. *Eur. J. Immunol.*, 33(8):2316–2326, 2003. doi: 10.1002/eji.200323763.

[25] D. Macallan, D. Wallace, Y. Zhang, C. de Lara, A. Worth, H. Ghattas, G. E. Griffin, P. Beverley, and D. Tough. Rapid turnover of effector–memory CD4+ T cells in healthy humans. *J. Exp. Med.*, 200(2):255–260, 2004. doi: 10.1084/jem.20040341.

[26] D. Macallan, D. Wallace, Y. Zhang, H. Ghattas, B. Asquith, C. de Lara, A. Worth, G. Panayiotakopoulos, G. Griffin, D. Tough, and P. L. Beverley. B-cell kinetics in humans: rapid turnover of peripheral blood memory cells. *Blood*, 105(9):3633–3640, 2005. doi: 10.1182/blood-2004-09-3740.

[27] A. A. Patel, F. Ginhoux, and S. Yona. Monocytes, macrophages, dendritic cells and neutrophils: an update on lifespan kinetics in health and disease. *Immunology*, 163: 250–261, 2021. doi: 10.1111/imm.13320.

[28] K. V. Rogers, S. W. Martin, I. Bhattacharya, R. S. P. Singh, and S. Nayak. A dynamic quantitative systems pharmacology model of inflammatory bowel disease: Part 2 – application to current therapies in Crohn's disease. *Clin. Transl. Sci.*, 14 (1):249–259, 2021. doi: 10.1111/cts.12850.

[29] G. Rosenthal, D. Germolec, K. Lamm, M. Ackermann, and M. Luster. Comparative effects on the immune system of methotrexate and trimetrexate. *Int. J. Immunopharmac.*, 9:793–801, 1987. doi: 10.1016/0192-0561(87)90075-0.

[30] M. Seitz, M. Zwicker, and P. Loetscher. Effects of methotrexate on differentiation of monocytes and production of cytokine inhibitors by monocytes. *Arthritis Rheum.*, 41(11):2032–2038, 1998. doi: 10.1002/1529-0131(199811)41:11<2032::AID-ART19>3.0.CO;2-J.

[31] G. Sigounas, N. Harindranath, G. Donadel, and A. L. Notkins. Half-life of polyreactive antibodies. *J. Clin. Immunol.*, 14(2):134–140, 1994. doi: 10.1007/BF01541346.

[32] K. Soetaert and T. Petzoldt. Inverse modelling, sensitivity and Monte Carlo analysis in R using package FME. *J. Stat. Softw.*, 33(3):1–28, 2010. doi: 10.18637/jss.v033.i03.

[33] B. Sonel, H. Tutkak, and N. Düzgün. Serum levels of IL-1beta, TNF-alpha, IL-8, and acute phase proteins in seronegative spondyloarthropathies. *Joint Bone Spine*, 69(5):463–467, 2002. ISSN 1297-319X. doi: 10.1016/S1297-319X(02)00431-1.

[34] D. Ternant, E. Ducourau, P. Fuzibet, C. Vignault, H. Watier, T. Lequerré, X. Le Loët, O. Vittecoq, P. Goupille, D. Mulleman, and G. Paintaud. Pharmacokinetics and concentration–effect relationship of adalimumab in rheumatoid arthritis. *Brit. J. Clin. Pharmaco.*, 79(2):286–297, 2015. doi: 10.1111/bcp.12509.

[35] L. Tucker, N. Yager, H. Al-Mossawi, P. Bowness, A. Cribbs, and L. Coates. E102 Transcriptomic effects of methotrexate on natural killer cells in psoriatic arthritis. *Rheumatology*, 64(Supplement_3):keaf142.337, 2025. doi: 10.1093/rheumatology/keaf142.337.

[36] S. J. Turley, K. Inaba, W. S. Garrett, M. Ebersold, J. Unternaehrer, R. M. Steinman, and I. Mellman. Transport of peptide-mhc class ii complexes in developing dendritic cells. *Science*, 288(5465):522–527, 2000. doi: 10.1126/science.288.5465.522.

[37] P. A. van Schouwenburg, G. M. Bartelds, M. H. Hart, L. Aarden, G. J. Wolbink, and D. Wouters. A novel method for the detection of antibodies to adalimumab in the presence of drug reveals "hidden" immunogenicity in rheumatoid arthritis patients. *J. Immunol. Methods*, 362(1):82–88, 2010. ISSN 0022-1759. doi: 10.1016/j.jim.2010.09.005.

[38] B. Vandooren, T. Noordenbos, C. Ambarus, S. Krausz, T. Cantaert, N. Yeremenko, M. Boumans, R. Lutter, P. Tak, and D. Baeten. Absence of a classically activated macrophage cytokine signature in peripheral spondylarthritis, including psoriatic arthritis. *Arthritis Rheum.*, 60(4):966–975, 2009. doi: 10.1002/art.24406.

[39] M. H. Weisman, L. W. Moreland, D. E. Furst, M. E. Weinblatt, E. C. Keystone, H. E. Paulus, L. S. Teoh, R. B. Velagapudi, P. A. Noertersheuser, G. Granneman, S. A. Fischkoff, and E. K. Chartash. Efficacy, pharmacokinetic, and safety assessment of adalimumab, a fully human anti-tumor necrosis factor-alpha monoclonal antibody, in adults with rheumatoid arthritis receiving concomitant methotrexate: A pilot study. *Clin. Ther.*, 25(6):1700–1721, 2003. doi: 10.1016/S0149-2918(03)80164-9.

[40] P. B. Wright, A. McEntegart, D. McCarey, I. B. McInnes, S. Siebert, and S. W. F. Milling. Ankylosing spondylitis patients display altered dendritic cell and T cell populations that implicate pathogenic roles for the IL-23 cytokine axis and intestinal inflammation. *Rheumatology*, 55(1):120–132, 08 2015. ISSN 1462-0324. doi: 10.1093/rheumatology/kev245.

[41] G. Zahn and A. Greischel. Pharmacokinetics of tumor necrosis factor alpha after intravenous administration in rats. dose dependence and influence of tumor necrosis factor beta. *Arzneimittelforschung*, 39(9):1180–2, 1989.

[42] Y. Zhang, D. Wallace, C. de Lara, H. Ghattas, B. Asquith, A. Worth, G. Griffin, G. Taylor, D. Tough, P. Beverley, and D. Macallan. In vivo kinetics of human natural killer cells: the effects of ageing and acute and chronic viral infection. *Immunology*, 121(2):258–265, 2007. doi: 10.1111/j.1365-2567.2007.02573.x.
